# Supplementary material for: Dissecting maternal and fetal genetic effects underlying the associations between maternal phenotypes, birth outcomes, and adult phenotypes: A mendelian-randomization and haplotype-based genetic score analysis in 10,734 mother–infant pairs
Source: PLoS Med. 2020 Aug 25;17(8):e1003305. doi: 10.1371/journal.pmed.1003305 (PMC7447062; doi:10.1371/journal.pmed.1003305)
Supplement: S1 Text — (PDF) [file pmed.1003305.s002.pdf]

Supplementary Method

Title

Dissecting maternal and fetal genetic effects underlying the associations between maternal phenotypes, birth outcomes, and adult phenotypes: A mendelian-randomization and haplotype-based genetic score analysis in 10,734 mother–infant pairs

Authors

Jing Chen, Jonas Bacelis, Pol Sole-Navais, Amit Srivastava, Julius Juodakis, Amy Rouse, Mikko Hallman, Kari Teramo, Mads Melbye, Bjarke Feenstra, Rachel M. Freathy, George Davey Smith, Deborah A. Lawlor, Jeffrey C. Murray, Scott M. Williams, Bo Jacobsson, Louis J. Muglia*, Ge Zhang*

* ge.zhang@cchmc.org or louis.muglia@cchmc.org

Contents

Contents 1

Supplementary Methods 2

Data sets 2

Phenotype data 4

Genotype data 5

Genetic scores 7

Statistical analyses 8

References 17

Supplementary Methods

Data sets

We used phenotype and genomewide SNP data of 10,734 mother/infant pairs from six birth studies collected from UK, Northern Europe, Australia, and North America (S1 Table).

FIN

The Finnish dataset (FIN) was collected for a genetic study of spontaneous preterm birth [1]. Briefly, whole blood samples were collected from more than 1,600 mother/child pairs from the Helsinki (southern Finland) University Hospitals between 2004 and 2014. All the studied samples are of Finnish descent. Crown-rump length at the first ultrasound screening between 10+ and 13 weeks was used to determine the gestational age. 2,962 blood samples from mothers and children were genotyped. After genotype quality control (QC) procedure and applying the phenotype-based inclusion/exclusion criteria, 1,170 mother/child pairs were selected and used in the analysis. The study was approved by the Ethics Committee of Oulu University Hospital and that of Helsinki University Central Hospital. Written informed consent was given by all participants.

MoBa

The Mother Child dataset of Norway (MoBa) is a nationwide Norwegian pregnancy study administered by the Norwegian Institute of Public Health. The study includes more than 114,000 children, 95,000 mothers and 75,000 fathers recruited from 1999 through 2008 [2]. Gestational age was estimated by ultrasound at gestational weeks 17–19. In the few cases without ultrasound dating, gestational age was estimated using the date of the last menstrual period. For the current study, we used the mother-child pairs that were selected from Version 4 of the MoBa dataset, which included a total of 71,669 pregnancies [3]. Singleton live-born spontaneous pregnancies with mothers in the age group 20–34 years were selected. Random sampling was done from two gestational age ranges 154-258 days (cases) and 273–286 days (controls). Pregnancies involving pre-existing medical conditions, pregnancies with complications as well as pregnancies conceived by in vitro fertilization, were excluded from the study. In total, blood samples from 3,120 mothers and children were genotyped [3]. 1,009 mother/child pairs that passed QC and inclusion/exclusion criteria were included in the analysis. All parents gave informed, written consent. The study was approved by The Regional Committee for Medical Research Ethics in South-Eastern, Norway.

DNBC

The Danish National Birth Cohort (DNBC) followed over 100,000 pregnancies between 1996 and 2003 with extensive epidemiologic data on health outcomes in both mother and child [4]. The current study used the data downloaded from the Database of Genotypes and Phenotypes (dbGaP) (phs000103.v1.p1), which contains data from a genome-wide case/control study using approximately 1,000 preterm mother-child pairs (gestational age between 22–37 weeks) from the DNBC, along with 1,000 control pairs in which the child was born at ~40 weeks gestation. Gestational duration in this dataset was determined by a consensus algorithm combining all available information from multiple sources: self-reported date of last menstrual period, self-reported delivery date, and gestational age at birth registered in the Medical Birth Register and the National Patient Register. Of the 3,886 samples with genotype data, we identified 1,739 mother/child pairs and used them in the analysis. The study protocol was approved by the Danish Scientific Ethical Committee and the Danish Data Protection Agency.

HAPO

The Hyperglycemia and Adverse Pregnancy Outcome (HAPO) Study [5] is a multicenter, international study in which high quality phenotypic data related to fetal growth and maternal glucose metabolism has been collected from 25,000 pregnant women of varied racial and socio-demographic backgrounds using standardized protocols that were uniform across centers. For the current study, we utilized phenotype and genotype data of 1,500 infants and their mothers of European descent download from dbGaP (phs000096.v2.p1). The samples were collected from Toronto, Canada, Belfast, UK, Brisbane and Newcastle, Australia. Gestational duration in this dataset was determined by last menstrual period or ultrasound estimation from 6-24 weeks. Of the 2,866 samples with genotype data, we identified 1,089 mother/child pairs and used them in the analysis.

GPN

The Genomic and Proteomic Network for Preterm Birth Research (GPN) study [6] is a multicenter observational genome-wide association study (GWAS) designed to determine the genetic predisposition to idiopathic preterm birth. Phenotype data and genotype data from 743 spontaneous preterm births (20 to less than 34 weeks gestation), and 752 controls (39 to less than 42 weeks gestation) of diverse ethnic background (White, Hispanics, African Americans, and Others) were collected. For this current study, we identified 343 mother/child pairs of European descent from the data downloaded from dbGaP (phs000714.v1.p1).

ALSPAC

The Avon Longitudinal Study of Parents and Children (ALSPAC) is a prospective birth cohort study. 14,541 pregnant women resident in the former county of Avon (situated around the city of Bristol in the South West of England) with expected dates of delivery 1st April 1991 to 31st December 1992 were recruited [7, 8]. The children arising from these women, and their partners were followed up intensively over nearly three decades. Genotype data of the mothers and children were generated using the Illumina HumanHap550 quad (children) and Illumina human660W quad (mothers). This resulted in a dataset of 17,842 participants (either mothers or offspring), containing 6,305 mother-offspring pairs, each with 465,740 SNPs genotyped. From this data set, 5,384 mother-offspring pairs who passed genotype QC and inclusion/exclusion criteria were included in the analysis. Please note that the study website contains details of all the data that is available through a fully searchable data dictionary and variable search tool (http://www.bristol.ac.uk/alspac/researchers/our-data/).

Phenotype data

Phenotype measures

In this study, we focused on maternal height, pregnancy BMI, blood pressure and blood glucose levels measured during pregnancy and pregnancy outcomes including gestational duration (and preterm birth as a binary trait), birth weight and birth length. Maternal height, weight and most of the birth outcomes were available in most of the data sets (except for birth weight and length were not available from MoBa and birth length was not available in the DNBC data set).

Maternal blood pressure data was available in DNBC and ALSPAC. In the HAPO study, blood pressure was measured once between 24 and 32 (mean 28) weeks when the mothers underwent a 75-g oral glucose tolerance test. In the ALSPAC study, all blood pressure measurements taken during antenatal care were extracted from medical records with each woman having a median (interquartile range) of 13 (11, 16) measurements between 8 weeks and delivery [9]. We used average of the blood pressure measured between 30 and 36 weeks to match the gestational age when the blood pressure was measured in HAPO. The individual measurements were not available to this study.

In the HAPO study, fasting plasma glucose (FPG) levels in the mothers were measured between 24 and 32 weeks gestation as part of the standard oral glucose tolerance test. In the ALSPAC study, FPG was measured in some mothers in a follow-up data collection 18 years after the pregnancy.

Inclusion/exclusion criteria

We studied singleton pregnancies with spontaneous live births – including spontaneous deliveries with or without premature rupture of membranes (PROM). C-sections after spontaneous onset of labor were retained. Medically indicated deliveries or C-sections and repeat C-sections were excluded. Mother/child pairs without gestational duration information were excluded. Pregnancies with known gestational or fetal complications (e.g. placental abnormities, chorioamnionitis, preeclampsia, and congenital anomalies) and pregnancies involving pre-existing medical conditions (i.e. hypertension or diabetes) or maternal risk exposure (e.g. drug use during pregnancy) known be associated with preterm birth were also excluded. Pregnancies with gestational diabetes and gestational hypertension were not excluded for the investigation of maternal physiological changes during pregnancy.

Genotype data

The genomewide SNP data of the data sets were generated using either Affymetrix 6.0 or various Illumina arrays. After genotype calling, similar genotype QC procedures were applied. An overview of the data procedure was shown in S1 Fig.

Genotype calls

For the FIN dataset, genotyping was conducted using Affymetrix 6.0 (Affymetrix, California, United States) and various other Illumina arrays (Illumina, California, United States). For the Affymetrix SNP Array 6.0, genotype calls were determined using the CRLMM algorithm [10, 11] among chips that passed the vendor-suggested QC (Contrast QC > 0.4). For the Illumina chips, the genotype calling was conducted using Illumina's genotyping module v1.94 in the GenomeStudio v2011.1.

The samples from the MoBa dataset were genotyped using the Illumina Human660W-Quadv1_A bead chip (Illumina Inc.) and the genotype calls were determined using CRLMM algorithm.

The DNBC samples were genotyped using Human660W-Quad bead arrays from Illumina. The raw genotype intensity data (*.idat) files were obtained from dbGaP and we performed genotype calls using CRLMM algorithm.

The HAPO samples of European descent were genotyped using Human610-Quad array. We obtained the raw genotype intensity data (*.idat) files from dbGaP and performed genotype calls using CRLMM algorithm.

The processed genotype calls in plink format of the GPN data set was obtained from dbGaP (phs000714.v1.p1). Data from participants of apparent duplications with others (IBD>0.8), with sex discrepancies (between known sex and genetically inferred sex), and children with high Mendelian errors (>10%) were deleted.

The ALSPAC genotype data were generated using the Illumina HumanHap550 quad (children) and Illumina human660W quad (mothers). The cleaned genotype calls of 465,740 SNPs of 17,842 individuals were obtained from ALSPAC.

Genotype QC procedures

We performed similar genotype QC across of all the dataset (S1 Fig). We first performed sample-level QC based on call rate, overall heterogeneity, sex discrepancies; we checked the pedigree relationship based IBD analysis; Genetic ancestry was assessed by principal components analysis (PCA) anchored by 1000 Genomes reference samples. Individuals with non-European ancestry were excluded. We then perform marker level QC: SNPs with low call rate (<98%), low minor allele frequency (<0.01) or significant deviation from Hardy-Weinberg Equilibrium ($p < 5\times10^{-6}$) were excluded.

Genomewide phasing, imputation and inference of allelic transmission

The genotype data of the mothers and infants was phased together using Shapeit2 software [12] and then the estimated haplotypes were used to impute untyped SNPs using the reference haplotypes extracted from the Phase III 1000 Genomes Project [13] by Minimac3 [14]. The genotype data of the FIN data set was generated using multiple platforms. The pre-phasing and imputation were done separately by each platform. To fully utilize the mother/child pair relationships across all the sample, we merged the imputed data across all the samples and conducted re-phasing using Shapeit2 (S1 Fig).

The haplotype phasing information in mother/infant pairs was used to infer allele transmission between mothers and their infants. When phasing in mother/infant duos, the Shapeit2 automatically accommodates the mother/child relationship and imposes constraints on each individual's haplotypes based on allele transmission between a mother and her child [12]. We did not use the duoHMM argument (for estimating haplotypes in general pedigrees [15]) when running the Shapeit2 as we only included mother/infant duos from the data sets and excluded other close related samples. Without invoking the duoHMM argument, the first phased haplotype of a mother/child pair reported by Shapeit2 is the transmitted haplotype.

Genetic scores

Selection of GWA SNPs

Reported GWA SNPs robustly associated with body height, BMI, blood pressure, fasting plasma glucose (FPG) levels and type 2 diabetes susceptibility (T2D) were used to construct weighted genetic scores to instrument various maternal phenotypes (S2 Table). In addition, we also constructed genetic scores for birth weight.

Height and BMI: The GWA SNPs associated with height and BMI reported by the 2018 GIANT and UK BioBank Meta Analysis [16] were download from the GIANT website (https://portals.broadinstitute.org/collaboration/giant/). From the top 3,290 height SNPs based on conditional & joint COJO analysis, we selected 2,390 SNPs with primary *p*-value < 1E-8 and among these 2,382 SNPs were diallelic and polymorphic in the 1000 Genomes European reference samples. Further, we excluded 141 SNPs because they were in substantial LD (r^2 > 0.2) with other more significant SNPs. As a result, 2,214 SNPs and their reported beta values were used to construct height genetic scores. Following a similar procedure, we selected 643 SNPs from the reported top 941 BMI SNPs to construct BMI genetic scores.

Blood pressure: For blood pressure, we extracted the 885 SNPs and their estimated effect sizes from the most recent GWAS [17] (Supplementary Table 24 of the original report). Among this list 5 SNPs were not polymorphic in the 1000 Genomes European reference samples, one SNP had zero effect and one was in close LD with another more significant SNP, were excluded. The final list included 878 SNPs. These SNPs together with their estimated effects on systolic (SBP), diastolic blood pressure (DBP) were used to build genetic scores for SBP and DBP, respectively. For simplicity, we also built a score for average blood pressure (BP) using the mean estimated effects of SBP and DBP.

Blood glucose and T2D susceptibility: 23 GWA SNPs associated with fasting glucose levels (FPG) identified from 96,496 individuals without diabetes [18] and their estimated effect sizes accounted for BMI were used to construct genetic scores for blood glucose levels. In addition, we also constructed genetic scores for susceptibility to Type 2 diabetes (T2D) using the SNPs reported by a recent GWA study in 898,130 European-descent [19]. Among the 403 reported SNPs (Supplementary Table 4 of the original report), 21 SNPs were excluded either due to non-polymorphic or LD redundancy. To contrast with the genetic regulation of normal glucose levels, we also excluded 13 SNPs which were in close LD with any of those 23 GWA SNPs associated

with normal fasting glucose levels. The final list of 369 SNPs and the log transformed BMI adjusted odds ratios (ORs) were used to construct T2D genetic scores.

Birth weight: We selected GWA SNPs associated with birth weight through fetal genetic effect from a recent GWA study using a large number of European samples [20]. This study used a structural equation modelling (SEM) method [21] to separate maternal from fetal effects. From the total 209 genome-wide significant SNPs (Supplementary Table 6 of the original report), we used 106 SNPs with confirmed fetal genetic effects (SEM classification: "Fetal Only" or "Fetal and Maternal"). After removing 10 SNPs due to LD redundancy or co-locating with other SNP, 96 SNPs and their fetal effects estimated by the SEM method were used to construct birth weight genetic score and to model fetal genetic effect on fetal growth.

Construction of genetic scores

The weighted genetic score was constructed as $S = \sum b_i G_i$, where $G_i = 0, 1 \text{ or } 2$ (for genotype) or $G_i = 0 \text{ or } 1$ (for haplotype genetic score), which indicates the number of reference alleles for a specific SNP, and b_i is the estimated allelic effect reported by the GWA studies. For a set of GWA SNPs associated with a particular maternal (adult) phenotype, we constructed two genotype genetic scores: S_{mat} (maternal genotype score), S_{fet} (fetal genotype score); and three haplotype genetic scores: S_{h1}, S_{h2} and S_{h3} respectively based on the maternal transmitted (h1), maternal non-transmitted (h2) and paternal transmitted alleles (h3) (Fig 2). Obviously, the maternal genotype score is the summation of the two maternal (transmitted and non-transmitted) haplotype scores ($S_{\text{mat}} = S_{\text{h1}} + S_{\text{h2}}$); the fetal genotype score is the summation of the two transmitted haplotype scores ($S_{\text{fet}} = S_{\text{h1}} + S_{\text{h3}}$).

Statistical analyses

Phenotypic associations

We first assessed statistical associations between the maternal phenotypes (X) and the pregnancy outcomes (Y) using regression models with a common form: $Y \sim X + \text{Cov}$, where Cov is a list of appropriate covariates. Specifically, the regression models are:

- $\text{gday} \sim \text{age1} + \text{age2} + \text{ht} + \text{bmi} + \text{sex}$
- $\text{pre} \sim \text{age1} + \text{age2} + \text{ht} + \text{bmi} + \text{sex}$
- $\text{bwt} \sim \text{age1} + \text{age2} + \text{ht} + \text{bmi} + \text{sex} + \text{gday1} + \text{gday2} + \text{gday3}$
- $\text{blen} \sim \text{age1} + \text{age2} + \text{ht} + \text{bmi} + \text{sex} + \text{gday1} + \text{gday2} + \text{gday3}$

In the above models, **gday** is the gestational duration measured in days, **pre** is the binary preterm birth status, **sex** is the fetal sex, **ht** and **bmi** are maternal prepregnant height and BMI and **age1** and **age2** are the first two polynomials of maternal age to capture the nonlinear effect of maternal age on pregnancy outcomes. Since birth weight (**bwt**) and birth length (**blen**) were primarily defined by gestational duration (**gday**), the first three polynomials (**gday1**, **gday2** and **gday3**) of gestational duration were included in regression models for **bwt** and **blen** to account for the strong effects of gestational duration on these birth outcomes.

In the HAPO and ALSPAC data sets, we also tested the impacts of maternal blood fasting glucose levels (**fpg**) and blood pressures (**sbp** and **dbp**) on the pregnancy outcomes using similar regression models by adding these variables as additional independent variable in the above regression models.

Associations between genetic scores and maternal phenotypes

Associations between the various genetic scores and their corresponding maternal phenotypes were examined using regression analyses. In the analysis of maternal height (**ht**) and maternal pre-pregnancy BMI (**bmi**), the first two polynomials of maternal age (**age1** and **age2**) were included as covariates. In the analysis of maternal blood pressure measures (**sbp** and **dbp**) and maternal fasting glucose levels (**fpg**), maternal height (**ht**), maternal BMI (**bmi**) and fetal sex (**sex**) were also included as covariates, as these factors were significantly associated with the maternal blood pressure (**sbp** and **dbp**) and maternal fasting glucose levels (**fpg**) measured during pregnancy.

- $\mathrm{ht} \sim \mathrm{scores} + \mathrm{age1} + \mathrm{age2}$
- $\mathrm{bmi} \sim \mathrm{scores} + \mathrm{age1} + \mathrm{age2}$
- $\mathrm{bp} \sim \mathrm{scores} + \mathrm{age1} + \mathrm{age2} + \mathrm{height} + \mathrm{bmi} + \mathrm{sex}$
- $\mathrm{fpg} \sim \mathrm{scores} + \mathrm{age1} + \mathrm{age2} + \mathrm{height} + \mathrm{bmi} + \mathrm{sex}$

Associations between genetic scores and pregnancy outcomes

To investigate the maternal or fetal genetic effects of a genetic score on a birth outcome, we tested associations between the three haplotype genetic scores and the pregnancy outcomes using regression models like those used in the observational association analyses, except the maternal phenotype under consideration was replaced by the three haplotype genetic scores ($S_{\mathrm{h1}} + S_{\mathrm{h2}} + S_{\mathrm{h3}}$). Hence the regression models for haplotype genetic score analysis take the form: $Y \sim S_{\mathrm{h1}} + S_{\mathrm{h2}} + S_{\mathrm{h3}} + \mathrm{Cov}$. For example, the following regression models were used to examine the association between height genetic scores and birth outcomes:

- $\mathrm{gday} \sim S_{\mathrm{h1}} + S_{\mathrm{h2}} + S_{\mathrm{h3}} + \mathrm{age1} + \mathrm{age2} + \mathrm{bmi} + \mathrm{sex}$
- $\mathrm{pre} \sim S_{\mathrm{h1}} + S_{\mathrm{h2}} + S_{\mathrm{h3}} + \mathrm{age1} + \mathrm{age2} + \mathrm{bmi} + \mathrm{sex}$
- $\mathrm{bwt} \sim S_{\mathrm{h1}} + S_{\mathrm{h2}} + S_{\mathrm{h3}} + \mathrm{age1} + \mathrm{age2} + \mathrm{bmi} + \mathrm{sex} + \mathrm{gday1} + \mathrm{gday2} + \mathrm{gday3}$
- $\mathrm{blen} \sim S_{\mathrm{h1}} + S_{\mathrm{h2}} + S_{\mathrm{h3}} + \mathrm{age1} + \mathrm{age2} + \mathrm{bmi} + \mathrm{sex} + \mathrm{gday1} + \mathrm{gday2} + \mathrm{gday3}$

The associations between these haplotype-based scores (S_{h1}, S_{h2} and S_{h3}), i.e. the estimates of the regression coefficients ($\hat{\beta}_{\mathrm{h1}}$, $\hat{\beta}_{\mathrm{h2}}$, and $\hat{\beta}_{\mathrm{h3}}$) and their associated *p*-values can explicitly model maternal effect vs fetal genetic effect (Fig 2) [22, 23]. In particular, an association between S_{h2} (maternal non-transmitted haplotype score) with a pregnancy outcome is a clear sign of maternal effect; whereas an association with S_{h3} (paternal transmitted haplotype score) indicates direct fetal genetic effect on a pregnancy outcome.

Modeling of maternal and fetal genetic effects using linear hypotheses

In addition, we modeled the maternal effect and the fetal effect as the linear combinations of the regression coefficients (β_{h1}, β_{h2}, β_{h3}) of the three haplotype genetic scores (Fig 2). Under the assumptions of additivity between maternal and fetal effect and zero parent of origin effect, the effect (β_{h1}) of the maternal transmitted haplotype (h1) should equal to the summation of the maternal effect (β_{h2}) of the non-transmitted haplotype (h2) and fetal genetic effect (β_{h3}) of the paternal transmitted haplotype (h3). Thus, ($\beta_{\mathrm{h1}} - \beta_{\mathrm{h3}}$) and ($\beta_{\mathrm{h1}} - \beta_{\mathrm{h2}}$) respectively represent the maternal effect and the fetal genetic effect of the maternal transmitted haplotype (h1). Therefore, we can model the maternal and fetal genetic effect on a pregnancy outcome as:

- <u>Maternal effect</u>: $\beta_{MY} = (\beta_{\mathrm{h1}} + \beta_{\mathrm{h2}} - \beta_{\mathrm{h3}})/2$ models the joint effect of the two maternal haplotypes ($\beta_{\mathrm{h1}} + \beta_{\mathrm{h2}}$) and excludes the possible fetal effect of the transmitted haplotype h1 by subtracting β_{h3}.
- <u>Fetal genetic effect</u>: $\beta_{FY} = (\beta_{\mathrm{h1}} - \beta_{\mathrm{h2}} + \beta_{\mathrm{h3}})/2$ models the joint effect of the maternal and the paternal transmitted haplotypes ($\beta_{\mathrm{h1}} + \beta_{\mathrm{h3}}$) and excludes the possible maternal effect of the transmitted haplotype h1 by subtracting β_{h2}.

Modeling of maternal and fetal genetic effect using the linear combinations of the regression coefficients of the three haplotype genetic scores are more powerful (hence smaller standard error) than a single haplotype score (e.g. S_{h2} for maternal effect and S_{h3} for fetal effect) when the assumptions of additivity between maternal and fetal effect and zero parent of origin effect ($\beta_{\mathrm{h1}} = \beta_{\mathrm{h2}} + \beta_{\mathrm{h3}}$) hold.

Estimation of maternal causal effects

The effect size estimates of the maternal non-transmitted haplotype score ($\hat{\beta}_{\mathrm{h2}}$) or the linear combination ($\hat{\beta}_{MY} = (\beta_{\mathrm{h1}} + \widehat{\beta_{\mathrm{h2}}} - \beta_{\mathrm{h3}})/2$) for maternal effect can be interpreted as a two-sample Mendelian randomization estimates of maternal causal effect. The first estimate ($\hat{\beta}_{\mathrm{h2}}$) based on the maternal non-transmitted alleles (h2), exclusively instruments the maternal effect. The second estimate ($\hat{\beta}_{MY}$) based on the linear combination of the regression coefficients of the three haplotype scores, although less specific (because it includes the transmitted alleles and needs the assumptions of additivity and zero parent of origin effect), is more powerful because it also considers the maternal effect of the transmitted alleles in instrumenting the maternal effect. These estimates evaluate the change in a pregnancy outcome caused by *certain amount* of change in a maternal phenotype caused by one unit change in the genetic score (which can cause one unit change in the adult phenotype).

For a maternal phenotype (e.g. maternal pre-pregnancy height), the weighted genetic score was constructed using the GWA SNPs and their estimated effect sizes of a “same” adult phenotype (e.g. adult height). In the reference study, one unit genetic score should associated exactly with one unit difference in the adult phenotype; however, it may not associated with the same amount of difference in the corresponding maternal phenotype because of the heterogeneity due to different sex, age, etc from the reference study. For example, one unit (mmHg) SBP and DBP genetic score only associated with 0.27mmHg and 0.32mmHg changes in maternal SBP and DBP in this study (S4 Table). Therefore, the effect size estimate ($\hat{\beta}_{\mathrm{h2}}$ or $\hat{\beta}_{MY}$) needs to be adjusted by their effect on the maternal phenotype (β_{MX}) to reflect the real magnitude of the causal effect (i.e. amount of change in a pregnancy outcome caused by one unit change in the maternal phenotype). We estimated the maternal causal effect by the ratio estimate [24]:

$$\hat{\beta}_{XY}^{m} = \hat{\beta}_{MY}/\hat{\beta}_{MX}$$

The standard error can be obtained based on the asymptotic normal approximation [25]:

$$\mathrm{se}(\hat{\beta}_{XY}^{m}) = \sqrt{\frac{\mathrm{se}(\hat{\beta}_{MY})^2}{\hat{\beta}_{MX}^{\;2}} + \frac{\hat{\beta}_{MY}^{\;2}\,\mathrm{se}(\hat{\beta}_{MX})^2}{\hat{\beta}_{MX}^{\;4}}}$$

As an alternative, we also utilized the maternal non-transmitted haplotype score (S_{h2}) as genetic instrument [22] and estimated the maternal causal effects using the two-stage least squares analysis (TSLS) [24].

Estimation of genetically confounded associations due to shared genetic effects

The effect size estimates of the paternal transmitted haplotype score ($\hat{\beta}_{\mathrm{h3}}$) or the linear combination ($\hat{\beta}_{FY} = (\beta_{\mathrm{h1}} - \widehat{\beta_{\mathrm{h2}}} + \beta_{\mathrm{h3}})/2$) for fetal genetic effect reflect the genetic variants associated with an adult phenotype have (shared) genetic effect on a pregnancy outcome. Quantitatively, they describe the amount of change in a pregnancy outcome caused by the genetic alleles that are associated with one unit difference in an adult phenotype. This shared genetic effect of the alleles associated with an adult phenotype X' (or the corresponding maternal phenotype X) on a pregnancy outcome (Y) can confound the association between a maternal phenotype (X) and a pregnancy outcome (Y) as well as the association between the pregnancy outcome (Y) and the adult phenotype (X') in offspring (Fig 1 and 2). In the following, we provided the mathematics for estimating these confounding associations from the estimated shared genetic effect ($\hat{\beta}_{FY}$).

Considering a general scenario (S2 Fig), in which two random variables (X and Y) are influenced by a confounder Z. The correlation between X and Y due to confounding is:

$$\rho'_{XY} = \rho_{ZX}\rho_{ZY} = \rho_{ZX}^2 \rho_{ZY}/\rho_{ZX}$$

If expressing the correlations using regression coefficients following ($\rho_{XY} = \beta_{XY}\frac{\mathrm{sd}(X)}{\mathrm{sd}(Y)}$), the formula can be written as:

$$\beta'_{XY}\frac{\mathrm{sd}(X)}{\mathrm{sd}(Y)} = \rho_{ZX}^2 \left(\beta_{ZY}\frac{\mathrm{sd}(Z)}{\mathrm{sd}(Y)}\right)\Big/\left(\beta_{ZX}\frac{\mathrm{sd}(Z)}{\mathrm{sd}(X)}\right)$$

which gives:

$$\beta'_{XY} = \rho_{ZX}^2 \frac{\beta_{ZY}}{\beta_{ZX}} \text{ or } \beta'_{XY} = \rho_{ZY}^2 \frac{\mathrm{Var}(Y)}{\mathrm{Var}(X)}\frac{\beta_{ZX}}{\beta_{ZY}}$$

By applying this general conclusion to the two confounding scenarios due to shared genetic effect (S3 Fig), we can estimate the magnitudes of the genetically confounded associations.

Scenario 1: Genetically confounded association (β_{XY}^c) between a maternal phenotype (X) and a pregnancy outcome (Y) (S3 Fig, A). In this case, the confounder is set of the transmitted alleles (Z) that are associated with a maternal phenotype (X) and the genetically confounded association can be written as:

$$\beta_{XY}^c = \frac{h_X^2}{2}\frac{\beta_{FY}}{\beta_{MX}}$$

where ρ_{ZX}^2 is replaced by the half of the heritability of X (h_X^2) and β_{MX} and β_{FY} are respectively the regression coefficient of genetic effects on a maternal phenotype (X) and a pregnancy outcome (Y). Assuming all the genetic variants (including those unidentified ones) associated with a maternal phenotype (X) have similar effects as those estimated from the genetic score built on the known GWA SNPs ($\hat{\beta}_{MX}$ and $\hat{\beta}_{FY}$ for maternal and fetal genetic effects), then we can estimate the magnitude of the genetically confounded association by:

$$\hat{\beta}_{XY}^c = \frac{h_X^2}{2}\frac{\hat{\beta}_{FY}}{\hat{\beta}_{MX}}$$

As the ratio of two normally distributed variables (i.e. $\hat{\beta}_{FY}$ and $\hat{\beta}_{MX}$), there is no simple analytical solution to obtain the distribution of this estimate ($\hat{\beta}_{XY}^c$). We calculated the standard error (se) using a normal approximation (the formula is similar to the ratio estimate above):

$$\mathrm{se}(\hat{\beta}_{XY}^c) = \frac{h_X^2}{2}\sqrt{\frac{\mathrm{se}(\hat{\beta}_{FY})^2}{\hat{\beta}_{MX}{}^2} + \frac{\hat{\beta}_{FY}{}^2\,\mathrm{se}(\hat{\beta}_{MX})^2}{\hat{\beta}_{MX}{}^4}}$$

The heritability of a maternal phenotypes (X) is not always same as the corresponding adult phenotype. We therefore calculated the heritability of a maternal phenotype (h_X^2) from the heritability of the corresponding adult phenotype (h^2, S2 Table) by:

$$h_X^2 = h^2\frac{R_{\mathrm{mat}}^2}{R_{\mathrm{rep}}^2}$$

where R_{rep}^2 and R_{mat}^2 are respectively the variance explained by the same genetic score in the adult phenotype (as reported by the reference GWA study).and in a maternal phenotype (as observed in this current study). The intuition behind this calculation is that we assume a same set of genetic variants influence both the adult phenotype and the maternal phenotype and their cumulative additive genetic effects on these traits are proportional to the cumulative additive genetic effects of the GWA SNPs (variance explained by the genetic score).

Scenario 2: Genetically confounded association ($\beta_{YX'}^c$) between a pregnancy outcome (Y) and an adult (late) phenotype (X') in offspring (S3 Fig, B). In this case, the confounder includes the maternal as well as the paternal transmitted alleles that are associated with adult phenotype (X'). The maternal transmitted alleles can be associated with the pregnancy outcome (Y) by both maternal effect (β_{MY}) and fetal genetic effect (β_{FY}). The genetically confounded association between a pregnancy outcome (Y) and adult phenotype (X') is given by:

$$\beta^{c}_{YX'} = h^2 \frac{\mathrm{Var}(X')}{\mathrm{Var}(Y)} \left(\frac{\beta_{MY}}{2} + \beta_{FY}\right) / \beta_{FX'} \quad \text{(Method 1)}$$

Where h^2 is the heritability of the adult phenotype (X'), β_{MY} and β_{FY} are the regression coefficients of maternal effect and fetal genetic effect on a pregnancy outcome (Y) and $\beta_{FX'}$ is the regression coefficient of genetic effect on an adult phenotype (X') in offspring. This estimate partitions the genetically confounded association into the maternal ($\frac{\beta_{MY}}{2}$) and the fetal component (β_{FY}).

Considering that the maternal non-transmitted alleles (h2) has no effect on the confounding, we can also use a simple alternative to estimate the genetically confounded association:

$$\beta^{c}_{YX'} = h^2 \frac{\mathrm{Var}(X')}{\mathrm{Var}(Y)} \left(\frac{\beta_{\mathrm{h1}} + \beta_{\mathrm{h3}}}{2}\right) / \beta_{FX'} \quad \text{(Method 2)}$$

If the assumptions of additivity and zero parent of origin effect ($\beta_{\mathrm{h1}} = \beta_{\mathrm{h2}} + \beta_{\mathrm{h3}}$) hold, this formula is mathematically equivalent to the previous one. This simple formula uses the combination of two regression coefficients ($\beta_{\mathrm{h1}} + \beta_{\mathrm{h3}}$) and does not require the assumption of zero parent of origin effect. It is theoretically more accurate and precise than the previous one in statistical inference; however, it cannot dissect the maternal and fetal contributions of the confounding.

Although we did not directly estimate $\beta_{FX'}$ (because the adult phenotype is not available), the magnitude of the association should equal to one ($\beta_{FX'} = 1$) if the reference GWA study (from which we extracted the GWA SNPs and effect size estimates to construct the genetic scores) is representative of (or homogenous with) our study population. Again, if assuming all the genetic variants associated with the adult phenotype (X') have similar maternal and fetal genetic effects as those estimated from the genetic score built on known GWA SNPs, then we can estimate the genetically confounded association between a pregnancy outcome (Y) and an adult (late) phenotype (X') in offspring by:

$$\hat{\beta}^{c}_{YX'} = h^2 \frac{\mathrm{Var}(X')}{\mathrm{Var}(Y)} \left(\frac{\hat{\beta}_{MY}}{2} + \hat{\beta}_{FY}\right) \quad \text{(Method 1)}$$

or

$$\hat{\beta}^{c}_{YX'} = h^2 \frac{\mathrm{Var}(X')}{\mathrm{Var}(Y)} \left(\frac{\widehat{\beta_{\mathrm{h1}} + \beta_{\mathrm{h3}}}}{2}\right) \quad \text{(Method 2)}$$

By assuming the variance of the adult phenotype ($\mathrm{Var}(X')$) and its heritability (h^2), and the variance of the pregnancy outcome ($\mathrm{Var}(Y)$) as known population parameters, the estimate ($\hat{\beta}^{c}_{YX'}$) follows a normal distribution with mean ($\frac{\hat{\beta}_{MY}}{2} + \hat{\beta}_{FY}$ or $\frac{\widehat{\beta_{\mathrm{h1}}+\beta_{\mathrm{h3}}}}{2}$) and se scaled by $h^2 \frac{\mathrm{Var}(X')}{\mathrm{Var}(Y)}$.

We compared our estimated genetically confounded association ($\hat{\beta}^{c}_{YX'}$) between a pregnancy outcome (Y) and an adult (late) phenotype (X') (referred as the **confounded (eff)**) with:

1) The **confounded (cor)** phenotypic associations between birth weight and adult phenotypes based on the genetic correlations reported by Horikoshi et al. [26]. We estimated the phenotypic correlation using $R_{\mathrm{p}} = R_{\mathrm{g}}\sqrt{h_1^2 h_2^2}$, where R_{g} is the reported genetic correlation and $\sqrt{h_1^2 h_2^2}$ is the square root of the product of the heritability of an adult phenotype (h_1^2) and birth weight (h_2^2) (also see S2 Table).
2) The **observed** phenotypic associations from the ALSPAC data set. We calculated the regression coefficients between birth weight and offspring height, BMI, BP and FPG measured at age 17 in a subset of ALPAC samples. Gestational age and sex were included as covariates.
3) The **reported** associations between birth weight and BP and T2D susceptibility from a recent epidemiological meta study [27]. We extracted the “unstratified risk” of T2D and changes in SBP and DBP per 1kg increase in birth weight from Table 1 of the reference [27] and converted the values to changes in standard deviation (of an adult phenotype) per 1 SD (484g) increase in birth weight.

Multivariable Mendelian Randomization analysis

The analyses of associations between the haplotype genetic scores and birth outcomes provided a powerful way to illustrate the different maternal and fetal effects of the SNPs known to be associated with an intermediate phenotype. However, the genetic scores built on hundreds or thousands of SNPs were less specific, as they are more likely to be associated with other phenotypic traits, therefore introducing ambiguities in the interpretation of genetic score associations [28]. To circumvent this issue, we performed multivariable Mendelian Randomization (MR) analysis using the recently developed Mendelian randomization pleiotropy residual sum and outlier (MR-PRESSO) approach [29]. This method can detect and correct for SNPs with horizontal pleiotropic effects (outliers) in multivariable MR test. Using this approach, we tested whether the effects of the maternal transmitted (h1), maternal non-transmitted (h2) and paternal transmitted (h3) alleles of the GWA SNPs on a pregnancy outcome are

proportional to their reported effects on an intermediate phenotype by large GWA studies. The allele-specific effect estimates (for the h1, h2 and h3 alleles) of a SNP were obtained using the same regression methods as those in testing the associations between haplotype genetic scores and pregnancy outcomes ($Y \sim S_{\mathrm{h1}} + S_{\mathrm{h2}} + S_{\mathrm{h3}} + \mathrm{Cov}$), except for the haplotype genetic scores were replace by the haplotypes of a SNP.

In two-sample MR analysis [30], the non-zero slope of the regression line between the reported effect on an exposure and the estimated effect on an outcome has usually been interpreted as *causal effect*. In the current context, the allele-specific effects by the maternal transmitted (h1), maternal non-transmitted (h2) and paternal transmitted (h3) alleles on a pregnancy outcome were used to index the maternal effect (h2), fetal genetic effect (h3), or combination of both (h1). In this case, a non-zero "casual estimate"" (i.e. slope of the regression line between the reported effects of the SNPs on a maternal phenotype and the estimated allele-specific effect on a birth outcome) may or may not imply causal effect (S4 Fig). Using the height GWA SNPs as an example, the "causal estimate" of maternal height on gestational duration by the maternal non-transmitted alleles (h2) represents a maternal effect, and it is very likely that the causal path is mediated by maternal height. However, the "estimated effect" on birth weight by the paternal transmitted alleles (h3) indicates a fetal genetic effect and the estimated effect sizes on birth weight are even proportional to their effects on adult height, which most probably reflects that the pleiotropic effects of the height associated SNPs on both birth weight and adult height act though a common pathway [31]. A significant MR-PRESSO global test indicates that the allele-specific effect sizes of the variants on an outcome are not always proportional to their effects on an intermediate phenotype. In cases of causal inferences (e.g. utilizing non-transmitted alleles to infer causal effect of a maternal trait on a birth outcome, S4 Fig, A), this is a sign of horizontal pleiotropy – meaning the identified maternal effect may not exclusively be mediated by the intermediate maternal phenotype. In cases of genetic correlation between a birth outcome and an adult phenotype (e.g. the fetal effects of height associated SNPs on birth weight, S4 Fig, B), this suggests the genetic effects of the SNPs on a birth outcome are not always proportional to their reported effects on the adult phenotype, which might suggest involvement of different molecular pathways.

Meta-analysis

Fixed-effect meta-analysis was used to combine the results from different studies. Between-study heterogeneity was tested by Cochran's Q test.

References

1. Plunkett J, Doniger S, Orabona G, Morgan T, Haataja R, Hallman M, et al. An evolutionary genomic approach to identify genes involved in human birth timing. PLoS Genet. 2011;7(4):e1001365. doi: 10.1371/journal.pgen.1001365. PubMed PMID: 21533219; PubMed Central PMCID: PMC3077368.

2. Magnus P, Birke C, Vejrup K, Haugan A, Alsaker E, Daltveit AK, et al. Cohort Profile Update: The Norwegian Mother and Child Cohort Study (MoBa). Int J Epidemiol. 2016;45(2):382-8. doi: 10.1093/ije/dyw029. PubMed PMID: 27063603.

3. Myking S, Boyd HA, Myhre R, Feenstra B, Jugessur A, Devold Pay AS, et al. X-chromosomal maternal and fetal SNPs and the risk of spontaneous preterm delivery in a Danish/Norwegian genome-wide association study. PLoS One. 2013;8(4):e61781. doi: 10.1371/journal.pone.0061781. PubMed PMID: 23613933; PubMed Central PMCID: PMC3628886.

4. Olsen J, Melbye M, Olsen SF, Sorensen TI, Aaby P, Andersen AM, et al. The Danish National Birth Cohort--its background, structure and aim. Scand J Public Health. 2001;29(4):300-7. PubMed PMID: 11775787.

5. HAPO Study Cooperative Research Group, Metzger BE, Lowe LP, Dyer AR, Trimble ER, Chaovarindr U, et al. Hyperglycemia and adverse pregnancy outcomes. N Engl J Med. 2008;358(19):1991-2002. doi: 10.1056/NEJMoa0707943. PubMed PMID: 18463375.

6. Zhang H, Baldwin DA, Bukowski RK, Parry S, Xu Y, Song C, et al. A genome-wide association study of early spontaneous preterm delivery. Genet Epidemiol. 2015;39(3):217-26. doi: 10.1002/gepi.21887. PubMed PMID: 25599974; PubMed Central PMCID: PMCPMC4366311.

7. Boyd A, Golding J, Macleod J, Lawlor DA, Fraser A, Henderson J, et al. Cohort Profile: the 'children of the 90s'--the index offspring of the Avon Longitudinal Study of Parents and Children. Int J Epidemiol. 2013;42(1):111-27. Epub 2012/04/18. doi: 10.1093/ije/dys064. PubMed PMID: 22507743; PubMed Central PMCID: PMCPMC3600618.

8. Fraser A, Macdonald-Wallis C, Tilling K, Boyd A, Golding J, Davey Smith G, et al. Cohort Profile: the Avon Longitudinal Study of Parents and Children: ALSPAC mothers cohort. Int J Epidemiol. 2013;42(1):97-110. Epub 2012/04/18. doi: 10.1093/ije/dys066. PubMed PMID: 22507742; PubMed Central PMCID: PMCPMC3600619.

9. Macdonald-Wallis C, Tilling K, Fraser A, Nelson SM, Lawlor DA. Established preeclampsia risk factors are related to patterns of blood pressure change in normal term pregnancy: findings from the Avon Longitudinal Study of Parents and Children. J Hypertens. 2011;29(9):1703-11. Epub 2011/08/16. doi: 10.1097/HJH.0b013e328349eec6. PubMed PMID: 21841545.

10. Carvalho B, Bengtsson H, Speed TP, Irizarry RA. Exploration, normalization, and genotype calls of high-density oligonucleotide SNP array data. Biostatistics. 2007;8(2):485-99. doi: 10.1093/biostatistics/kxl042. PubMed PMID: 17189563.

11. Scharpf RB, Irizarry RA, Ritchie ME, Carvalho B, Ruczinski I. Using the R Package crlmm for Genotyping and Copy Number Estimation. J Stat Softw. 2011;40(12):1-32. PubMed PMID: 22523482; PubMed Central PMCID: PMC3329223.

12. Delaneau O, Marchini J, Zagury JF. A linear complexity phasing method for thousands of genomes. Nat Methods. 2012;9(2):179-81. doi: 10.1038/nmeth.1785. PubMed PMID: 22138821.

13. 1000 Genomes Project Consortium, Abecasis GR, Auton A, Brooks LD, DePristo MA, Durbin RM, et al. An integrated map of genetic variation from 1,092 human genomes. Nature. 2012;491(7422):56-65. doi: 10.1038/nature11632. PubMed PMID: 23128226; PubMed Central PMCID: PMC3498066.

14. Fuchsberger C, Abecasis GR, Hinds DA. minimac2: faster genotype imputation. Bioinformatics. 2015;31(5):782-4. doi: 10.1093/bioinformatics/btu704. PubMed PMID: 25338720.

15. O'Connell J, Gurdasani D, Delaneau O, Pirastu N, Ulivi S, Cocca M, et al. A general approach for haplotype phasing across the full spectrum of relatedness. PLoS Genet. 2014;10(4):e1004234. doi: 10.1371/journal.pgen.1004234. PubMed PMID: 24743097; PubMed Central PMCID: PMCPMC3990520.

16. Yengo L, Sidorenko J, Kemper KE, Zheng Z, Wood AR, Weedon MN, et al. Meta-analysis of genome-wide association studies for height and body mass index in approximately 700000 individuals of European ancestry. Hum Mol Genet. 2018;27(20):3641-9. Epub 2018/08/21. doi: 10.1093/hmg/ddy271. PubMed PMID: 30124842.

17. Evangelou E, Warren HR, Mosen-Ansorena D, Mifsud B, Pazoki R, Gao H, et al. Genetic analysis of over 1 million people identifies 535 new loci associated with blood pressure traits. Nat Genet. 2018;50(10):1412-25. Epub 2018/09/19. doi: 10.1038/s41588-018-0205-x. PubMed PMID: 30224653; PubMed Central PMCID: PMCPMC6284793.

18. Manning AK, Hivert MF, Scott RA, Grimsby JL, Bouatia-Naji N, Chen H, et al. A genome-wide approach accounting for body mass index identifies genetic variants influencing fasting glycemic traits and insulin resistance. Nat Genet. 2012;44(6):659-69. doi: 10.1038/ng.2274. PubMed PMID: 22581228; PubMed Central PMCID: PMCPMC3613127.

19. Mahajan A, Taliun D, Thurner M, Robertson NR, Torres JM, Rayner NW, et al. Fine-mapping type 2 diabetes loci to single-variant resolution using high-density imputation and islet-specific epigenome maps. Nat Genet. 2018;50(11):1505-13. Epub 2018/10/10. doi: 10.1038/s41588-018-0241-6. PubMed PMID: 30297969; PubMed Central PMCID: PMCPMC6287706.

20. Warrington NM, Beaumont RN, Horikoshi M, Day FR, Helgeland O, Laurin C, et al. Maternal and fetal genetic effects on birth weight and their relevance to cardio-metabolic risk factors. Nat Genet. 2019;51(5):804-14. Epub 2019/05/03. doi: 10.1038/s41588-019-0403-1. PubMed PMID: 31043758; PubMed Central PMCID: PMCPMC6522365.

21. Warrington NM, Freathy RM, Neale MC, Evans DM. Using structural equation modelling to jointly estimate maternal and fetal effects on birthweight in the UK Biobank. Int J Epidemiol. 2018;47(4):1229-41. Epub 2018/02/16. doi: 10.1093/ije/dyy015. PubMed PMID: 29447406; PubMed Central PMCID: PMCPMC6124616.

22. Zhang G, Bacelis J, Lengyel C, Teramo K, Hallman M, Helgeland O, et al. Assessing the Causal Relationship of Maternal Height on Birth Size and Gestational Age at Birth: A Mendelian Randomization Analysis. PLoS Med. 2015;12(8):e1001865. doi: 10.1371/journal.pmed.1001865. PubMed PMID: 26284790; PubMed Central PMCID: PMCPMC4540580.

23. Zhang G, Srivastava A, Bacelis J, Juodakis J, Jacobsson B, Muglia LJ. Genetic studies of gestational duration and preterm birth. Best Pract Res Clin Obstet Gynaecol. 2018;52:33-47. Epub 2018/07/17. doi: 10.1016/j.bpobgyn.2018.05.003. PubMed PMID: 30007778; PubMed Central PMCID: PMCPMC6290110.

24. Lawlor DA, Harbord RM, Sterne JA, Timpson N, Davey Smith G. Mendelian randomization: using genes as instruments for making causal inferences in epidemiology. Stat Med. 2008;27(8):1133-63. doi: 10.1002/sim.3034. PubMed PMID: 17886233.

25. Burgess S, Small DS, Thompson SG. A review of instrumental variable estimators for Mendelian randomization. Stat Methods Med Res. 2017;26(5):2333-55. Epub 2015/08/19. doi: 10.1177/0962280215597579. PubMed PMID: 26282889; PubMed Central PMCID: PMCPMC5642006.

26. Horikoshi M, Beaumont RN, Day FR, Warrington NM, Kooijman MN, Fernandez-Tajes J, et al. Genome-wide associations for birth weight and correlations with adult disease. Nature. 2016;538(7624):248-52. doi: 10.1038/nature19806. PubMed PMID: 27680694.

27. Knop MR, Geng TT, Gorny AW, Ding R, Li C, Ley SH, et al. Birth Weight and Risk of Type 2 Diabetes Mellitus, Cardiovascular Disease, and Hypertension in Adults: A Meta-Analysis of 7 646 267 Participants From 135 Studies. J Am Heart Assoc. 2018;7(23):e008870. Epub 2018/11/30. doi: 10.1161/JAHA.118.008870. PubMed PMID: 30486715.

28. Evans DM, Brion MJ, Paternoster L, Kemp JP, McMahon G, Munafo M, et al. Mining the human phenome using allelic scores that index biological intermediates. PLoS Genet. 2013;9(10):e1003919. doi: 10.1371/journal.pgen.1003919. PubMed PMID: 24204319; PubMed Central PMCID: PMC3814299.

29. Verbanck M, Chen CY, Neale B, Do R. Detection of widespread horizontal pleiotropy in causal relationships inferred from Mendelian randomization between complex traits and diseases. Nat Genet. 2018;50(5):693-8. Epub 2018/04/25. doi: 10.1038/s41588-018-0099-7. PubMed PMID: 29686387; PubMed Central PMCID: PMCPMC6083837.

30. Burgess S, Scott RA, Timpson NJ, Davey Smith G, Thompson SG, Consortium E-I. Using published data in Mendelian randomization: a blueprint for efficient identification of causal risk factors. Eur J Epidemiol. 2015;30(7):543-52. Epub 2015/03/17. doi: 10.1007/s10654-015-0011-z. PubMed PMID: 25773750; PubMed Central PMCID: PMCPMC4516908.

31. O'Connor LJ, Price AL. Distinguishing genetic correlation from causation across 52 diseases and complex traits. Nat Genet. 2018;50(12):1728-34. Epub 2018/10/31. doi: 10.1038/s41588-018-0255-0. PubMed PMID: 30374074.
