# Supplementary material for: Dissecting maternal and fetal genetic effects underlying the associations between maternal phenotypes, birth outcomes, and adult phenotypes: A mendelian-randomization and haplotype-based genetic score analysis in 10,734 mother–infant pairs
Source: PLoS Med. 2020 Aug 25;17(8):e1003305. doi: 10.1371/journal.pmed.1003305 (PMC7447062; doi:10.1371/journal.pmed.1003305)
Supplement: S1 Table — (PDF) [file pmed.1003305.s004.pdf]

S1 Table. Data sets and number of samples

Data set	Phenotype[a]	Genotype[b]		Filtered[c]			
	Record	Typed	Passed	Record	Mother	Infant	Duo
FIN	1644	2962	2710	1371	1322	1217	1170
MoBa	2035	3120	2979	1933	1804	1134	1009
DNBC	2068	3886	3799	2038	1912	1865	1739
HAPO	1507	2866	2743	1266	1203	1152	1089
GPN	1495	2852	1360	440	419	364	343
ALSPAC	15443	17842	17435	9806	7603	7587	5384
Total				16854	14263	13319	**10734**

a: Number of phenotype records (pregnancies)

b: Number of genotyped individuals (mothers and infants) and individuals passed genotype QC

c: Number of phenotype records (pregnancies), and number of mothers, infants and mother/infant duos after applying genotype QC and phenotype filtering. 10,734 was the total number of mother/infant duos used in the analyses.

Abbreviations: ALSPAC, The Avon Longitudinal Study of Parents and Children; DNBC, The Danish National Birth Cohort; FIN, The Finnish birth data set; GPN, The Genomic and Proteomic Network for Preterm Birth Research; HAPO, The Hyperglycemia and Adverse Pregnancy Outcome Study; MoBa, The Mother Child data set of Norway.
