# Supplementary material for: Dissecting maternal and fetal genetic effects underlying the associations between maternal phenotypes, birth outcomes, and adult phenotypes: A mendelian-randomization and haplotype-based genetic score analysis in 10,734 mother–infant pairs
Source: PLoS Med. 2020 Aug 25;17(8):e1003305. doi: 10.1371/journal.pmed.1003305 (PMC7447062; doi:10.1371/journal.pmed.1003305)
Supplement: S2 Table — GWA, genome-wide association; SNP, single-nucleotide polymorphism. (PDF) [file pmed.1003305.s005.pdf]

S2 Table. GWA SNPs used to build the genetic scores

Trait	Total[a]	Selected[b]	Used[c]	Unit[d]	Reference[e]	H^2 [Reference][f]
Height	3290	2214	2130	SD*	[1]	0.80 [2]
BMI	941	643	628	SD*	[1]	0.6 [3]
BP	885	878	831	mmHg	[4]	0.5 [5]
FPG	23	23	22	mmol/L	[6]	0.66 [7]
T2D	403	369	306	Log(OR)	[8]	0.45 [9]
Birth weight	106	96	86	SD*	[10]	0.3 [11]

a: Total number of GWA SNPs with genome-wide significance (p < 1E-8 (height, BMI) or p < 5E-8 (BP, FPG and T2D).

b: Number of selected GWA significant SNPs (polymorphic in 1000 Genomes European samples and not in close LD with other more significant SNPs).

c: SNPs that were available in all study data sets were used to constructing the genetic scores. The information of these SNPs can be found in Supplementary data (S1_Data.xlsx).

d: Units of the effect size estimates (SD: standard deviation) in the original GWA reports. Units using SD* were transformed to cm (height), kg/m^2 (BMI) and g (Birth weight) according 1-SD (height) = 6.4cm, 1-SD (BMI) = 4.0kg/m^2, and 1-SD (birth weight) = 484g in constructing the genetic scores.

e: Reference GWA studies

f: H^2: Reported heritability of the traits with source reference.

Abbreviations: BP, mean of the SBP (systolic blood pressure) and DBP (diastolic blood pressure) scores; BMI, body mass index; FPG, fasting plasma glucose; T2D, type 2 diabetes

References:

1. Yengo L, Sidorenko J, Kemper KE, Zheng Z, Wood AR, Weedon MN, et al. Meta-analysis of genome-wide association studies for height and body mass index in approximately 700000 individuals of European ancestry. Hum Mol Genet. 2018;27(20):3641-9. Epub 2018/08/21. doi: 10.1093/hmg/ddy271. PubMed PMID: 30124842.
2. Macgregor S, Cornes BK, Martin NG, Visscher PM. Bias, precision and heritability of self-reported and clinically measured height in Australian twins. Hum Genet. 2006;120(4):571-80. Epub 2006/08/26. doi: 10.1007/s00439-006-0240-z. PubMed PMID: 16933140.
3. Elks CE, den Hoed M, Zhao JH, Sharp SJ, Wareham NJ, Loos RJ, et al. Variability in the heritability of body mass index: a systematic review and meta-regression. Front Endocrinol (Lausanne). 2012;3:29. Epub 2012/05/31. doi: 10.3389/fendo.2012.00029. PubMed PMID: 22645519; PubMed Central PMCID: PMCPMC3355836.
4. Evangelou E, Warren HR, Mosen-Ansorena D, Mifsud B, Pazoki R, Gao H, et al. Genetic analysis of over 1 million people identifies 535 new loci associated with blood pressure traits. Nat Genet. 2018. doi: 10.1038/s41588-018-0205-x. PubMed PMID: 30224653.

5. Doris PA. The genetics of blood pressure and hypertension: the role of rare variation. Cardiovasc Ther. 2011;29(1):37-45. Epub 2010/12/07. doi: 10.1111/j.1755-5922.2010.00246.x. PubMed PMID: 21129164; PubMed Central PMCID: PMCPMC3562708.

6. Manning AK, Hivert MF, Scott RA, Grimsby JL, Bouatia-Naji N, Chen H, et al. A genome-wide approach accounting for body mass index identifies genetic variants influencing fasting glycemic traits and insulin resistance. Nat Genet. 2012;44(6):659-69. doi: 10.1038/ng.2274. PubMed PMID: 22581228; PubMed Central PMCID: PMCPMC3613127.

7. Simonis-Bik AM, Eekhoff EM, Diamant M, Boomsma DI, Heine RJ, Dekker JM, et al. The heritability of HbA1c and fasting blood glucose in different measurement settings. Twin Res Hum Genet. 2008;11(6):597-602. Epub 2008/11/20. doi: 10.1375/twin.11.6.597. PubMed PMID: 19016616.

8. Mahajan A, Taliun D, Thurner M, Robertson NR, Torres JM, Rayner NW, et al. Fine-mapping type 2 diabetes loci to single-variant resolution using high-density imputation and islet-specific epigenome maps. Nat Genet. 2018;50(11):1505-13. Epub 2018/10/10. doi: 10.1038/s41588-018-0241-6. PubMed PMID: 30297969; PubMed Central PMCID: PMCPMC6287706.

9. Fuchsberger C, Flannick J, Teslovich TM, Mahajan A, Agarwala V, Gaulton KJ, et al. The genetic architecture of type 2 diabetes. Nature. 2016;536(7614):41-7. doi: 10.1038/nature18642. PubMed PMID: 27398621; PubMed Central PMCID: PMCPMC5034897.

10. Warrington NM, Beaumont RN, Horikoshi M, Day FR, Helgeland O, Laurin C, et al. Maternal and fetal genetic effects on birth weight and their relevance to cardio-metabolic risk factors. Nat Genet. 2019;51(5):804-14. Epub 2019/05/03. doi: 10.1038/s41588-019-0403-1. PubMed PMID: 31043758; PubMed Central PMCID: PMCPMC6522365.

11. Lunde A, Melve KK, Gjessing HK, Skjaerven R, Irgens LM. Genetic and environmental influences on birth weight, birth length, head circumference, and gestational age by use of population-based parent-offspring data. Am J Epidemiol. 2007;165(7):734-41. doi: 10.1093/aje/kwk107. PubMed PMID: 17311798.
