# Supplementary material for: Dissecting maternal and fetal genetic effects underlying the associations between maternal phenotypes, birth outcomes, and adult phenotypes: A mendelian-randomization and haplotype-based genetic score analysis in 10,734 mother–infant pairs
Source: PLoS Med. 2020 Aug 25;17(8):e1003305. doi: 10.1371/journal.pmed.1003305 (PMC7447062; doi:10.1371/journal.pmed.1003305)
Supplement: S3 Table — (PDF) [file pmed.1003305.s006.pdf]

S3 Table. Association between maternal phenotypes and birth outcomes

Data set	Gestational days			Preterm birth (log(OR))			Birth weight (g)			Birth length (cm)		
	beta	se	*p*-val	beta	se	*p*-val	beta	se	*p*-val	beta	se	*p*-val
***Height* (cm)**												
ALSPAC	0.13	0.025	**3.2E-07**	-0.045	0.012	**0.00012**	16	0.91	**1.7E-66**	0.074	0.0048	**2.2E-52**
FIN	0.15	0.1	0.13	-0.018	0.012	0.15	14	1.9	**1.6E-13**	0.065	0.0087	**1.9E-13**
MoBa	0.24	0.1	**0.017**	-0.031	0.011	**0.0069**		na			na	
DNBC	0.37	0.094	**0.00011**	-0.029	0.0085	**0.00053**	15	1.7	**4.7E-17**			
HAPO	0.1	0.04	**0.013**				18	2.2	**5.0E-17**	0.08	0.0098	**7.6E-16**
GPN	0.43	0.25	0.084	-0.024	0.015	0.12	7.6	2.3	**0.00089**	0.011	0.012	0.37
meta[a]	0.14	0.02	**2.2E-12**	-0.03	0.005	**2.2E-09**	15	0.67	**1.5E-111**	0.068	0.0037	**1.6E-75**
p_het	0.097			0.6			**0.0054**			**2.1E-05**		
***BMI* (kg/m^2)**												
ALSPAC	0.099	0.045	**0.029**	-0.042	0.023	0.065	20	1.6	**2.9E-33**	0.052	0.0085	**8.6E-10**
FIN	-0.081	0.16	0.61	0.017	0.019	0.38	9.3	3	**0.0018**	0.036	0.014	**0.0086**
MoBa	-0.12	0.14	0.38	-0.0025	0.016	0.88		na			na	
DNBC	-0.012	0.14	0.93	-0.0077	0.012	0.52	13	2.5	**1.3E-07**		na	
HAPO	0.035	0.054	0.51				18	2.9	**8.7E-10**	0.039	0.013	**0.0024**
GPN	-0.25	0.36	0.48	-0.0038	0.022	0.86	7.8	3.2	**0.016**	0.054	0.018	**0.0026**
meta	0.049	0.032	0.12	-0.006	0.0075	0.42	16	1.1	**1.0E-47**	0.047	0.006	**3.9E-15**
p_het	0.5			0.41			**0.0012**			0.68		
***SBP* (mmHg)**												
ALSPAC	-0.074	0.021	**0.00038**				-4.1	0.98	**2.9E-05**	-0.014	0.0052	**0.0057**
HAPO	0.0059	0.027	0.83		na		-0.74	1.5	0.61	0.006	0.0066	0.36
meta	-0.044	0.017	**0.0073**				-3	0.81	**0.00018**	-0.0065	0.0041	0.11
p_het	**0.019**						0.055			**0.015**		
***DBP* (mmHg)**												
ALSPAC	-0.16	0.031	**1.6E-07**				-7.4	1.4	**2.9E-07**	-0.018	0.0075	**0.02**
HAPO	-0.037	0.035	0.28		na		-4.1	1.9	**0.027**	0.00075	0.0084	0.93
meta	-0.11	0.023	**3.3E-06**				-6.2	1.1	**6.0E-08**	-0.0094	0.0056	0.094
p_het	**0.008**						0.16			0.11		
***FPG* (mmol/L)[b]**												
ALSPAC	0.21	0.53	0.69	-0.058	0.26	0.82	28	19	0.15	-0.1	0.1	0.32
HAPO	-0.28	0.73	0.7		na		192	39	**5.7E-07**	0.62	0.18	**0.00048**
meta	0.043	0.43	0.92	-0.058	0.26	0.82	61	17	**0.00042**	0.082	0.089	0.36
p_het	0.59			1			**0.00011**			**0.00043**		

a: The shaded rows show the meta-analysis results. p_het: *p*-value for heterogeneity test.

b: In HAPO, maternal FPG was measured between 24-32 weeks of gestation. In ALSPAC, the FPG showed here was measured 18 years after pregnancy.

Abbreviations: BMI, body mass index; SBP and DBP, systolic and diastolic blood pressure; FPG, fasting plasma glucose; beta, estimated effect; se, standard error; log(OR), log odds ratio.
