# Supplementary material for: Dissecting maternal and fetal genetic effects underlying the associations between maternal phenotypes, birth outcomes, and adult phenotypes: A mendelian-randomization and haplotype-based genetic score analysis in 10,734 mother–infant pairs
Source: PLoS Med. 2020 Aug 25;17(8):e1003305. doi: 10.1371/journal.pmed.1003305 (PMC7447062; doi:10.1371/journal.pmed.1003305)
Supplement: S4 Table — (PDF) [file pmed.1003305.s007.pdf]

S4 Table. Associations between maternal genetic scores and maternal traits

Maternal trait	Maternal genotype (h1+h2)				Maternal trans (h1)				Maternal non-trans (h2)			
	beta	se	*p*-val	r^2	beta	se	*p*-val	r^2	beta	se	*p*-val	r^2
Height[a] (cm)	0.8	0.014	**<2.2E-308**	0.25	0.81	0.02	**<2.2E-308**	0.14	0.8	0.02	**<2.2E-308**	0.14
BMI[a] (kg/m^2)	0.87	0.037	**1.30E-120**	0.052	0.88	0.052	**1.70E-63**	0.028	0.85	0.053	**2.80E-59**	0.026
SBP[b] (mmHg)	0.27	0.025	**4.50E-27**	0.021	0.26	0.035	**4.20E-14**	0.01	0.27	0.035	**1.60E-14**	0.011
DBP[b] (mmHg)	0.32	0.028	**2.80E-30**	0.024	0.32	0.04	**8.50E-16**	0.012	0.32	0.04	**6.20E-16**	0.012
FPG[c] (mmol/L) (HAPO)	1	0.1	**1.20E-23**	0.083	1.1	0.14	**2.20E-13**	0.043	1	0.14	**1.20E-12**	0.04
FPG[c] (mmol/L) (ALSPAC)	0.89	0.088	**9.60E-24**	0.041	0.85	0.12	**8.20E-12**	0.018	0.94	0.12	**5.00E-14**	0.023
FPG[d] (mmol/L) (HAPO)	0.047	0.018	**0.0077**	0.0079	0.0067	0.025	0.79	0.00023	0.088	0.025	**0.00054**	0.012
FPG[d] (mmol/L) (ALSPAC)	0.057	0.016	**0.00027**	0.0065	0.046	0.022	**0.034**	0.0025	0.069	0.022	**0.0023**	0.0041

a: Associations between maternal height, BMI and their corresponding maternal genotype or haplotypes scores (based on the meta results of the six data sets, except birth length was not available in the DNBC data set).

b: Associations between maternal systolic (SBP) and diastolic blood pressure (DBP) and their corresponding maternal genotype or haplotypes scores based on ALSPAC and HAPO.

c: Associations between fasting plasma glucose (FPG) genetic scores and maternal FPG levels either measured during (HAPO) or 18 years after pregnancy (ALSPAC).

d: Associations between type 2 diabetes (T2D) genetic scores and maternal FPG levels either measured during (HAPO) or 18 years after pregnancy (ALSPAC).

Abbreviations: BMI, body mass index; SBP and DBP, systolic and diastolic blood pressure; FPG, fasting plasma glucose; beta, estimated effect; se, standard error; r^2, percentage of variance explained.
