# Supplementary material for: Dissecting maternal and fetal genetic effects underlying the associations between maternal phenotypes, birth outcomes, and adult phenotypes: A mendelian-randomization and haplotype-based genetic score analysis in 10,734 mother–infant pairs
Source: PLoS Med. 2020 Aug 25;17(8):e1003305. doi: 10.1371/journal.pmed.1003305 (PMC7447062; doi:10.1371/journal.pmed.1003305)
Supplement: S5 Table — (PDF) [file pmed.1003305.s008.pdf]

S5 Table. Associations between maternal height genetic scores and maternal height

Data set	geno (h1+h2)				trans (h1)				non-trans (h2)			
	beta	se	*p*-val	r^2	beta	se	*p*-val	r^2	beta	se	*p*-val	r^2
ALSPAC	0.82	0.021	**1.70E-295**	0.24	0.83	0.03	**2.40E-160**	0.12	0.82	0.03	**3.60E-152**	0.12
FIN	0.77	0.037	**2.40E-80**	0.27	0.78	0.055	**2.50E-42**	0.14	0.76	0.054	**2.10E-41**	0.13
MoBa	0.72	0.041	**5.50E-62**	0.25	0.72	0.059	**1.20E-32**	0.13	0.73	0.059	**4.10E-32**	0.12
DNBC	0.77	0.033	**7.90E-106**	0.25	0.75	0.048	**2.90E-52**	0.12	0.79	0.048	**5.10E-57**	0.13
HAPO	0.87	0.039	**7.80E-91**	0.32	0.87	0.057	**6.60E-48**	0.16	0.87	0.057	**3.80E-47**	0.16
GPN	0.92	0.098	**1.20E-18**	0.21	1	0.14	**1.60E-12**	0.12	0.83	0.13	**1.00E-09**	0.089
meta[a]	0.8	0.014	**<2.2E-308**	0.25	0.81	0.02	**<2.2E-308**	0.14	0.8	0.02	**<2.2E-308**	0.14
p_het	0.057				0.16				0.53			

a: the meta-analysis results. p_het: *p*-value for heterogeneity test.

Abbreviations: beta, estimated effect; se, standard error; r^2, percentage of variance explained.
