# Supplementary material for: Dissecting maternal and fetal genetic effects underlying the associations between maternal phenotypes, birth outcomes, and adult phenotypes: A mendelian-randomization and haplotype-based genetic score analysis in 10,734 mother–infant pairs
Source: PLoS Med. 2020 Aug 25;17(8):e1003305. doi: 10.1371/journal.pmed.1003305 (PMC7447062; doi:10.1371/journal.pmed.1003305)
Supplement: S6 Table — BMI, body mass index. (PDF) [file pmed.1003305.s009.pdf]

S6 Table. Associations between maternal BMI genetic scores and maternal BMI

Data set	geno (h1+h2)				trans (h1)				non-trans (h2)			
	beta	se	*p*-val	r^2	beta	se	*p*-val	r^2	beta	se	*p*-val	r^2
ALSPAC	0.86	0.05	**5.20E-65**	0.06	0.79	0.071	**2.10E-28**	0.025	0.94	0.071	**2.80E-39**	0.035
FIN	0.68	0.1	**7.50E-11**	0.036	0.73	0.14	**2.50E-07**	0.021	0.63	0.15	**2.10E-05**	0.015
MoBa	0.95	0.13	**2.20E-13**	0.054	0.95	0.18	**1.30E-07**	0.028	0.94	0.18	**3.20E-07**	0.026
DNBC	0.91	0.099	**1.40E-19**	0.049	1.1	0.14	**2.20E-15**	0.037	0.71	0.14	**2.00E-07**	0.015
HAPO	1	0.14	**6.40E-13**	0.047	1.2	0.2	**5.90E-09**	0.032	0.84	0.2	**2.40E-05**	0.017
GPN	1.2	0.29	**6.70E-05**	0.047	1.5	0.38	**8.50E-05**	0.044	0.75	0.4	0.06	0.0086
meta[a]	0.87	0.037	**1.30E-120**	0.052	0.88	0.052	**1.70E-63**	0.028	0.85	0.053	**2.80E-59**	0.026
p_het	0.35				0.064				0.41			

a: the meta-analysis results. p_het: *p*-value for heterogeneity test.

Abbreviations: BMI, body mass index; beta, estimated effect; se, standard error; r^2, percentage of variance explained.
