# Supplementary material for: Dissecting maternal and fetal genetic effects underlying the associations between maternal phenotypes, birth outcomes, and adult phenotypes: A mendelian-randomization and haplotype-based genetic score analysis in 10,734 mother–infant pairs
Source: PLoS Med. 2020 Aug 25;17(8):e1003305. doi: 10.1371/journal.pmed.1003305 (PMC7447062; doi:10.1371/journal.pmed.1003305)
Supplement: S7 Table — BP, blood pressure; DBP, diastolic BP; SBP, systolic BP. (PDF) [file pmed.1003305.s010.pdf]

S7 Table. Associations between maternal BP genetic scores and maternal BP (SBP and DBP)

Data set[a]	geno (h1+h2)				trans (h1)				non-trans (h2)			
	beta	se	*p*-val	r^2	beta	se	*p*-val	r^2	beta	se	*p*-val	r^2
SBP												
ALSPAC	0.25	0.026	**5.20E-22**	0.018	0.24	0.037	**5.20E-11**	0.0093	0.26	0.037	**1.50E-12**	0.0086
HAPO	0.39	0.078	**6.80E-07**	0.022	0.45	0.11	**3.80E-05**	0.015	0.33	0.11	**0.0028**	0.0074
meta	0.27	0.025	**4.50E-27**	0.021	0.26	0.035	**4.20E-14**	0.01	0.27	0.035	**1.60E-14**	0.011
p_het	0.089				0.067				0.56			
DBP												
ALSPAC	0.32	0.029	**1.50E-26**	0.02	0.31	0.042	**3.90E-13**	0.01	0.33	0.042	**6.10E-15**	0.0097
HAPO	0.41	0.1	**5.60E-05**	0.013	0.54	0.14	**0.00017**	0.011	0.28	0.14	**0.042**	0.0037
meta[b]	0.32	0.028	**2.80E-30**	0.024	0.32	0.04	**8.50E-16**	0.012	0.32	0.04	**6.20E-16**	0.012
p_het	0.38				0.11				0.77			

a: BP data were only available in ALSPAC and HAPO.

b: the meta-analysis results. p_het: *p*-value for heterogeneity test.

Abbreviations: BP, blood pressure; DBP, diastolic BP; SBP, systolic BP; beta, estimated effect; se, standard error; r^2, percentage of variance explained.
