# Supplementary material for: Dissecting maternal and fetal genetic effects underlying the associations between maternal phenotypes, birth outcomes, and adult phenotypes: A mendelian-randomization and haplotype-based genetic score analysis in 10,734 mother–infant pairs
Source: PLoS Med. 2020 Aug 25;17(8):e1003305. doi: 10.1371/journal.pmed.1003305 (PMC7447062; doi:10.1371/journal.pmed.1003305)
Supplement: S8 Table — FPG, fasting plasma glucose; T2D, type 2 diabetes. (PDF) [file pmed.1003305.s011.pdf]

S8 Table. Associations between maternal FPG and T2D genetic scores and maternal FPG

Data set[a]	geno (h1+h2)				trans (h1)				non-trans (h2)			
	beta	se	*p*-val	r^2	beta	se	*p*-val	r^2	beta	se	*p*-val	r^2
FPG[b]												
ALSPAC	0.89	0.088	**9.60E-24**	0.041	0.85	0.12	**8.20E-12**	0.018	0.94	0.12	**5.00E-14**	0.023
HAPO	1	0.1	**1.20E-23**	0.083	1.1	0.14	**2.20E-13**	0.043	1	0.14	**1.20E-12**	0.04
meta[d]	0.95	0.066	**4.60E-47**	0.059	0.94	0.093	**8.40E-24**	0.03	0.97	0.092	**1.50E-25**	0.032
p_het	0.3				0.26				0.72			
T2D[c]												
ALSPAC	0.057	0.016	**0.00027**	0.0065	0.046	0.022	**0.034**	0.0025	0.069	0.022	**0.0023**	0.0041
HAPO	0.047	0.018	**0.0077**	0.0079	0.0067	0.025	0.79	0.00023	0.088	0.025	**0.00054**	0.012
meta[d]	0.053	0.012	**6.70E-06**	0.0061	0.029	0.016	0.074	0.00097	0.077	0.017	**4.50E-06**	0.0063
p_het	0.68				0.24				0.57			

a: Maternal FPG during pregnancy was only available in HAPO. FPG was measure in a subset of ALSPAC mothers 18 years after the pregnancy.

b: Associations between FPG genetic scores (based on 22 FPG SNPs) and maternal FPG levels either measured during (HAPO) or 18 years after pregnancy (ALSPAC).

c: Associations between T2D genetic scores (based on 306 T2D SNPS) and maternal FPG levels either measured during (HAPO) or 18 years after pregnancy (ALSPAC).

d: the meta-analysis results. p_het: *p*-value for heterogeneity test.

Abbreviations: FPG, fasting plasma glucose; T2D, type 2 diabetes; beta, estimated effect; se, standard error; r^2, percentage of variance explained.
