# Supplementary material for: Dissecting maternal and fetal genetic effects underlying the associations between maternal phenotypes, birth outcomes, and adult phenotypes: A mendelian-randomization and haplotype-based genetic score analysis in 10,734 mother–infant pairs
Source: PLoS Med. 2020 Aug 25;17(8):e1003305. doi: 10.1371/journal.pmed.1003305 (PMC7447062; doi:10.1371/journal.pmed.1003305)
Supplement: S9 Table — (PDF) [file pmed.1003305.s012.pdf]

**S9 Table. Correlation (*p*-value) between maternal genotype score ($S_{mat}$), fetal genotype score ($S_{fet}$), and the 3 haplotype genetic scores: $S_{h1}$, $S_{h2}$ and $S_{h3}$.**

**A. Height**

| score | $S_{mat}$ | $S_{fet}$ | $S_{h1}$ | $S_{h2}$ |
|---|---|---|---|---|
| $S_{fet}$ | 0.53 (**<2.2E-308**) | | | |
| $S_{h1}$ | 0.72 (**<2.2E-308**) | 0.71 (**<2.2E-308**) | | |
| $S_{h2}$ | 0.72 (**<2.2E-308**) | **0.058** (1.2e-08) | **0.042** (0.0008) | |
| $S_{h3}$ | **0.044** (3.1e-06) | 0.72 (**<2.2E-308**) | **0.019** (0.024) | **0.044** (2.7e-06) |

**B. BMI**

| score | $S_{mat}$ | $S_{fet}$ | $S_{h1}$ | $S_{h2}$ |
|---|---|---|---|---|
| $S_{fet}$ | 0.5 (**<2.2E-308**) | | | |
| $S_{h1}$ | 0.71 (**<2.2E-308**) | 0.7 (**<2.2E-308**) | | |
| $S_{h2}$ | 0.7 (**<2.2E-308**) | 0.0072 (0.23) | -0.0024 (0.41) | |
| $S_{h3}$ | 0.01 (0.15) | 0.71 (**<2.2E-308**) | 0.0018 (0.45) | 0.011 (0.14) |

**C. BP[a]**

| score | $S_{mat}$ | $S_{fet}$ | $S_{h1}$ | $S_{h2}$ |
|---|---|---|---|---|
| $S_{fet}$ | 0.5 (**<2.2E-308**) | | | |
| $S_{h1}$ | 0.71 (**<2.2E-308**) | 0.7 (**<2.2E-308**) | | |
| $S_{h2}$ | 0.7 (**<2.2E-308**) | -0.0034 (0.36) | -0.0067 (0.27) | |
| $S_{h3}$ | -0.0094 (0.22) | 0.7 (**<2.2E-308**) | -0.01 (0.14) | -0.00067 (0.48) |

**D. FPG**

| score | $S_{mat}$ | $S_{fet}$ | $S_{h1}$ | $S_{h2}$ |
|---|---|---|---|---|
| $S_{fet}$ | 0.49 (**<2.2E-308**) | | | |
| $S_{h1}$ | 0.7 (**<2.2E-308**) | 0.71 (**<2.2E-308**) | | |
| $S_{h2}$ | 0.7 (**<2.2E-308**) | -0.015 (0.063) | -0.012 (0.11) | |
| $S_{h3}$ | 0.013 (0.18) | 0.72 (**<2.2E-308**) | **0.031** (0.046) | -0.0096 (0.16) |

**E. T2D**

| score | $S_{mat}$ | $S_{fet}$ | $S_{h1}$ | $S_{h2}$ |
|---|---|---|---|---|
| $S_{fet}$ | 0.5 (**1.5e-223**) | | | |
| $S_{h1}$ | 0.7 (**<2.2E-308**) | 0.71 (**<2.2E-308**) | | |
| $S_{h2}$ | 0.71 (**<2.2E-308**) | -0.0089 (0.29) | 0.002 (0.42) | |
| $S_{h3}$ | -0.004 (0.37) | 0.71 (**<2.2E-308**) | 0.0074 (0.22) | -0.013 (0.18) |

**F. Birth weight**

| score | $S_{mat}$ | $S_{fet}$ | $S_{h1}$ | $S_{h2}$ |
|---|---|---|---|---|
| $S_{fet}$ | 0.5 (**<2.2E-308**) | | | |
| $S_{h1}$ | 0.71 (**<2.2E-308**) | 0.71 (**<2.2E-308**) | | |
| $S_{h2}$ | 0.7 (**<2.2E-308**) | 0.0019 (0.45) | 0.00047 (0.49) | |
| $S_{h3}$ | 0.0042 (0.36) | 0.71 (**<2.2E-308**) | 0.004 (0.34) | 0.0029 (0.42) |

a: BP, mean of the SBP (systolic blood pressure) and DBP (diastolic blood pressure) scores.

**Abbreviations:** BMI, body mass index; FPG, fasting plasma glucose; T2D, type 2 diabetes.
