# Supplementary material for: Dissecting maternal and fetal genetic effects underlying the associations between maternal phenotypes, birth outcomes, and adult phenotypes: A mendelian-randomization and haplotype-based genetic score analysis in 10,734 mother–infant pairs
Source: PLoS Med. 2020 Aug 25;17(8):e1003305. doi: 10.1371/journal.pmed.1003305 (PMC7447062; doi:10.1371/journal.pmed.1003305)
Supplement: S10 Table — (PDF) [file pmed.1003305.s013.pdf]

S10 Table. Association between haplotype genetic scores and birth outcomes – meta-analyses using random-effects model

Maternal trait (unit) Haplotype Score Tests	Gestational days			Preterm birth (log(OR))			Birth weight (g)			Birth length (cm)		
	beta	se	*p*-val	beta	se	*p*-val	beta	se	*p*-val	beta	se	*p*-val
**Height (cm)**												
Maternal trans (β_{h1})	0.038	0.046	0.41	-0.028	0.012	**0.019**	18	2.9	**3.40E-10**	0.078	0.024	**0.00091**
Maternal non-trans (β_{h2})	0.17	0.047	**0.00022**	-0.037	0.011	**0.00097**	5.9	1.9	**0.0024**	0.022	0.011	0.056
Paternal trans (β_{h3})	-0.068	0.081	0.4	0.023	0.011	**0.038**	14	1.6	**3.10E-19**	0.069	0.012	**5.70E-09**
Maternal effect (β_{MY})	0.21	0.093	**0.025**	-0.044	0.0098	**9.20E-06**	4.7	2.2	**0.034**	0.022	0.0076	**0.0033**
Fetal effect (β_{FY})	-0.089	0.041	**0.029**	0.015	0.012	0.23	14	2.4	**1.50E-08**	0.06	0.02	**0.0026**
**BMI (kg/m²)**												
Maternal trans (β_{h1})	-0.059	0.17	0.73	-0.065	0.041	0.11	23	5.8	**7.00E-05**	0.063	0.034	0.067
Maternal non-trans (β_{h2})	-0.11	0.17	0.52	0.015	0.041	0.71	6.4	7.9	0.42	0.079	0.032	**0.013**
Paternal trans (β_{h3})	-0.081	0.23	0.72	0.049	0.042	0.24	-6.5	5.7	0.26	0.013	0.045	0.77
Maternal effect (β_{MY})	-0.098	0.15	0.51	-0.044	0.046	0.34	19	5	**0.00016**	0.06	0.028	**0.031**
Fetal effect (β_{FY})	0.043	0.15	0.77	-0.018	0.036	0.6	4	5	0.43	-0.005	0.041	0.9
**BP (mmHg)**												
Maternal trans (β_{h1})	-0.22	0.064	**0.00067**	0.034	0.016	**0.028**	-6.4	2.5	**0.011**	-0.0025	0.022	0.91
Maternal non-trans (β_{h2})	-0.055	0.086	0.52	0.047	0.016	**0.0023**	-3.1	2.2	0.16	-0.027	0.019	0.15
Paternal trans (β_{h3})	-0.016	0.064	0.8	0.011	0.02	0.59	-5.9	2.1	**0.0053**	-0.017	0.012	0.15
Maternal effect (β_{MY})	-0.12	0.055	**0.033**	0.038	0.013	**0.0045**	-2	1.9	0.27	-0.0096	0.01	0.35
Fetal effect (β_{FY})	-0.1	0.056	0.075	-0.0035	0.013	0.8	-4.8	1.9	**0.0094**	0.0032	0.018	0.86
**FPG (mmol/L)**												
Maternal trans (β_{h1})	-3.9	1.8	**0.029**	0.6	0.43	0.16	16	80	0.84	-0.067	0.32	0.84
Maternal non-trans (β_{h2})	-3.2	1.8	0.071	0.54	0.43	0.21	270	59	**4.70E-06**	0.86	0.45	0.056
Paternal trans (β_{h3})	2.2	2.3	0.33	-0.38	0.43	0.37	-52	59	0.38	-0.13	0.32	0.69
Maternal effect (β_{MY})	-5	1.5	**0.0012**	0.77	0.37	**0.039**	170	51	**0.0011**	0.48	0.41	0.23
Fetal effect (β_{FY})	0.99	1.5	0.51	-0.14	0.36	0.7	-150	50	**0.0022**	-0.46	0.28	0.096
**T2D (log(OR))**												
Maternal trans (β_{h1})	0.013	0.3	0.97	-0.0079	0.074	0.91	-14	10	0.17	-0.065	0.056	0.25
Maternal non-trans (β_{h2})	0.05	0.31	0.87	0.011	0.075	0.89	22	26	0.4	0.0046	0.11	0.97
Paternal trans (β_{h3})	0.83	0.31	**0.0069**	-0.11	0.073	0.13	-30	11	**0.0095**	-0.038	0.072	0.6
Maternal effect (β_{MY})	-0.39	0.27	0.15	0.058	0.063	0.36	24	8.9	**0.0064**	-0.047	0.12	0.7
Fetal effect (β_{FY})	0.4	0.27	0.14	-0.067	0.062	0.28	-39	8.8	**1.30E-05**	-0.085	0.049	0.082

This table is similar to Table 2, except based on meta-analysis using random-effects model.

The cells highlighted in yellow showed significant heterogeneity and the meta-analysis using random-effects model were less significant than the fixed-effect model (Table 2). As shown in S8 Fig (top row) and S12 Fig (second row), the source of heterogeneity came from the GPN data set. The GPN data set included more early preterm pregnancies (20-34wks), which might enrich for risk factors that can reduce both gestational duration and fetal growth. After excluding the GPN data set, the Cochran's Q was no longer significant, and the *p*-values of the meta-analyses became even more significant.

Abbreviations: BP, mean of the SBP (systolic blood pressure) and DBP (diastolic blood pressure) scores; BMI, body mass index; FPG, fasting plasma glucose; T2D, type 2 diabetes; beta, estimated effect; se, standard error; log(OR), log odds ratio.
