# Supplementary material for: Dissecting maternal and fetal genetic effects underlying the associations between maternal phenotypes, birth outcomes, and adult phenotypes: A mendelian-randomization and haplotype-based genetic score analysis in 10,734 mother–infant pairs
Source: PLoS Med. 2020 Aug 25;17(8):e1003305. doi: 10.1371/journal.pmed.1003305 (PMC7447062; doi:10.1371/journal.pmed.1003305)
Supplement: S11 Table — ALSPAC, The Avon Longitudinal Study of Parents and Children. (PDF) [file pmed.1003305.s014.pdf]

S11 Table. Association between haplotype genetic scores and birth outcomes based on the ALSPAC data set only

Maternal trait (unit) Haplotype Score Tests	Gestational days			Preterm birth (log(OR))			Birth weight (g)			Birth length (cm)		
	beta	se	*p*-val	beta	se	*p*-val	beta	se	*p*-val	beta	se	*p*-val
**Height (cm)**												
Maternal trans (β_{h1})	0.022	0.06	0.71	-0.03	0.027	0.26	24	2.2	**2.40E-28**	0.11	0.011	**1.30E-23**
Maternal non-trans (β_{h2})	0.15	0.061	**0.015**	-0.046	0.028	0.098	8.1	2.2	**0.00026**	0.021	0.012	0.076
Paternal trans (β_{h3})	-0.07	0.06	0.22	0.014	0.027	0.59	13	2.2	**1.70E-09**	0.085	0.011	**5.00E-14**
Maternal effect (β_{MY})	0.12	0.05	**0.021**	-0.05	0.02	0.06	9.5	2	**6.60E-07**	0.025	0.01	**0.013**
Fetal effect (β_{FY})	-0.1	0.05	0.059	0.015	0.02	0.54	15	2	**4.20E-14**	0.09	0.01	**4.50E-19**
**BMI (kg/m^2)**												
Maternal trans (β_{h1})	-0.01	0.22	0.95	-0.096	0.1	0.34	24	8.1	**0.0032**	0.096	0.042	**0.023**
Maternal non-trans (β_{h2})	-0.02	0.22	0.94	0.12	0.1	0.25	18	8.1	**0.025**	0.085	0.042	**0.044**
Paternal trans (β_{h3})	0.25	0.22	0.25	-0.059	0.1	0.56	0.95	8	0.91	0.067	0.042	0.11
Maternal effect (β_{MY})	-0.14	0.19	0.46	0.04	0.09	0.64	20	7	**0.0033**	0.057	0.04	0.12
Fetal effect (β_{FY})	0.13	0.19	0.51	-0.14	0.09	0.12	3.3	7	0.63	0.039	0.04	0.28
**BP (mmHg)**												
Maternal trans (β_{h1})	-0.24	0.083	**0.0039**	0.082	0.038	**0.029**	-8.6	3	**0.004**	-0.044	0.016	**0.005**
Maternal non-trans (β_{h2})	-0.03	0.084	0.68	0.061	0.038	0.11	-2.4	3	0.42	0.0062	0.016	0.69
Paternal trans (β_{h3})	-0.06	0.082	0.45	0.07	0.037	0.06	-7.6	3	**0.01**	-0.013	0.016	0.39
Maternal effect (β_{MY})	-0.11	0.07	0.14	0.037	0.03	0.26	-1.7	3	0.51	-0.012	0.01	0.36
Fetal effect (β_{FY})	-0.13	0.07	0.066	0.046	0.03	0.17	-6.9	3	**0.0082**	-0.032	0.01	**0.02**
**FPG (mmol/L)**												
Maternal trans (β_{h1})	-3.3	2.3	0.14	-0.27	1	0.79	12	82	0.89	-0.16	0.43	0.72
Maternal non-trans (β_{h2})	-2.8	2.3	0.22	1.1	1	0.29	240	82	**0.0039**	0.18	0.43	0.67
Paternal trans (β_{h3})	4.9	2.3	**0.03**	-1.5	1	0.16	-110	82	0.2	0.0064	0.43	0.99
Maternal effect (β_{MY})	-5.5	2	**0.005**	1.1	0.9	0.2	180	70	**0.012**	0.01	0.37	0.98
Fetal effect (β_{FY})	2.2	2	0.27	-1.4	0.9	0.11	-170	71	**0.019**	-0.17	0.37	0.65
**T2D (log(OR))**												
Maternal trans (β_{h1})	0.29	0.39	0.46	0.0075	0.18	0.97	-11	14	0.45	-0.029	0.074	0.7
Maternal non-trans (β_{h2})	0.046	0.41	0.91	-0.016	0.18	0.93	41	15	**0.0056**	0.092	0.076	0.23
Paternal trans (β_{h3})	0.75	0.4	0.062	-0.36	0.18	**0.049**	-15	14	0.3	-0.0042	0.075	0.96
Maternal effect (β_{MY})	-0.21	0.34	0.55	0.17	0.16	0.26	22	12	0.07	0.034	0.07	0.6
Fetal effect (β_{FY})	0.5	0.35	0.15	-0.17	0.16	0.29	-33	12	**0.0078**	-0.063	0.07	0.33

This table is similar to Table 2, except based on the ALSPAC data set only.

Abbreviations: BP, mean of the SBP (systolic blood pressure) and DBP (diastolic blood pressure) scores; BMI, body mass index; FPG, fasting plasma glucose; T2D, type 2 diabetes; beta, estimated effect; se, standard error; log(OR), log odds ratio.
