# Supplementary material for: Dissecting maternal and fetal genetic effects underlying the associations between maternal phenotypes, birth outcomes, and adult phenotypes: A mendelian-randomization and haplotype-based genetic score analysis in 10,734 mother–infant pairs
Source: PLoS Med. 2020 Aug 25;17(8):e1003305. doi: 10.1371/journal.pmed.1003305 (PMC7447062; doi:10.1371/journal.pmed.1003305)
Supplement: S12 Table — DNBC, The Danish National Birth Cohort; FIN, The Finnish birth data set; GPN, The Genomic and Proteomic Network for Preterm Birth Research; HAPO, Hyperglycemia and Adverse Pregnancy Outcome; MoBa, The Mother Child data set of Norway. (PDF) [file pmed.1003305.s015.pdf]

S12 Table. Association between haplotype genetic scores and birth outcomes based on meta-analysis of the FIN, MoBa, DNBC, HAPO and GPN data sets

Maternal trait (unit) Haplotype Score Tests	Gestational days			Preterm birth (log(OR))			Birth weight (g)			Birth length (cm)		
	beta	se	*p*-val	beta	se	*p*-val	beta	se	*p*-val	beta	se	*p*-val
**Height (cm)**												
Maternal trans (β_{h1})	0.061	0.073	0.4	-0.027	0.012	**0.033**	16	2.3	**1.60E-12**	0.072	0.013	**7.80E-08**
Maternal non-trans (β_{h2})	0.21	0.073	**0.005**	-0.036	0.012	**0.004**	4.4	2.3	0.055	0.024	0.013	0.064
Paternal trans (β_{h3})	0.0022	0.07	0.98	0.025	0.012	**0.042**	15	2.2	**2.60E-11**	0.059	0.013	**5.60E-06**
Maternal effect (β_{MY})	0.13	0.06	**0.034**	-0.043	0.01	**6.10E-05**	2.8	2	0.16	0.019	0.01	0.1
Fetal effect (β_{FY})	-0.07	0.06	0.25	0.017	0.01	0.13	13	2	**3.70E-11**	0.053	0.01	**6.00E-06**
**BMI (kg/m^2)**												
Maternal trans (β_{h1})	-0.12	0.27	0.64	-0.059	0.045	0.19	22	8.3	**0.0075**	0.027	0.048	0.58
Maternal non-trans (β_{h2})	-0.25	0.27	0.36	-0.0053	0.045	0.91	-1.3	8.4	0.88	0.072	0.05	0.15
Paternal trans (β_{h3})	-0.32	0.27	0.24	0.07	0.044	0.12	-14	8.2	0.081	-0.027	0.049	0.57
Maternal effect (β_{MY})	-0.03	0.24	0.89	-0.066	0.04	0.091	18	7	**0.016**	0.064	0.04	0.14
Fetal effect (β_{FY})	-0.09	0.24	0.71	0.005	0.04	0.9	4.6	7	0.52	-0.035	0.04	0.41
**BP^b (mmHg)**												
Maternal trans (β_{h1})	-0.19	0.1	0.066	0.024	0.017	0.15	-4.9	3.1	0.12	0.016	0.018	0.38
Maternal non-trans (β_{h2})	-0.036	0.1	0.72	0.045	0.017	**0.009**	-3.7	3.1	0.23	-0.049	0.018	**0.006**
Paternal trans (β_{h3})	0.052	0.1	0.61	-0.0082	0.017	0.63	-4.1	3.1	0.18	-0.022	0.018	0.22
Maternal effect (β_{MY})	-0.14	0.09	0.12	0.038	0.02	**0.0089**	-2.4	3	0.37	-0.006	0.02	0.71
Fetal effect (β_{FY})	-0.05	0.09	0.57	-0.013	0.02	0.37	-2.7	3	0.31	0.021	0.02	0.17
**FPG (mmol/L)**												
Maternal trans (β_{h1})	-4.7	2.8	0.095	0.78	0.47	0.095	15	85	0.86	0.051	0.49	0.92
Maternal non-trans (β_{h2})	-3.8	2.8	0.17	0.43	0.47	0.36	300	85	**0.00032**	1.4	0.49	**0.0048**
Paternal trans (β_{h3})	-0.53	2.7	0.85	-0.17	0.47	0.72	6.1	85	0.94	-0.3	0.49	0.54
Maternal effect (β_{MY})	-4	2.5	0.1	0.69	0.41	0.091	160	75	**0.035**	0.87	0.43	**0.045**
Fetal effect (β_{FY})	-0.73	2.4	0.76	0.11	0.4	0.78	-140	71	**0.046**	-0.83	0.42	**0.045**
**T2D (log(OR))**												
Maternal trans (β_{h1})	-0.4	0.48	0.4	-0.011	0.081	0.89	-18	15	0.23	-0.11	0.086	0.19
Maternal non-trans (β_{h2})	0.056	0.47	0.91	0.018	0.079	0.81	26	14	0.068	0.017	0.084	0.84
Paternal trans (β_{h3})	0.96	0.49	**0.047**	-0.063	0.08	0.43	-42	15	**0.0042**	-0.072	0.087	0.4
Maternal effect (β_{MY})	-0.65	0.42	0.12	0.035	0.07	0.61	26	13	**0.041**	-0.01	0.07	0.9
Fetal effect (β_{FY})	0.25	0.42	0.56	-0.048	0.07	0.48	-44	13	**0.00043**	-0.11	0.07	0.12

This table is similar to Table 2, except based on the meta-analysis of FIN, MoBa, DNBC, HAPO and GPN data sets.

Abbreviations: BP, mean of the SBP (systolic blood pressure) and DBP (diastolic blood pressure) scores; BMI, body mass index; FPG, fasting plasma glucose; T2D, type 2 diabetes; beta, estimated effect; se, standard error; log(OR), log odds ratio.
