# Supplementary material for: Dissecting maternal and fetal genetic effects underlying the associations between maternal phenotypes, birth outcomes, and adult phenotypes: A mendelian-randomization and haplotype-based genetic score analysis in 10,734 mother–infant pairs
Source: PLoS Med. 2020 Aug 25;17(8):e1003305. doi: 10.1371/journal.pmed.1003305 (PMC7447062; doi:10.1371/journal.pmed.1003305)
Supplement: S13 Table — SD, standard deviation. (PDF) [file pmed.1003305.s016.pdf]

S13 Table. Phenotypic associations, estimated maternal causal effects and genetically confounded associations (per 1-SD changes in maternal traits) between maternal traits and birth outcomes

Maternal trait (1-SD)	Gestational days			Preterm birth (log(OR))			Birth weight (g)			Birth length (cm)		
	beta	se	*p*-val	beta	se	*p*-val	beta	se	*p*-val	beta	se	*p*-val
**Height (1SD=6.4cm)**												
Phenotypic	0.898	0.128	**2.20E-12**	-0.193	0.0322	**2.20E-09**	96.2	4.29	**1.50E-111**	0.433	0.0235	**1.60E-75**
Causal (TSLS)	1.29	0.351	**0.00024**	-0.298	0.0904	**0.00099**	57.8	12.3	**2.40E-06**	0.207	0.0671	**0.002**
Causal (Ratio)	1.01	0.323	**0.0018**	-0.348	0.0787	**9.60E-06**	50.3	11.1	**5.60E-06**	0.178	0.0607	**0.0033**
Fetal genetic	-0.711	0.326	**0.029**	0.13	0.0794	0.1	111	11.3	**5.00E-23**	0.59	0.0617	**1.00E-21**
Confounded	-0.289	0.133	**0.029**	0.0528	0.0323	0.1	45.3	4.59	**5.00E-23**	0.24	0.0251	**1.00E-21**
Combined[a]	0.722	0.349	**0.039**	-0.296	0.0851	**0.00051**	95.6	12	**1.50E-15**	0.418	0.0657	**1.90E-10**
**BMI (1SD=4.0kg/m^2)**												
Phenotypic	0.196	0.127	0.12	-0.0241	0.03	0.42	62.4	4.3	**1.00E-47**	0.187	0.0238	**3.90E-15**
Causal (TSLS)	-0.222	0.791	0.78	0.0735	0.219	0.74	59.4	28.4	**0.036**	0.339	0.152	**0.026**
Causal (Ratio)	-0.452	0.686	0.51	-0.222	0.165	0.18	87.7	23.5	**0.00019**	0.275	0.128	**0.032**
Fetal genetic	0.197	0.686	0.77	-0.0845	0.163	0.6	18.2	23	0.43	0.0356	0.127	0.78
Confounded	0.0511	0.178	0.77	-0.022	0.0425	0.6	4.74	5.97	0.43	0.00925	0.033	0.78
Combined	-0.401	0.708	0.57	-0.244	0.17	0.15	92.4	24.2	**0.00014**	0.284	0.132	**0.032**
**BP (1SD=5.8mmHg)**												
Phenotypic	-0.487	0.125	**9.20E-05**				-30.2	6.11	**8.00E-07**	-0.0549	0.0306	0.072
Causal (TSLS)	1.71	1.23	0.16				-73.9	57.3	0.2	-0.18	0.313	0.56
Causal (Ratio)	-2.28	1.09	**0.036**	0.73	0.264	**0.0057**	-39.2	36	0.28	-0.186	0.199	0.35
Fetal genetic	-1.93	1.1	0.078	-0.0668	0.259	0.8	-93.5	36.8	**0.011**	-0.166	0.199	0.41
Confounded	-0.228	0.129	0.078	-0.0079	0.0306	0.8	-11.1	4.35	**0.011**	-0.0196	0.0235	0.41
Combined	-2.51	1.09	**0.022**	0.722	0.266	**0.0066**	-50.2	36.2	0.17	-0.206	0.201	0.31
**FPG (1SD=0.36mmol/L)**												
Phenotypic	-0.102	0.264	0.7				70.4	14	**4.90E-07**	0.223	0.0636	**0.00046**
Causal (TSLS)	-0.731	1.26	0.56				147	67.5	**0.03**	0.672	0.309	**0.03**
Causal (Ratio)	-1.73	0.561	**0.002**	0.269	0.133	**0.043**	58.7	18.8	**0.0018**	0.13	0.0992	0.19
Fetal genetic	0.346	0.529	0.51	-0.0482	0.127	0.7	-53.8	18.3	**0.0033**	-0.161	0.0977	0.1
Confounded	0.0865	0.132	0.51	-0.0121	0.0317	0.7	-13.5	4.58	**0.0033**	-0.0402	0.0244	0.1
Combined	-1.64	0.577	**0.0043**	0.257	0.137	0.06	45.3	19.4	**0.019**	0.0894	0.102	0.38

a: "*Combined*" is the sum of maternal "*Causal (Ratio)*" and genetically "*Confounded*" effect.

Abbreviations: BP, mean of the SBP (systolic blood pressure) and DBP (diastolic blood pressure) scores; BMI, body mass index; FPG, fasting plasma glucose; beta, estimated effect; se, standard error; log(OR), log odds ratio; SD, standard deviation.
