# Supplementary material for: Dissecting maternal and fetal genetic effects underlying the associations between maternal phenotypes, birth outcomes, and adult phenotypes: A mendelian-randomization and haplotype-based genetic score analysis in 10,734 mother–infant pairs
Source: PLoS Med. 2020 Aug 25;17(8):e1003305. doi: 10.1371/journal.pmed.1003305 (PMC7447062; doi:10.1371/journal.pmed.1003305)
Supplement: S14 Table — SD, standard deviation. (PDF) [file pmed.1003305.s017.pdf]

S14 Table. Estimated changes in adult phenotypes per 1-SD changes in birth outcomes

Adult phenotype	Gestational days (1-SD=11.4day)			Preterm birth (1-SD=1.81 log[OR])			Birth weight (1-SD=426g)			Birth length (1-SD=2.2cm)		
	beta	se	*p*-val	beta	se	*p*-val	beta	se	*p*-val	beta	se	*p*-val
**Height (cm)**												
Method 1	-0.074	0.131	0.57	-0.114	0.228	0.62	1.31	0.119	**3.40E-28**	1.25	0.125	**1.50E-23**
Method 2	-0.00658	0.0926	0.94	-0.043	0.162	0.79	1.32	0.0848	**1.10E-54**	1.26	0.0887	**1.50E-45**
**BMI (kg/m^2)**												
Method 1	-0.0054	0.14	0.97	-0.257	0.24	0.28	0.304	0.126	**0.016**	0.162	0.133	0.22
Method 2	-0.0106	0.101	0.92	-0.0578	0.175	0.74	0.186	0.0914	**0.042**	0.201	0.0961	**0.037**
**BP (mmHg)**												
Method 1	-0.331	0.131	**0.011**	0.231	0.224	0.3	-0.327	0.116	**0.0049**	-0.143	0.123	0.24
Method 2	-0.249	0.0956	**0.0093**	0.305	0.164	0.063	-0.359	0.085	**2.50E-05**	-0.191	0.0902	**0.035**
**FPG (mmol/L)**												
Method 1	-0.00892	0.0102	0.38	0.0106	0.0176	0.54	-0.0112	0.00906	0.21	-0.00842	0.00951	0.38
Method 2	-0.00395	0.00744	0.6	0.00605	0.0128	0.64	-0.00278	0.00664	0.68	-0.00304	0.00699	0.66
**T2D (log(OR))**												
Method 1	0.0263	0.0385	0.49	-0.0348	0.0648	0.59	-0.0917	0.0342	**0.0073**	-0.0511	0.036	0.16
Method 2	0.055	0.028	**0.049**	-0.056	0.048	0.24	-0.0723	0.0252	**0.004**	-0.0334	0.0265	0.21

The genetically confounded association was estimated by two methods (S1 Text):

$$\hat{\beta}^{c}_{YX'} = h^2 \frac{\mathrm{Var}(X')}{\mathrm{Var}(Y)}\left(\frac{\hat{\beta}_{MY}}{2} + \hat{\beta}_{FY}\right) \text{ (Method 1) and } \hat{\beta}^{c}_{YX'} = h^2 \frac{\mathrm{Var}(X')}{\mathrm{Var}(Y)}\left(\frac{\widehat{\beta_{h1}+\beta_{h3}}}{2}\right) \text{ (Method 2)}$$

Abbreviations: BP, mean of the SBP (systolic blood pressure) and DBP (diastolic blood pressure) scores; BMI, body mass index; FPG, fasting plasma glucose; T2D, type 2 diabetes; beta, estimated effect; se, standard error; log(OR), log odds ratio; SD, standard deviation.
