# Supplementary material for: Dissecting maternal and fetal genetic effects underlying the associations between maternal phenotypes, birth outcomes, and adult phenotypes: A mendelian-randomization and haplotype-based genetic score analysis in 10,734 mother–infant pairs
Source: PLoS Med. 2020 Aug 25;17(8):e1003305. doi: 10.1371/journal.pmed.1003305 (PMC7447062; doi:10.1371/journal.pmed.1003305)
Supplement: S15 Table — (PDF) [file pmed.1003305.s018.pdf]

S15 Table. Association between fetal birth weight genetic score and gestational-age-adjusted birth weight (gram)

Data set	geno (h1+h2)				maternal-trans (h1)				paternal-trans (h3)				diff (h1-h3)[b]			
	beta	se	*p*-val	r^2	beta	se	*p*-val	r^2	beta	se	*p*-val	r^2	beta	se	*p*-val	chi
ALSPAC	1.2	0.085	**3.90E-41**	0.037	0.85	0.12	**3.40E-12**	0.011	1.4	0.12	**3.10E-33**	0.028	-0.59	0.17	**0.00055**	12
FIN	0.58	0.16	**0.00025**	0.012	0.35	0.22	0.1	0.0029	0.84	0.23	**0.00031**	0.01	-0.48	0.32	0.13	2.3
DNBC	0.9	0.16	**7.30E-09**	0.021	0.83	0.21	**0.00012**	0.0089	0.98	0.22	**8.90E-06**	0.012	-0.15	0.3	0.62	0.25
HAPO	1.3	0.19	**6.10E-11**	0.038	0.9	0.27	**0.00091**	0.012	1.6	0.27	**4.40E-09**	0.028	-0.72	0.39	0.064	3.4
GPN	1.1	0.23	**3.00E-06**	0.069	1.1	0.35	**0.0016**	0.033	1.1	0.3	**0.00057**	0.036	0.047	0.46	0.92	0.01
meta[a]	1	0.061	**5.60E-64**	0.031	0.79	0.087	**1.20E-19**	0.0092	1.3	0.086	**3.90E-49**	0.024	-0.47	0.12	**0.00013**	0.0017
p_het	**0.015**				0.26				**0.049**				0.51			

a: shows the meta-analysis results. p_het: *p*-value for heterogeneity test.

b: tests the difference in effect size between the maternal transmitted (h1) and the paternal transmitted (h3) haplotype scores; chi: chi-squared statistics of the difference between h1 and h2.

Abbreviations: beta, estimated effect; se, standard error; r^2, percentage of variance explained.
