# Supplementary material for: Dissecting maternal and fetal genetic effects underlying the associations between maternal phenotypes, birth outcomes, and adult phenotypes: A mendelian-randomization and haplotype-based genetic score analysis in 10,734 mother–infant pairs
Source: PLoS Med. 2020 Aug 25;17(8):e1003305. doi: 10.1371/journal.pmed.1003305 (PMC7447062; doi:10.1371/journal.pmed.1003305)
Supplement: S16 Table — MR-PRESSO, mendelian randomization pleiotropy residual sum and outlier. (PDF) [file pmed.1003305.s019.pdf]

S16 Table. MR-PRESSO: Effects of maternal traits on birth outcomes

		Gestational days			Preterm birth			Birth weight			Birth length		
Trait	Method[a]	beta	se	*p*-val	beta	se	*p*-val	beta	se	*p*-val	beta	se	*p*-val
Height													
h1[b]	raw	0.038	0.056	0.5	-0.030	0.015	0.05	28.13	1.88	**7.90E-44**	0.133	0.010	**2.90E-35**
	corrected	0.078	0.055	0.16	-0.027	0.015	0.077	26.56	1.88	**7.60E-45**	0.131	0.010	**7.00E-36**
h2	raw	0.203	0.056	**0.00029**	-0.056	0.015	**0.00016**	9.84	1.88	**2.90E-07**	0.033	0.010	**0.0017**
	corrected	0.203	0.055	**0.00021**	-0.058	0.015	**9.80E-05**	10.00	1.88	**1.00E-07**	0.034	0.010	**0.0006**
h3	raw	-0.067	0.056	0.23	0.030	0.015	**0.044**	18.75	1.88	**2.00E-20**	0.103	0.011	**6.30E-22**
	corrected	-0.064	0.055	0.24	0.028	0.015	0.055	18.75	1.88	**1.30E-21**	0.102	0.010	**4.90E-22**
BMI (Body mass index)													
h1	raw	-0.075	0.1875	0.69	-0.0625	0.05	0.2	24.25	6.75	**0.00038**	0.08	0.035	**0.025**
	corrected	-0.045	0.18	0.81	-0.0575	0.05	0.23	23	6.5	**0.00045**	0.0575	0.035	0.097
h2	raw	-0.1	0.1875	0.6	0.055	0.05	0.27	12.25	6.5	0.056	0.0825	0.035	**0.02**
	corrected	-0.09	0.18	0.61	0.0575	0.0475	0.23	10	6.25	0.11	0.095	0.035	**0.0053**
h3	raw	0.16	0.1925	0.4	0.01025	0.0525	0.84	-4	6.5	0.54	0.0475	0.035	0.19
	corrected	0.22	0.1825	0.23	0.01425	0.05	0.77	-3.5	6	0.55	0.0425	0.0325	0.21
BP (Blood pressure)													
h1	raw	-0.25	0.073	**0.00078**	0.056	0.02	**0.0045**	-7.5	2.4	**0.002**	-0.028	0.013	**0.036**
	corrected	-0.22	0.069	**0.0015**	0.055	0.019	**0.0044**	-7.5	2.3	**0.0012**	-0.03	0.013	**0.018**
h2	raw	-0.03	0.07	0.67	0.058	0.019	**0.0025**	-3.1	2.4	0.19	-0.0093	0.013	0.48
	corrected	-0.029	0.068	0.67	0.057	0.019	**0.0028**	-3.6	2.3	0.12	-0.014	0.013	0.27
h3	raw	-0.029	0.07	0.68	0.016	0.018	0.38	-6.9	2.4	**0.0044**	-0.016	0.013	0.2
	corrected	-0.058	0.068	0.4	0.015	0.018	0.39	-8.3	2.3	**0.00035**	-0.012	0.013	0.33
FPG (Fasting plasma glucose)													
h1	raw	-3.4	2.1	0.11	0.19	0.67	0.78	14	62	0.82	-0.11	0.3	0.72
	corrected	-2.7	1.9	0.17	0.15	0.51	0.78	-12	53	0.82			
h2	raw	-2.9	2.2	0.2	0.59	0.52	0.27	270	74	**0.0018**	0.54	0.27	0.056
	corrected	-2.8	1.4	0.058	NA	NA	NA	250	69	**0.0016**	NA	NA	NA
h3	raw	3.8	1.7	**0.037**	-0.5	0.52	0.34	-74	77	0.35	-0.07	0.38	0.86
	corrected	NA	NA	NA	NA	NA	NA	-43	59	0.48	0.039	0.35	0.91
T2D (Type 2 diabetes)													
h1	raw	0.02	0.30	0.96	-0.009	0.074	0.9	-11.7	11.3	0.3	-0.069	0.056	0.2
	corrected	NA	NA	NA	NA	NA	NA	-13.5	11.3	0.22	NA	NA	NA
h2	raw	0.05	0.31	0.88	0.014	0.078	0.85	33.9	10.4	**0.0014**	0.039	0.052	0.46
	corrected	NA	NA	NA	NA	NA	NA	33.4	10.4	**0.0012**	NA	NA	NA
h3	raw	0.83	0.32	**0.011**	-0.104	0.078	0.17	-28.2	10.0	**0.0064**	-0.048	0.052	0.35
	corrected	NA	NA	NA	NA	NA	NA	NA	NA	NA	NA	NA	NA

a: The raw estimates were computed based on all GWA SNPs using standard IVW (inverse-variance weighted) analysis. The corrected estimates were calculated after excluding outliers only when the MR-PRESSO global test suggested horizontal pleiotropy ($p < 0.05$) (please see S18 Table).

b: The beta calculated based on h1, h2 or h3 may not reflect real causal effect. It indicates the slope of the regression line between the reported effects of the SNPs on an adult phenotype and the estimated allele-specific effects on a pregnancy outcome, i.e. it evaluates the allele-specific genetic effect of one-unit change in a maternal (adult) phenotype corresponds to how many units change in an outcome. In the analysis of blood pressure (BP), the averages of reported effects on SBP and DBP were used.

Abbreviations: beta, estimated effect; se, standard error.
