# Supplementary material for: Dissecting maternal and fetal genetic effects underlying the associations between maternal phenotypes, birth outcomes, and adult phenotypes: A mendelian-randomization and haplotype-based genetic score analysis in 10,734 mother–infant pairs
Source: PLoS Med. 2020 Aug 25;17(8):e1003305. doi: 10.1371/journal.pmed.1003305 (PMC7447062; doi:10.1371/journal.pmed.1003305)
Supplement: S17 Table — MR-PRESSO: effects of fetal growth on birth outcomes (A) and maternal BP and glucose levels (B). BP, blood pressure; MR-PRESSO, mendelian randomization pleiotropy residual sum and outlier. (PDF) [file pmed.1003305.s020.pdf]

S17 Table. MR-PRESSO: Effects of fetal growth on birth outcomes (A) and maternal BP and glucose levels (B)

A. Effects of birth weight associated SNPs on birth outcomes

		Gestational days			Preterm birth			Birth weight			Birth length		
Birth weight	Method[a]	beta	se	*p*-val	beta	se	*p*-val	beta	se	*p*-val	beta	se	*p*-val
h1	raw	-0.0058	0.0029	0.056	0.0025	0.0007	**0.00074**	0.74	0.10	**1.8E-11**	0.0017	0.0005	**0.0008**
	corrected	NA	NA	NA	NA	NA	NA	NA	NA	**5.8E-12**	NA	NA	NA
h2	raw	0.0010	0.0027	0.7	-0.0005	0.0007	0.44	-0.17	0.11	0.11	-0.0003	0.0005	0.56
	corrected	NA	NA	NA	NA	NA	NA	NA	NA	**0.012**	NA	NA	NA
h3	raw	-0.0101	0.0031	**0.0012**	0.0020	0.0006	**0.003**	1.22	0.08	**6.5E-25**	0.0029	0.0004	**1.7E-09**
	corrected	NA	NA	NA	NA	NA	NA	NA	NA	NA	NA	NA	NA

B. Effects of birth weight associated SNPs on maternal BP and glucose levels

		BP[b]			SBP[b]			DBP[b]			FPG[c]		
Birth weigth	Method[a]	beta	se	*p*-val	beta	se	*p*-val	beta	se	*p*-val	beta	se	*p*-val
h1	raw	-0.0016	0.0014	0.28	-0.0029	0.0018	0.12	-0.0003	0.0015	0.85	-0.0002	0.0002	0.34
	corrected	NA	NA	NA	NA	NA	NA	NA	NA	NA	NA	NA	0.63
h2	raw	0.0005	0.0012	0.66	0.0006	0.0016	0.72	0.0007	0.0012	0.57	-0.0003	0.0002	0.092
	corrected	NA	NA	NA	NA	NA	NA	NA	NA	NA	NA	NA	0.19
h3	raw	0.0033	0.0013	**0.014**	0.0043	0.0017	**0.011**	0.0021	0.0013	0.11	-0.0001	0.0001	0.68
	corrected	NA	NA	NA	NA	NA	NA	NA	NA	NA	NA	NA	NA

a: The raw estimates were computed based on all GWA SNPs using standard IVW (inverse-variance weighted) analysis. The corrected estimates were calculated after excluding outliers only when the MR-PRESSO global test suggested horizontal pleiotropy ($p < 0.05$) (please see S19 Table).

b: Maternal blood pressures (SBP: systolic blood pressure, DBP: diastolic blood pressure and BP: average of SBP and DBP) in ALSPAC and HAPO.

c: Fasting plasma glucose (FPG) during pregnancy measured in HAPO.

Abbreviations: beta, estimated effect; se, standard error.
