# Supplementary material for: Dissecting maternal and fetal genetic effects underlying the associations between maternal phenotypes, birth outcomes, and adult phenotypes: A mendelian-randomization and haplotype-based genetic score analysis in 10,734 mother–infant pairs
Source: PLoS Med. 2020 Aug 25;17(8):e1003305. doi: 10.1371/journal.pmed.1003305 (PMC7447062; doi:10.1371/journal.pmed.1003305)
Supplement: S18 Table — MR-PRESSO, mendelian randomization pleiotropy residual sum and outlier. (PDF) [file pmed.1003305.s021.pdf]

S18 Table. MR-PRESSO global test, outlier test, and distortion test results: effects of maternal traits on birth outcomes

	Gestational days			Preterm birth			Birth weight			Birth length		
	global *p*-val	number of outliers	distortion *p*-val	global *p*-val	number of outliers	distortion *p*-val	global *p*-val	number of outliers	distortion *p*-val	global *p*-val	number of outliers	distortion *p*-val
Height (2130)[a]												
**h1**	<0.001	17	0.636	<0.001	9	0.808	<0.001	20	0.775	<0.001	14	0.889
**h2**	<0.001	19	0.974	<0.001	5	0.942	<0.001	11	0.923	<0.001	14	0.815
**h3**	<0.001	19	0.936	<0.001	6	0.893	<0.001	17	0.975	<0.001	10	0.882
BMI (628)												
**h1**	<0.001	8	0.632	<0.001	1	0.902	<0.001	11	0.831	<0.001	7	0.348
**h2**	<0.001	9	0.958	<0.001	5	0.948	<0.001	7	0.653	<0.001	7	0.718
**h3**	<0.001	12	0.811	<0.001	5	0.941	<0.001	11	0.952	<0.001	7	0.886
BP (831)												
**h1**	<0.001	17	0.643	<0.001	8	0.956	<0.001	17	0.993	<0.001	14	0.865
**h2**	<0.001	11	0.995	<0.001	1	0.955	<0.001	8	0.864	<0.001	13	0.785
**h3**	<0.001	11	0.771	<0.001	2	0.970	<0.001	13	0.607	<0.001	8	0.625
FPG (22)												
**h1**	0.004	1	0.567	<0.001	2	0.872	0.029	1	0.087	0.137	NA	NA
**h2**	<0.001	3	0.936	0.137	NA	NA	0.001	1	0.803	0.47	NA	NA
**h3**	0.052	NA	NA	0.139	NA	NA	<0.001	1	0.262	0.005	1	0.089
T2D (306)												
**h1**	0.75	0	NA	0.725	0	NA	<0.001	1	0.909	0.177	0	NA
**h2**	0.481	0	NA	0.166	0	NA	0.016	2	0.971	0.58	0	NA
**h3**	0.131	0	NA	0.116	0	NA	0.064	0	NA	0.565	0	NA

This table shows the results of MR-PRESSO global test, number of MR-PRESSO outliers and MR-PRESSO distortion test of the MR-PRESSO analysis of the effects of maternal traits on birth outcomes (S16 Table).

a: The numbers in the parentheses are the numbers of SNPs used in the MR-PRESSO analyses. It can be shown that although global horizontal pleiotropy was detected in some of the MR analyses, the numbers of variants with significant horizontal pleiotropic effect were very small and none of the causal effects had significant distortions due to the horizontal pleiotropic outlier variants.

Abbreviations: BP, mean of the SBP (systolic blood pressure) and DBP (diastolic blood pressure) scores; BMI, body mass index; FPG, fasting plasma glucose; T2D, type 2 diabetes.
