# Supplementary material for: Dissecting maternal and fetal genetic effects underlying the associations between maternal phenotypes, birth outcomes, and adult phenotypes: A mendelian-randomization and haplotype-based genetic score analysis in 10,734 mother–infant pairs
Source: PLoS Med. 2020 Aug 25;17(8):e1003305. doi: 10.1371/journal.pmed.1003305 (PMC7447062; doi:10.1371/journal.pmed.1003305)
Supplement: S19 Table — MR-PRESSO global test, outlier test, and distortion test results: effects of fetal growth on birth outcomes (A) and maternal BP and glucose levels (B). BP, blood pressure; MR-PRESSO, mendelian randomization pleiotropy residual sum and outlier. (PDF) [file pmed.1003305.s022.pdf]

S19 Table. MR-PRESSO global test, outlier test and distortion test results: effects of fetal growth on birth outcomes (A) and maternal BP and glucose levels (B)

A. Effects of birth weight associated SNPs on birth outcomes

	Gestational days			Preterm birth			Birth weight			Birth length		
Birth weight (86)[a]	global *p*-val	number of outliers	distortion *p*-val	global *p*-val	number of outliers	distortion *p*-val	global *p*-val	number of outliers	distortion *p*-val	global *p*-val	number of outliers	distortion *p*-val
h1	0.095	0	NA	0.196	0	NA	0.034	1	0.886	0.108	0	NA
h2	0.522	0	NA	0.179	0	NA	<0.001	3	0.581	0.501	0	NA
h3	0.052	0	NA	0.512	0	NA	0.674	0	NA	0.8	0	NA

B. Effects of birth weight associated SNPs on maternal BP and glucose levels

	BP[b]			SBP[b]			DBP[b]			FPG[c]		
Birth weight (86)[a]	global *p*-val	number of outliers	distortion *p*-val	global *p*-val	number of outliers	distortion *p*-val	global *p*-val	number of outliers	distortion *p*-val	global *p*-val	number of outliers	distortion *p*-val
h1	0.567	0	NA	0.779	0	NA	0.071	0	NA	0.008	1	0.265
h2	0.996	0	NA	0.991	0	NA	0.936	0	NA	<0.001	1	0.228
h3	0.873	0	NA	0.908	0	NA	0.438	0	NA	0.929	NA	NA

This table shows the results of MR-PRESSO global test, number of MR-PRESSO outliers and MR-PRESSO distortion test of the MR-PRESSO analyses of the effects of fetal growth on birth outcomes (A) and maternal BP and glucose levels (B) (S17 Table).

a: The number in the parenthesis is the numbers of SNPs used in the MR-PRESSO analyses. It can be shown that although global horizontal pleiotropy was detected in some of the MR analyses, the numbers of variants with significant horizontal pleiotropic effect were very small and none of the causal effects had significant distortions due to the horizontal pleiotropic outlier variants.

b: Maternal blood pressures (SBP: systolic blood pressure, DBP: diastolic blood pressure and BP: average of SBP and DBP) in ALSPAC and HAPO.

c: Fasting plasma glucose (FPG) during pregnancy measured in HAPO.
