# Supplementary material for: Dissecting maternal and fetal genetic effects underlying the associations between maternal phenotypes, birth outcomes, and adult phenotypes: A mendelian-randomization and haplotype-based genetic score analysis in 10,734 mother–infant pairs
Source: PLoS Med. 2020 Aug 25;17(8):e1003305. doi: 10.1371/journal.pmed.1003305 (PMC7447062; doi:10.1371/journal.pmed.1003305)
Supplement: S1 Fig — (PDF) [file pmed.1003305.s023.pdf]

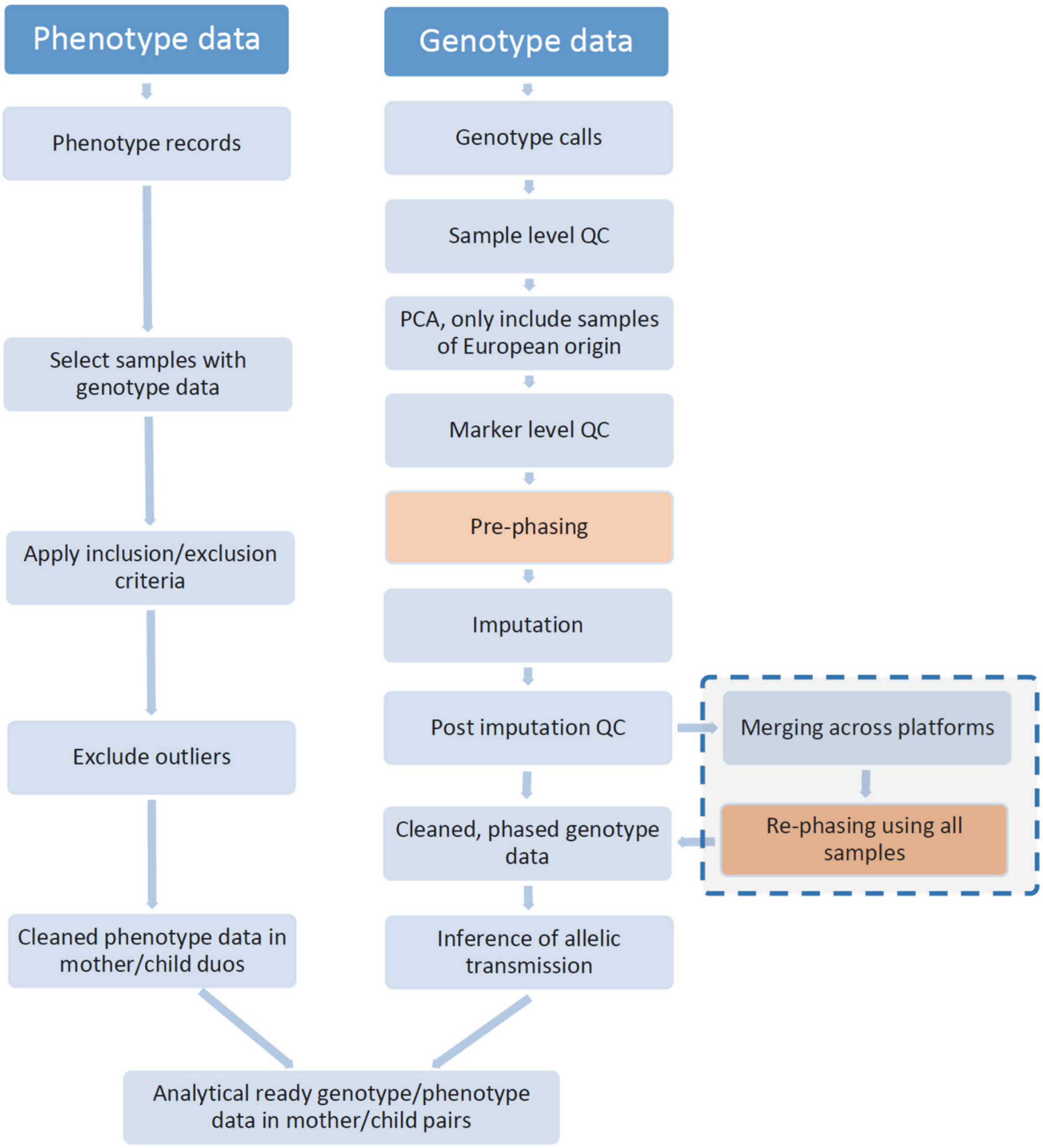

S1 Fig. Processing of phenotype and genotype data

The processing of phenotype (left) and genotype data (right). Haplotype transmission between mothers and their infants was obtained from the haplotypes inferred from the pre-phasing or re-phasing steps (highlighted in orange). For the FIN data set, a merging and a re-phasing step was added to merge and phase the imputed genotypes of mothers and infants genotyped by different platforms (dashed box). The sample-level QC (quality control) includes checking on call rate, overall heterogeneity, sex discrepancies and pedigree relationships. The marker-level QC includes removing markers with low call rate (<98%), low minor allele frequency (<0.01) or significant deviation from Hardy-Weinberg Equilibrium ($p < 5\times10^{-6}$). SNPs with low imputation quality (Rsq < 0.6) were also excluded (post imputation QC).
