# Supplementary material for: Dissecting maternal and fetal genetic effects underlying the associations between maternal phenotypes, birth outcomes, and adult phenotypes: A mendelian-randomization and haplotype-based genetic score analysis in 10,734 mother–infant pairs
Source: PLoS Med. 2020 Aug 25;17(8):e1003305. doi: 10.1371/journal.pmed.1003305 (PMC7447062; doi:10.1371/journal.pmed.1003305)
Supplement: S2 Fig — (PDF) [file pmed.1003305.s024.pdf]

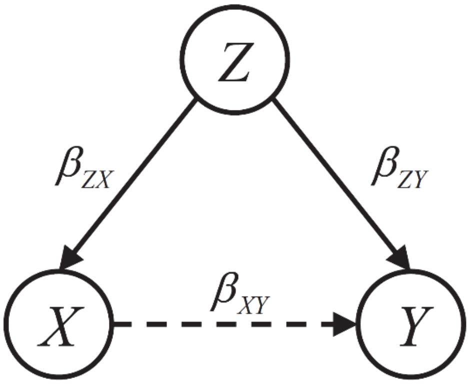

S2 Fig. General representation of confounded association

Spurious association between *X* and *Y* (β_{XY}) due to a confounder *Z*, which is causally associated with both *X* and *Y* with effect size β_{ZX} and β_{ZY}, respectively.
