# Supplementary material for: Dissecting maternal and fetal genetic effects underlying the associations between maternal phenotypes, birth outcomes, and adult phenotypes: A mendelian-randomization and haplotype-based genetic score analysis in 10,734 mother–infant pairs
Source: PLoS Med. 2020 Aug 25;17(8):e1003305. doi: 10.1371/journal.pmed.1003305 (PMC7447062; doi:10.1371/journal.pmed.1003305)
Supplement: S3 Fig — Genetically confounded association between a maternal phenotype and a birth outcome (A) and between a birth outcome and an adult phenotype in offspring (B) because of shared genetics. (PDF) [file pmed.1003305.s025.pdf]

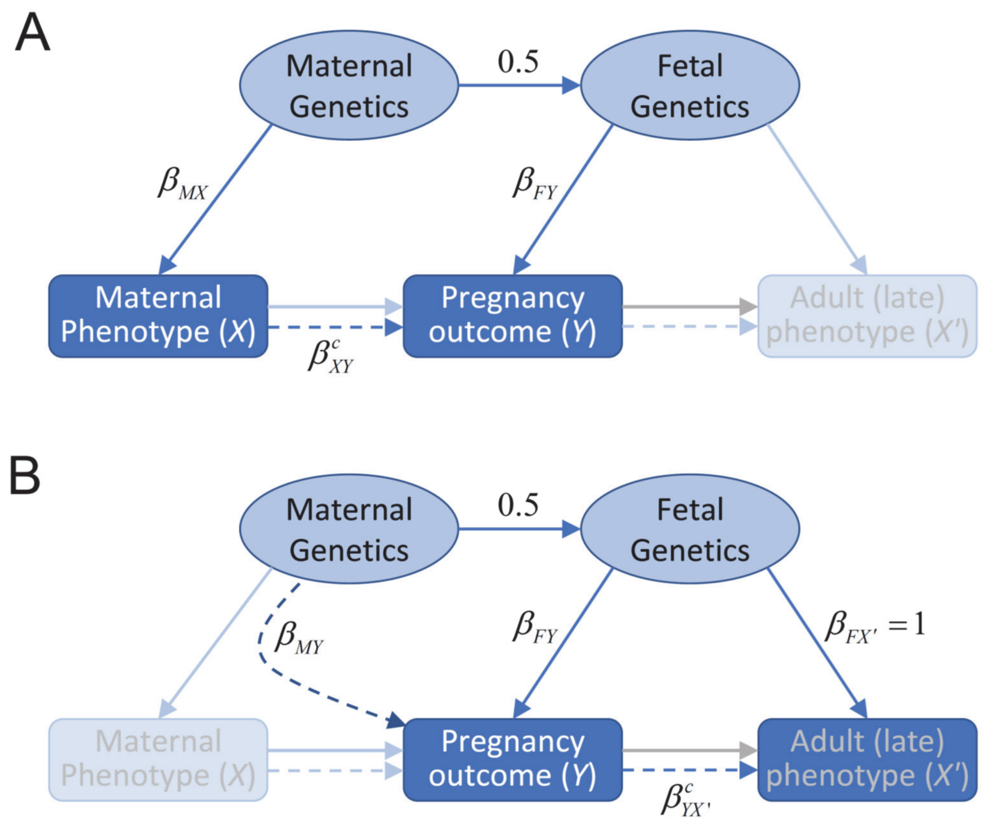

S3 Fig. Genetically confounded association between a maternal phenotype and a birth outcome (A) and between a birth outcome and an adult phenotype in offspring (B) because of shared genetics
