# Supplementary material for: Dissecting maternal and fetal genetic effects underlying the associations between maternal phenotypes, birth outcomes, and adult phenotypes: A mendelian-randomization and haplotype-based genetic score analysis in 10,734 mother–infant pairs
Source: PLoS Med. 2020 Aug 25;17(8):e1003305. doi: 10.1371/journal.pmed.1003305 (PMC7447062; doi:10.1371/journal.pmed.1003305)
Supplement: S4 Fig — MR, mendelian randomization. (PDF) [file pmed.1003305.s026.pdf]

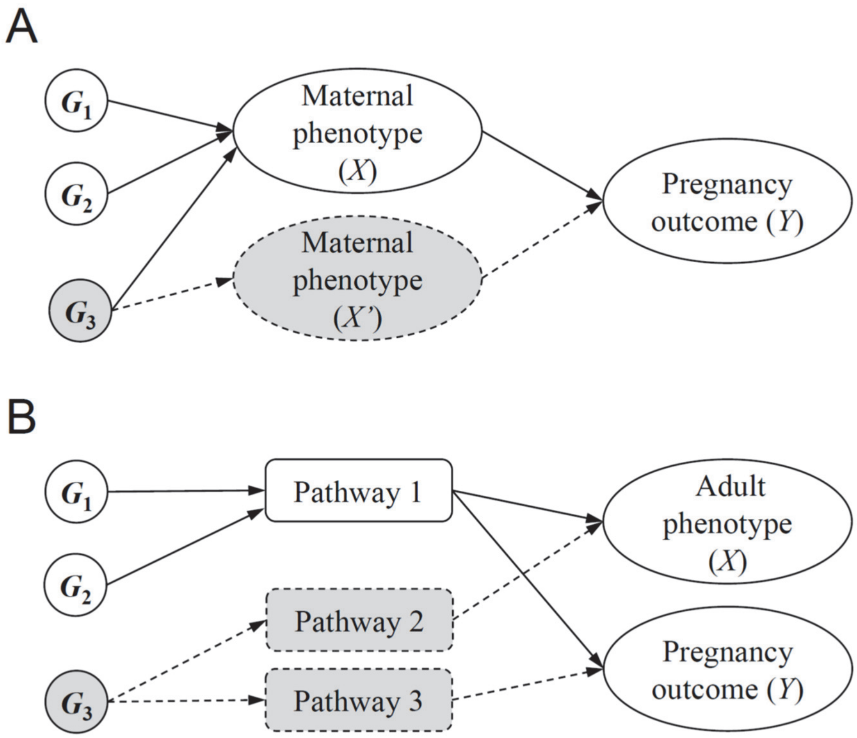

S4 Fig. Multivariable MR analysis of maternal and fetal genetic effect

A. Maternal effect. A list of genetic variants (G_1, G_2, G_3, …) are primarily associated with a maternal phenotype *X*, which causally influences a pregnancy outcome *Y*. In this case, these genetic variants can be used to instrument the maternal causal effect of *X* on *Y* and the estimated effect sizes of these variants on *Y*, especially those based on the non-transmitted alleles (the transmitted alleles can have fetal genetic effect), should be generally proportional to their effects on the maternal phenotype, which can be obtained from existing large GWA studies. Some variants (e.g. G_3) may associate with other maternal phenotype (*X'*), which also can influence *Y* (horizontal pleiotropy). Thus, the effects of these variants on *Y* are less correlated with their reported effects on *X*, which could be detected as outliers by MR-PRESSO. The situation of "fetal drive" (a fetal phenotype causally influences a maternal phenotype) is similar, in which case the paternal transmitted alleles of the SNPs associated with a fetal phenotype (e.g. birth weight) could be associated with a maternal phenotype (e.g. maternal blood pressure during pregnancy). **B. Fetal genetic effect.** Genetic variants (G_1, G_2, G_3, …) are associated with both an adult phenotype *X* and a pregnancy outcome *Y*. Some of the variants (e.g. G_1 and G_2) may be associated with both *X* and *Y* through a common pathway 1. Therefore, the estimated effects of these variants on *Y* (especially based on the paternal transmitted alleles to avoid maternal effect) should be proportional to their effects on *X* because these variants act through the same pathway, although there is no real causal relationship between *X* and *Y*. Some of the variants (e.g. G_3) may be associated with *X* and *Y* through distinct pathways (2 and 3) and therefore their effects on *X* and *Y* are relatively independent and could be detected as outliers with horizontal pleiotropy by MR-PRESSO.
