# Supplementary figures and images for: Dissecting maternal and fetal genetic effects underlying the associations between maternal phenotypes, birth outcomes, and adult phenotypes: A mendelian-randomization and haplotype-based genetic score analysis in 10,734 mother–infant pairs
Source: PLoS Med. 2020 Aug 25;17(8):e1003305. doi: 10.1371/journal.pmed.1003305 (PMC7447062; doi:10.1371/journal.pmed.1003305)

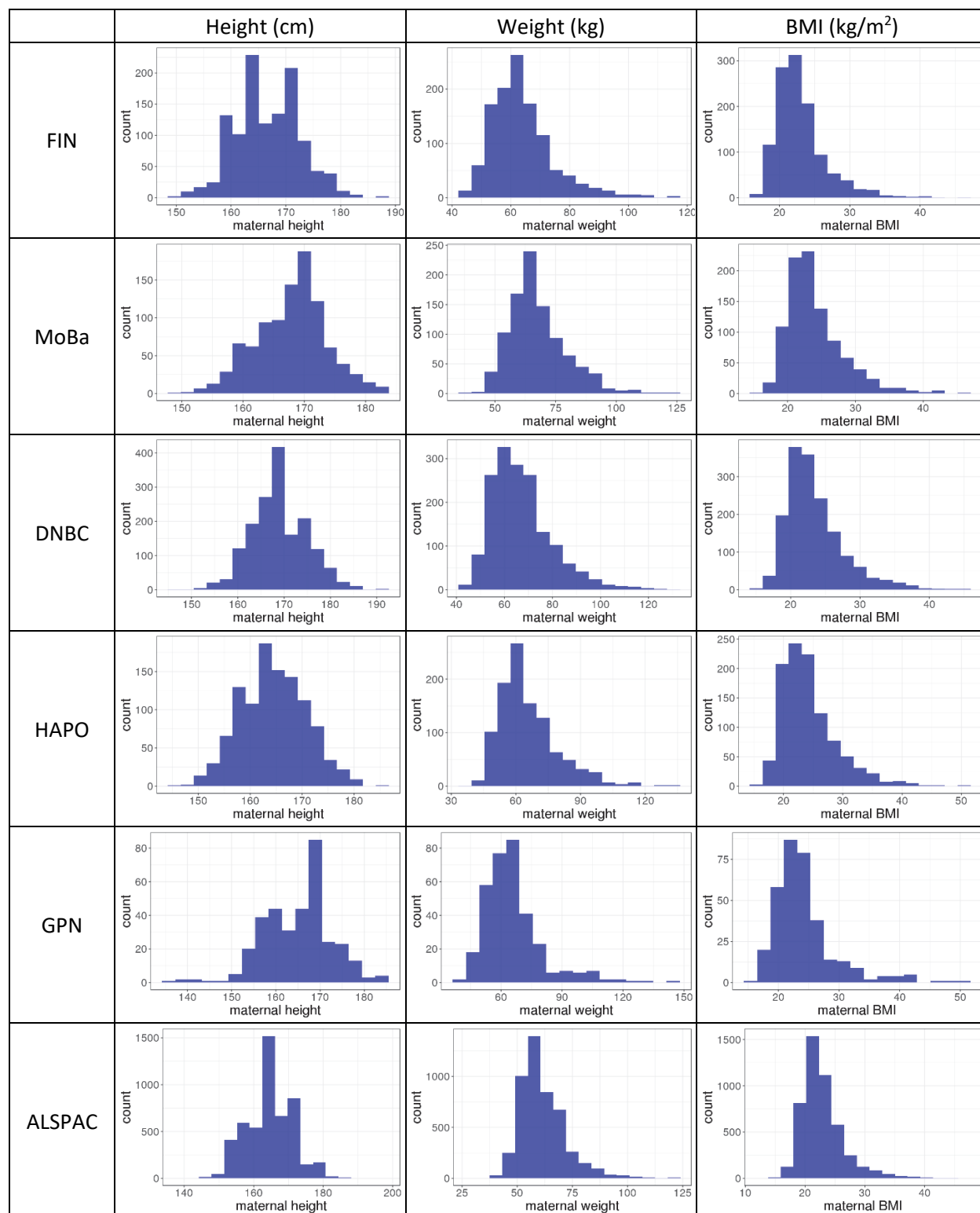

**S5 Fig. Distributions of maternal height, weight, and BMI**

Supplement: S5 Fig — BMI, body mass index. (PDF) [file pmed.1003305.s027.pdf]
