# Supplementary material for: Dissecting maternal and fetal genetic effects underlying the associations between maternal phenotypes, birth outcomes, and adult phenotypes: A mendelian-randomization and haplotype-based genetic score analysis in 10,734 mother–infant pairs
Source: PLoS Med. 2020 Aug 25;17(8):e1003305. doi: 10.1371/journal.pmed.1003305 (PMC7447062; doi:10.1371/journal.pmed.1003305)
Supplement: S6 Fig — BP, blood pressure. (PDF) [file pmed.1003305.s028.pdf]

	SBP (mmHg)	DBP (mmHg)	FPG* (mmol/L)
HAPO	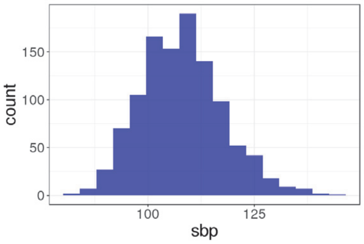	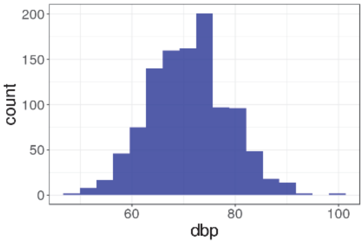	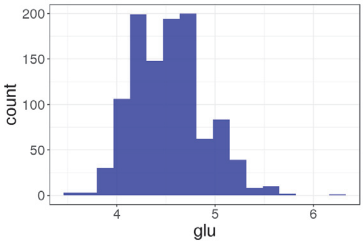
ALSPAC	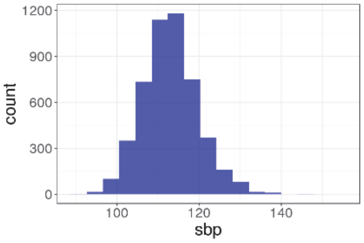	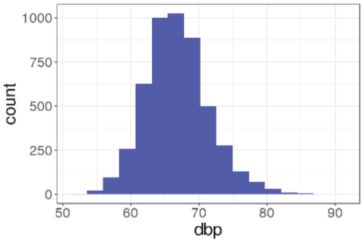	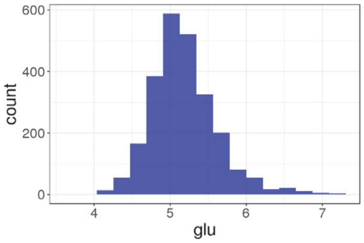

S6 Fig. Distributions of maternal BP and glucose levels

* In the HAPO study, fasting plasma glucose (FPG) in the mothers were measured between 24 and 32 weeks gestation. In the ALSPAC study, FPG was measured in a follow-up data collection 18 years after the pregnancy.
