# Supplementary material for: Dissecting maternal and fetal genetic effects underlying the associations between maternal phenotypes, birth outcomes, and adult phenotypes: A mendelian-randomization and haplotype-based genetic score analysis in 10,734 mother–infant pairs
Source: PLoS Med. 2020 Aug 25;17(8):e1003305. doi: 10.1371/journal.pmed.1003305 (PMC7447062; doi:10.1371/journal.pmed.1003305)
Supplement: S7 Fig — (PDF) [file pmed.1003305.s029.pdf]

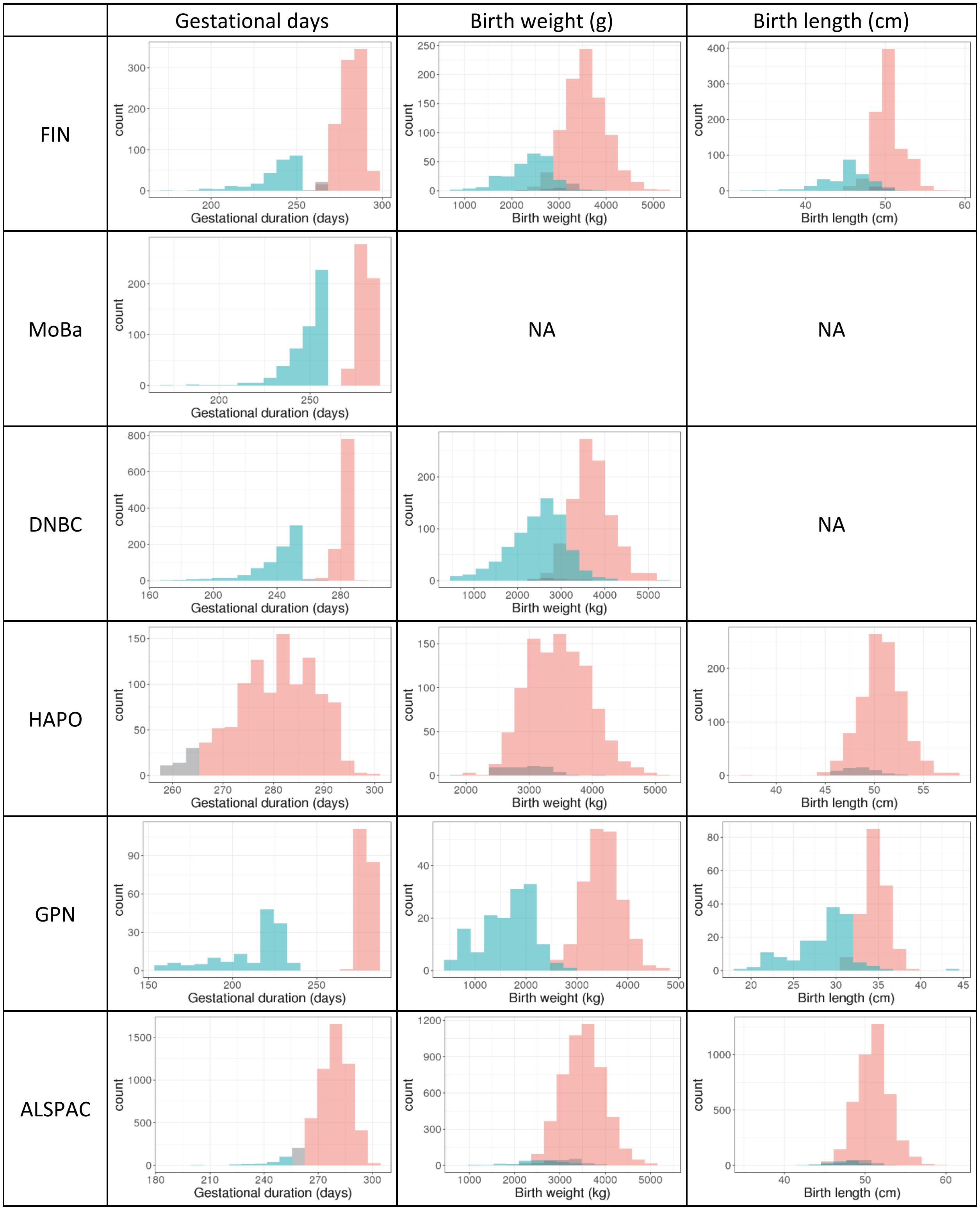

S7 Fig. Distributions of gestational days, birth weight, and birth length

The FIN, MoBa, DNBC, and the GPN were collected for case/control study of preterm birth; therefore, the gestational duration was truncated and enriched for cases (gestational duration < 37 weeks)
