# Supplementary material for: Dissecting maternal and fetal genetic effects underlying the associations between maternal phenotypes, birth outcomes, and adult phenotypes: A mendelian-randomization and haplotype-based genetic score analysis in 10,734 mother–infant pairs
Source: PLoS Med. 2020 Aug 25;17(8):e1003305. doi: 10.1371/journal.pmed.1003305 (PMC7447062; doi:10.1371/journal.pmed.1003305)
Supplement: S8 Fig — (PDF) [file pmed.1003305.s030.pdf]

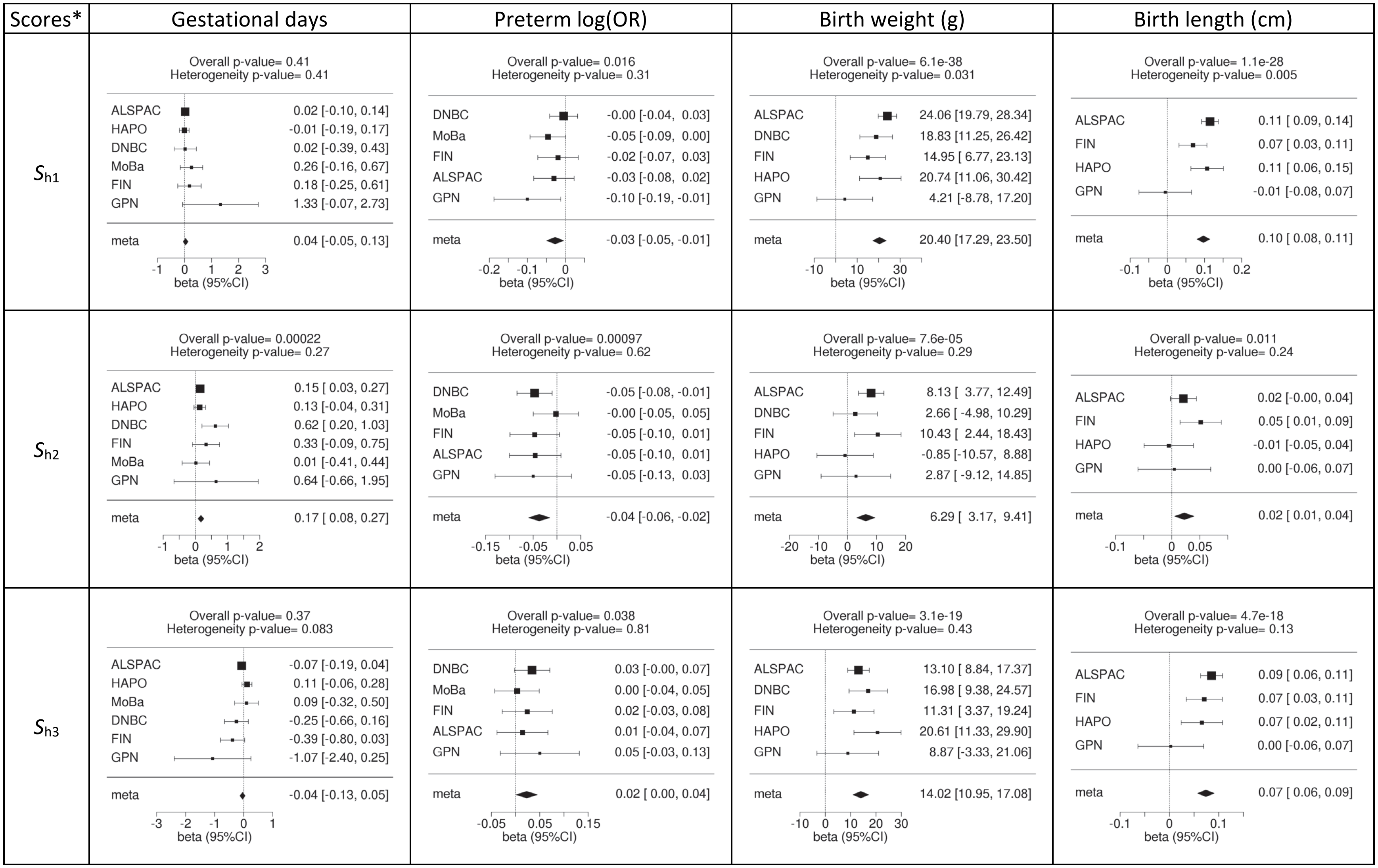

S8 Fig. Estimated effect sizes of adult height haplotype scores on pregnancy outcomes

* S_{h1}, S_{h2} and S_{h3} are height haplotype genetic scores built on maternal transmitted, maternal non-transmitted and paternal transmitted alleles.
