# Supplementary material for: Dissecting maternal and fetal genetic effects underlying the associations between maternal phenotypes, birth outcomes, and adult phenotypes: A mendelian-randomization and haplotype-based genetic score analysis in 10,734 mother–infant pairs
Source: PLoS Med. 2020 Aug 25;17(8):e1003305. doi: 10.1371/journal.pmed.1003305 (PMC7447062; doi:10.1371/journal.pmed.1003305)
Supplement: S13 Fig — SD, standard deviation. (PDF) [file pmed.1003305.s035.pdf]

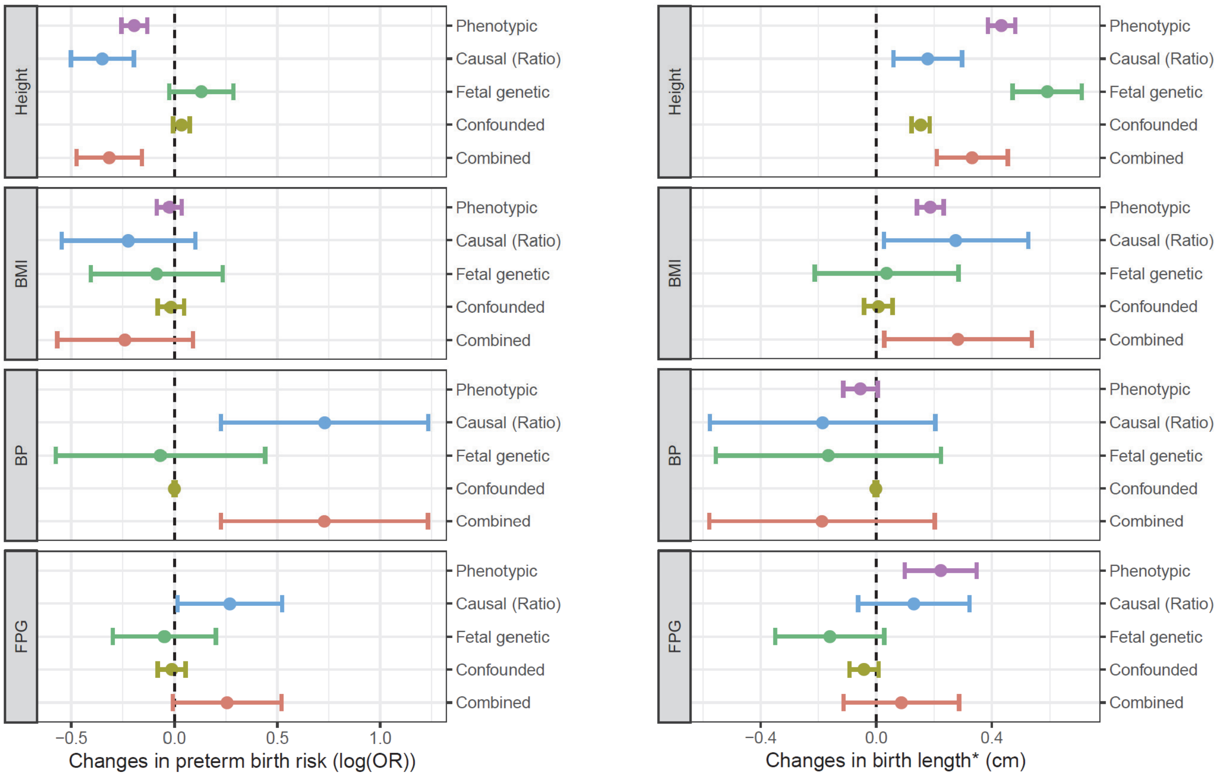

S13 Fig. Estimated sizes of causal effects and genetically confounded associations per 1-SD changes in maternal traits on preterm birth risk and birth length

The 1-SD values for maternal traits are: 6.4cm (height), 4.0kg/m^2 (BMI), 5.8mmHg (BP) and 0.36mmol/L (FPG). BMI, body mass index; BP, blood pressure; FPG, fasting plasma glucose; SD, standard deviation.
