# Supplementary material for: Dissecting maternal and fetal genetic effects underlying the associations between maternal phenotypes, birth outcomes, and adult phenotypes: A mendelian-randomization and haplotype-based genetic score analysis in 10,734 mother–infant pairs
Source: PLoS Med. 2020 Aug 25;17(8):e1003305. doi: 10.1371/journal.pmed.1003305 (PMC7447062; doi:10.1371/journal.pmed.1003305)
Supplement: S14 Fig — Estimated differences in adult phenotypes (SD) per 1-SD difference in gestational days (left) and preterm birth risk (right). SD, standard deviation. (PDF) [file pmed.1003305.s036.pdf]

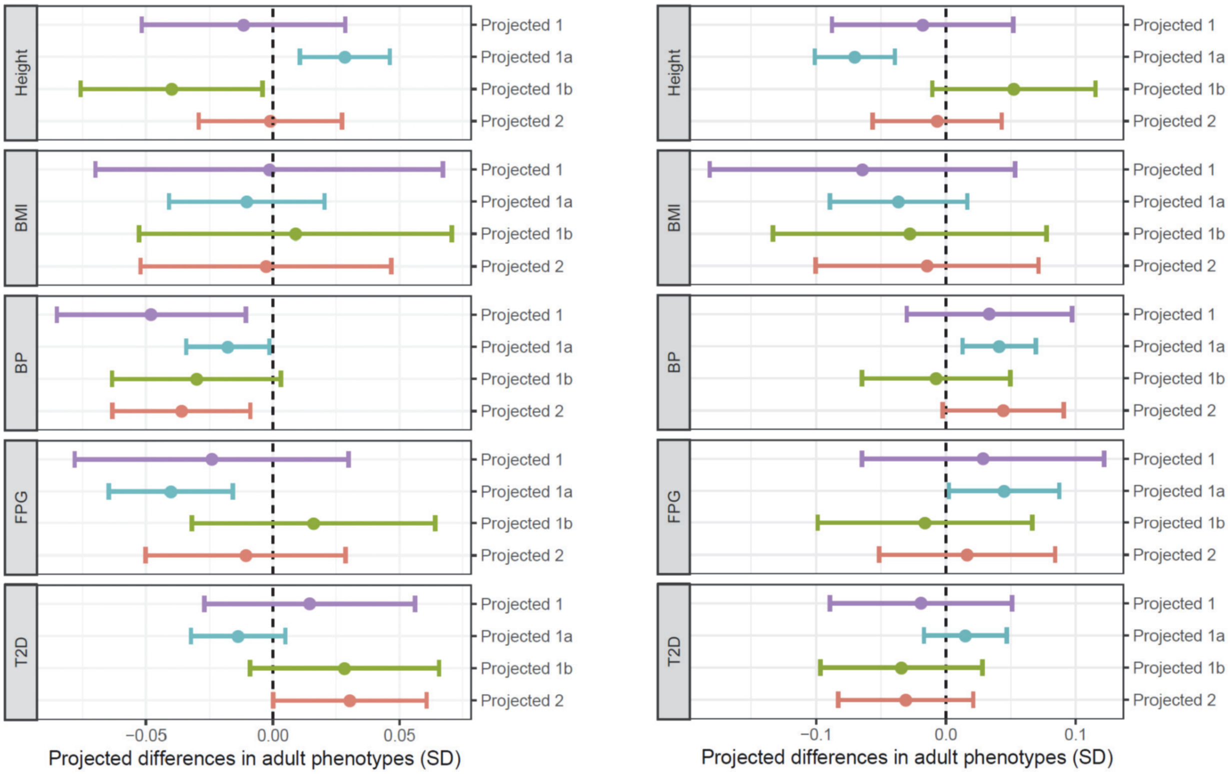

S14 Fig. Estimated differences in adult phenotypes (SD) per 1-SD difference in gestational days (left) and preterm birth risk (right)

The genetically confounded association was estimated by two methods (S1 Text):

$$\hat{\beta}^{c}_{YX'} = h^2 \frac{\mathrm{Var}(X')}{\mathrm{Var}(Y)}\left(\frac{\hat{\beta}_{MY}}{2} + \hat{\beta}_{FY}\right) \text{ (Method 1) and } \hat{\beta}^{c}_{YX'} = h^2 \frac{\mathrm{Var}(X')}{\mathrm{Var}(Y)}\left(\frac{\widehat{\beta_{h1}+\beta_{h3}}}{2}\right) \text{ (Method 2)}$$

Method 1a and Method 1b show the the maternal ($\frac{\beta_{MY}}{2}$) and the fetal component (β_{FY}) of the estimated genetically confounded associations.

1 SD of gestational duration was 11.4 days. The 1-SD values for adult phenotypes were assumed to be: 6.4cm (height), 4.0kg/m^2 (BMI), 6.9mmHg (BP), 0.37mmol/L (FPG) and 1.81 for log odds ratio of T2D. BMI, body mass index; BP, blood pressure; FPG, fasting plasma glucose; T2D, type 2 diabetes; SD, standard deviation.
