# Supplementary material for: Dissecting maternal and fetal genetic effects underlying the associations between maternal phenotypes, birth outcomes, and adult phenotypes: A mendelian-randomization and haplotype-based genetic score analysis in 10,734 mother–infant pairs
Source: PLoS Med. 2020 Aug 25;17(8):e1003305. doi: 10.1371/journal.pmed.1003305 (PMC7447062; doi:10.1371/journal.pmed.1003305)
Supplement: S18 Fig — MR, mendelian randomization. (PDF) [file pmed.1003305.s040.pdf]

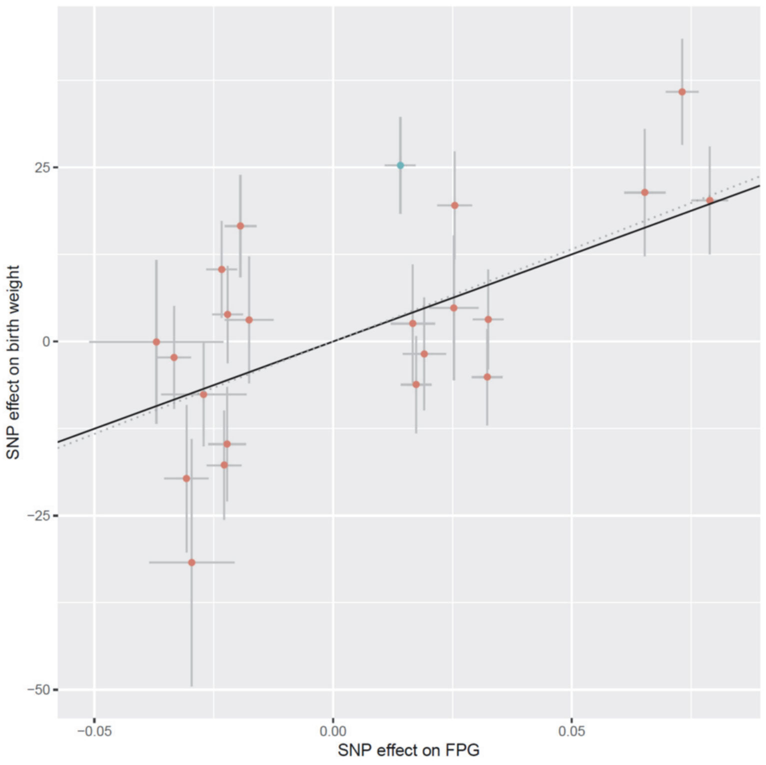

S18 Fig. An example of multivariable MR estimate

Multivariable MR estimate of causal effect of maternal fasting plasma glucose (FPG) on birth weight using maternal non-transmitted alleles. Each point represents the per allele association of the 22 FPG associated SNPs – x-axis: effect sizes on FPG level (and standard error) reported by the reference GWA study; y-axis: effect sizes on birth weight estimated from the maternal non-transmitted alleles (h2) from this study. The line shows the multivariable MR estimate. Removal of the outlier SNP (blue) did not change the causal estimate (the regression line).
