# Supplementary material for: Dissecting maternal and fetal genetic effects underlying the associations between maternal phenotypes, birth outcomes, and adult phenotypes: A mendelian-randomization and haplotype-based genetic score analysis in 10,734 mother–infant pairs
Source: PLoS Med. 2020 Aug 25;17(8):e1003305. doi: 10.1371/journal.pmed.1003305 (PMC7447062; doi:10.1371/journal.pmed.1003305)
Supplement: Attachment — Submitted filename: response.docx [file pmed.1003305.s041.docx]

# Requests from the editors:

To the data statement, we suggest adding a note to the effect that all data derived from the present study are presented with or in the paper (assuming this is the case).

We added “all data derived from the present study are presented with or in the paper”.

Please substitute a non-declarative title, and add a study descriptor (e.g., "...: a mendelian randomization study").

We changed the title to:

“*Dissecting maternal and fetal genetic effects underlying the associations between maternal phenotypes, birth outcomes and adult phenotypes – a haplotype-based genetic score analysis in mother/child pairs*”

Please convert your abstract into a three-section structure (background/methods and findings/conclusions).

Revised as suggested.

Please include summary demographic information for study participants in your abstract.

We added demographic information for the study participants.

We ask you to quote illustrative quantitative elements of your findings in the abstract to support the conclusions drawn.

We added quantitative results in the findings in the new abstract.

The final sentence of the "methods and findings" subsection of the abstract should summarize the study's main limitations.

We added the major limitations of the study at end of the "methods and findings".

Please present conclusions in the general form "In this study, we found that ... " or similar.

Changed as suggested.

After the abstract, we will need to ask you to add a new and accessible "author summary" section in non-identical prose. You may find it helpful to consult one or two recent research papers published in PLOS Medicine to get a sense of the preferred style.

We prepared “author summary” as requested.

Early in the methods section of your main text, please state whether the study had a protocol or prespecified analysis plan, and if so add the relevant document(s) as a supplementary file, referred to in the text. Please highlight analyses that were not prespecified.

A prospective protocol for analysis was not prepared for this study; however, the assembly of the data sets and all the analyses were planned in advance of data analysis.

Please add a sentence to your methods section to the effect that specific ethics approval was not required for the present study (assuming this is the case).

We added the ethics declaration. “This study involves re-analysis of existing data sets and the proposed analytical aims are consistent with the original consent agreements, under which the genomic and phenotypic data were obtained. Therefore, additional ethics approval was not required.”

Where available, please quote p values alongside 95% CI throughout the paper.

We added p values alongside with 95% CI throughout the paper.

Please quote exact p values or p<0.001, unless there are specific reasons to quote smaller p values.

We intend to keep the actual exact P values. We understand that expressing P values to more than 3 significant digits usually does not add useful information because P values with extreme results are sensitive to biases or departures from the assumptions of the test. However, in this manuscript, exact P values were used to compare the relative magnitude of the effects of the three haplotype scores tested using the same statistical model.

Throughout the text, please adapt reference callouts to the following format: "... cardiovascular diseases [11,12].".

We reformatted the references according to the PLOS Medicine style.

In your reference list, please ensure that journal names are abbreviated consistently, e.g., "J Pediatr." for reference 4 and "JAMA" for reference 9.

We fixed this problem.

Please spell out the group name in reference 5.

Fixed.

Please add full access details for references 31 & 40.

Fixed.

Please add a completed checklist for the most appropriate reporting guideline, which we suspect will be STROBE, as a supplementary file (referred to in your methods section). In the checklist, individual items should be referred to by section (e.g., "Methods") and paragraph number rather than by line or page numbers, as the latter generally change in the event of publication.

We added the STROBE Checklist for cross-sectional studies.

# Comments from the reviewers:

## *** Reviewer #1:

The authors present a very interesting study on quantifying the causal effects of maternal and fetal traits on birth outcomes using genetic scores constructed haplotype genetic scores which combines the maternal and fetal effects as a single unit. Using a large number of mother/infant duos singleton of European ancestry - they were able to identify the causal effects of maternal height, pre-pregnancy BMI, blood pressure and blood glucose on pregnancy outcomes (gestational duration), birth weight and birth length. What the authors have shown is mechanistic insight into the observed association between maternal phenotypes and birth outcomes which has implications for life-course association birth outcomes and future adult outcomes.

The results in itself are not particularly surprising that there are observed causal influences of maternal phenotypes on birth outcomes as this has been shown in many previous studies, including the authors own previous work but what is the novelty on this paper is actually the methodology itself. The authors developed a method which explicitly models maternal and fetal genetic effects using as a single analytic unit using instrumental variables of haplotype genetic scores. This means that a key advantage of this method is that they could explore whether any effects on fetal outcomes are associated gestational duration and whether fetal growth has an effect on gestational duration as well as maternal phenotypes in pregnancy. This is a complex analysis but presented in a way that is intuitive and generally easy to follow. The statistical methods underpinning all the analysis are a series of linear regression models, which have additive in nature, and then overall results are pooled across cohorts using meta-analyses

We appreciate the reviewer’s positive view of our study.

We agree with the reviewer that some of the associations between maternal phenotypes, birth outcomes and adult phenotypes have been shown in previous studies. Causal relations of some of these associations also have been investigated before using Mendelian randomization approach. In this current study we utilized a novel haplotype-based method and more powerful genetic instruments built on multiple variants reported by most recent large-scale GWA studies. As appreciated by the reviewer, one major innovation of this study is our new method to model maternal and fetal genetic effects using haplotype genetic scores, which enabled us to study a list of novel questions that have not been investigated before:

1. Our study extended beyond testing maternal causal effects to also including evaluating the genetically confounded association between maternal phenotypes and birth outcomes due to genetic sharing.
2. We also investigated the genetically confounded associations between birth outcomes and adult phenotypes in offspring.
3. We studied causal effect of fetal growth on maternal physiological conditions during pregnancy (i.e. “fetal drive”) using genetic scores built on paternal transmitted alleles.

There are few comments I have which the authors could find useful:

We address these comments one-by-one and for easy reference, we added number to the comments.

1. Abstract - Suggest conforming to PLOS Medicine style of three sections - background/methods&findings/conclusion. The methods are substantial so it would be useful to in the abstract to summarise the methodology and study design

We rewrote the Abstract according to the PLOS Medicine style. We provided more information about our methodology and results.

2. The study is rationale is strong and extends previous work by others and the authors themselves in determining causal associations between maternal phenotypes and pregnancy outcomes and adult cardio metabolic diseases. There is a good body of evidence already that the effects of maternal characteristics on fetal outcomes. The authors extend this body of evidence by combing maternal/fetal as a single unit to model genetic scores using a haplotype genetic score as an instrumental variable. Figure 2 is useful, the models assumes paternal non-transmitted alleles h4 (which isn't described in the legend) has no bearing on the maternal or fetal affects. This seems like a logical assumption but is there any evidence to suggest that paternal non-transmitted phenotypes may influence maternal phenotypes?

This is an interesting question. We think that the paternal non-transmitted alleles should be able to influence maternal phenotypes (during or beyond pregnancy), as the paternal genetics might influence the mothers through the environment that the fathers create for their couples. A similar phenomenon (genetic nurture) has been shown by Kong et al (Science 2018; 359:424-8), which suggested the paternal non-transmitted alleles could influence their children’s educational attainment and health-related traits. However, compared with the effect of parental educational attainment on their children’s education and health, the traits analyzed in our study (height, BMI, BP, FPG, T2D and birth weight) are less likely to have an important role in shaping a living environment that can influence their couples. Therefore, in this study we assumed the paternal variants associated with these traits (Table S4) can only influence birth outcomes or maternal pregnancy phenotype if they are transmitted to the fetuses (h3) – i.e. through fetal genetic effect (on birth outcomes) or “fetal drive” (on maternal pregnancy phenotypes).

3. Methods - ALSPAC blood pressure measurements: authors used BP measurements between 30-36 weeks - was this an average or a single point estimate?

It was an average of multiple blood pressure measurements between 30~36 weeks extracted from clinical records (Macdonald-Wallis, J Hypertens. 2011; 29:1703, Ref 35 in the paper). The individual measurements and the exact gestational weeks when these measurements were obtained were not available to this study.

4. Methods - PCA analysis to exclude non-European ancestry - how many were excluded in the end from non-European ancestry and how did this map up to what was self-reported as ethnicity?

We used phenotype and genotype data of 10,734 mother/infant pairs from six birth studies. These include three case/control data sets collected from Nordic countries (FIN, MOBA and DNBC) for genetic studies of preterm birth, a longitudinal birth cohort (ALSPAC) from the UK, the Hyperglycemia and Adverse Pregnancy Outcome (HAPO) Study and the Genomic and Proteomic Network for Preterm Birth Research (GPN) study.

The samples of the FIN, MOBA and DNBC were self-reported Europeans.

HAPO is a multicenter, international study, we only included the samples of European ancestry (Toronto, Canada, Belfast, UK, Brisbane and Newcastle, Australia).

The genotype data of ALSPAC were obtained from University of Bristol and all individuals with non-European ancestry were removed as part of their QC procedure.

For all these European data sets, we identified 211 subjects with non-European ancestry (Please see table below).

GPN is the only data set with diverse racial and ethnic background. More than one third of the pregnancies had mother and/or father with self-reported non-European ancestry. Based on genetic ancestry, we identified 1439 subjects, including 691 mothers and 748 infants of 830 pregnancies with non-European ancestry. Among these pregnancies, 539 (65%) had at least a self-reported non-Caucasian mother or father; 291 (35%) had both Caucasian mother or father. After using other qc filters and phenotype-based exclusion criteria, 363 mother/child pairs were included in the final analysis. All of these included mother/child pairs had both Caucasian mother and father except for two pregnancies, where the self-reported races of the fathers were non-Caucasian.

| No | Data set | Number of samples  before PCA | Number of samples with  non-European ancestry |
| --- | --- | --- | --- |
| 1 | ALSPAC | 17803 | 0 |
| 2 | FIN | 2934 | 26 |
| 3 | MOBA | 3058 | 71 |
| 4 | DNBC | 3853 | 24 |
| 5 | HAPO | 2836 | 90 |
| 6 | GPN | 2799 | 1439 |

5. Methods - genotype data - From previous GWA (Table S4 provided) - it would appear that heritability of certain most of the traits investigated varies from high levels (height - H2 0.8) to low (birthweight H2 0.3). This makes logical sense as features such as birth weight are have strong environmental influences (i.e. smoking in pregnancy). Can the authors discuss how the variation in the GRS instruments may have on the overall results and how better instruments would explain higher levels of variation in the phenotypic traits.

We added a paragraph to discuss this limitation.

“*Multiple maternal and fetal phenotypes were examined in this study. The heritability (Table S4) and the variance explained by their corresponding genetic scores (Table S5) varied across these phenotypic traits, …… Nevertheless, as the major aim the study is to distinguish and to compare the relative contributions of maternal and fetal effects of the same trait, instead of making accurate estimations of these effects and comparisons between different traits, we believe we have provided enough evidence to support our major conclusions (Figure 6).*”

6. Methods - association tests between haplotype genetic scores and pregnancy outcomes. How were potential interactions between handled or identified between gene-gene or gene-environment?

This is a very good point. It is possible that various gene-gene or gene-environment interactions may influence pregnancy phenotypes. The interactions might even exist between mothers and fetus (e.g. the h1, h2 and h3 genetic scores). As the consideration and testing of these interactions will substantially increase the complexity of the data analyses, we did not study these topics in this current study.

We considered LD redundancy between the GWA SNPs, and we excluded SNPs if they were in substantial LD (*r*^2^ > 0.2) with other more significant SNPs. Genetic score only captures the additive effects of selected SNPs, however, should explain the majority of the effects conferred by these SNPs.

7. Methods - multivariate MR analysis - when combing the results from all datasets, given the heterogeneity in the datasets (observed in the distributions provided in supplemental) as well as significant heterogeneity identified in several of the meta-analyses by Cochran's Q (Supplemental Figures S8-S12), it would have made sense to use a random-effects model. At least this should be considered in meta-analyses where there is a large amount of heterogeneity. This does have implications on the overall findings, but it would test the robustness of the pooling process.

We agree that there are certain levels of heterogeneity in the datasets, especially the distributions of gestational duration (Figure S7) due to the case/control sampling in 4 data sets (FIN, MoBa, DNBC and GPN). However, as we included gestational duration as a covariate in most of our analysis, we do not anticipate this could cause major problems.

Among the 60 meta-analyses of the associations between haplotype genetic score and pregnancy outcomes (Figure S8 to Figure 12), there were only 5 showing significant Cochran's Q. Therefore, we do not think that heterogeneity is a general problem in our meta-analyses. It should be noted that 4 of the 5 meta-analyses showed significant heterogeneity all involved distinct pattern of associations with fetal growth measures in the GPN data set (Figure S8, top row and Figure S12, second row). The GPN data set included more early preterm pregnancies (20-34wks), which might enrich for genetic and environmental risk factors that can reduce both gestational duration and fetal growth. After excluding the GPN data set, the Cochran's Q was no longer significant, and the p-values of the meta-analyses became even more significant (Please see the figure next page, which shows the meta-analyses without GPN). Therefore, we believe that our results based on fixed-effect meta-analysis were robust.

As suggested by the reviewer, we also conducted random-effects meta-analyses using REML method. This method estimates the amount of heterogeneity and a random-effects model is then automatically fitted. The meta-analysis of associations between haplotype genetic scores and birth outcomes using random-effects model were appended in Supplementary Table S18 in this revision. It can be shown that the results were essentially the same as the meta-analysis using fixed-effect model (Table 1) except for the above-mentioned 4 exceptions. This again indicated that in general, heterogeneity is not significant problem and our fixed-effect analysis is appropriate.

|  | Birth weight (g) | Birth length (cm) |
| --- | --- | --- |
| Height, Sh1  (Figure S8  Top row) |  |  |
| T2D, Sh2  (Figure S12, Second row) |  |  |

8. Results - association between genetic score and maternal phenotypes. The genetic scores explain > 20% of the maternal height variance but the remaining genotypes explain less than 5% variance of the maternal phenotypes - suggesting strong environmental influences which is not surprising. This also suggests that perhaps in the haplotype model exploring casual effects using the genetic instruments to consider incorporating more environmental covariates (presumably some of the birth cohorts would have collected these data - for instance ALSPAC). Though genetic risk scores may not associated with many of the environmental demographic factors, they might have some association with potential other maternal phenotypes indicative of maternal cardio-metabolic health which may have not been considered

Yes. the genetic scores explained different fractions of phenotypic variance (Table S5). In addition to the strong environmental influences in some of the traits (e.g. blood pressure), the number, allele frequencies and effect sizes of the identified trait associated variants (GWA SNPs) are the primary factors that define the phenotypic variance explained by the genetic scores. Because of these different levels of phenotypic variance explained, our study had different power in testing the causal effects or genetic correlations among different traits. We discussed this limitation in more detail in this revision.

We agree with the reviewer that incorporating environmental covariates might be beneficial. However, due to the incompleteness and inconsistency in the environmental data across the different study sites, it was hard to include the same set of environmental or socioeconomic factors in the analysis. As suggested by the reviewer, we incorporated several important environmental and socioeconomic factors in individual data sets and the obtained similar results.

The genetic score for a specific trait was built on multiple GWA SNPs. It is likely that some of the SNPs might associated with other phenotypes due to biological pleiotropy. We used the MR-PRESSO method to detect and remove SNPs with horizontal pleiotropic effects. The results showed some evidence of pleiotropy effect. After excluding the outliers, the corrected estimates and the p-values did not change substantially, suggesting the genetic scores were most strongly associated with their corresponding phenotype and the results were robust to the “residual” pleiotropic effects of individual SNPs.

9. Results - one of the limiting factors which have not been considered is maternal nutrient intake during pregnancy, and certain behavioural factors (i.e. smoking, alcohol usage) which may have some link to genetic link towards to these behaviours. Could the authors comment whether these behavioural or nutrition factors could have a large influence on their findings.

We agree with the reviewer that many maternal nutritional or behavioral factors can influence pregnancy outcomes and many of these factors can also be influenced by maternal genetics. But we do not anticipate the genetic control of these nutritional or behavioral factors has large influence on our findings because there were no strong genetic correlations between these nutritional or behavioral factors and the maternal traits examined in this study (i.e. height, BMI, FPG and BP).

## *** Reviewer #2:

The proposed manuscript entitled "Haplotype genetic score analysis in 10,734 mother/infant pairs reveals complex maternal and fetal genetic effects underlying the associations between maternal phenotypes, birth outcomes and adult phenotypes" by Chen et al. seeks to dissect the maternal and fetal genetic effects and their complex relationship between maternal traits and offspring outcomes including gestational duration, birth weight, and birth length. They utilized haplotype scores and Mendelian randomization to identify that tall maternal stature and high maternal blood glucose casually increase birth size, in the neonate, height and blood pressure increasing alleles can lead to increased and decreased birth weight, alleles associated with increased birth weight reduce the time of gestation and increase maternal blood pressure. This is an interesting article with some intriguing results but there are several issues that first need to be addressed.

We thank the reviewer for the positive view of our study.

1)	The Abstract needs to be expanded on and fully elucidated for the journal and should include Background, Methods, and Findings and Conclusions. Also given the complexity of some of the underlying statistical genetics in the article it should include an Author Summary as well to better present this to a clinical audience.

We rewrote the Abstract according to the journal’s style. We included an Author Summary to present the major findings in a way that can be appreciated by clinical audience.

2)	The second paragraph in the introduction describes a lot of the recent work on the underlying maternal/fetal genetic relationship with birthweight but provides less information on the relationship between birthweight and some of the other fetal outcomes proposed here. This paragraph should be shortened and go into more detail regarding the latter as this is the major gap in the literature this paper is seeking to address.

As suggested by the reviewer, we cut some detailed description of the recent work on birth weight and highlighted the major gaps that the paper is seeking to address.

3)	Throughout the manuscript when you discuss blood pressure it is unclear how this is defined. Are you using systolic or diastolic blood pressure as both are included in the supplementary tables or are you using a combined phenotype?

Sorry for the confusion. In the analysis of SBP and DBP as a phenotype, we kept them as distinct phenotypes (Table S2, S3, S5 and S8). We also constructed genetic scores for both SBP and DBP using the 831 GWA SNPs and their reported effects on SBP and DBP, respectively. To simplify the presentation of the haplotype genetic association analyses (Table 1 and Table S10, 11, 12 and 14), we built a score for **average blood pressure (BP)** based on the average of the SBP and DBP scores. We described this in the supplementary method and made it clearer throughout the manuscript.

4)	The authors used the program MR-PRESSO to avoid horizontal pleiotropy as the correctly mention the high correlation between traits. However, they do not provide any measure of horizontal pleiotropy. As the authors of the program mention, it is only useful when horizontal pleiotropy is < 50%. This should be addressed in some way in the manuscript. This is not clearly shown in the supplementary tables either.

We thank the reviewer for this great suggestion. In our analysis, MR-PRESSO global test was used to detect the presence of horizontal pleiotropy, MR-PRESSO outlier test to identify outliers, and MR-PRESSO distortion test to estimate the distortion caused by significant horizontal pleiotropic outlier variants. As indicated by these test results, although global horizontal pleiotropy was detected in some of the MR analyses, the number of variants with significant horizontal pleiotropic effect in these analyses was very small, much less than 50%. As a result, none of the causal effects had significant distortions due to the horizontal pleiotropic outlier variants. For simplicity, these results were only briefly mentioned in our original manuscript. According to the reviewer’s suggestion, we presented the results of the MR-PRESSO global test, MR-PRESSO outlier tests and the MR-PRESSO distortion tests in Supplementary Table S16 and S17 (corresponding to the MR-PRESSO analyses shown in Table S14 and S15 respectively). We also added the description in the methods and results accordingly.

5)	For the meta-analysis was any specific computer program used and are the Q scores provided in any of the supplementary tables?

We used the R metafor package and we inserted reference for this program. The forest plots and p-values for the test of heterogeneity (but not the Q scores) of the meta-analyses of haplotype genetic score associations were showed in Supplementary Figure 8-12, 16 and 17. The p-values for the tests of heterogeneity of the meta-analyses of phenotype associations and the associations between maternal genetic scores and maternal traits were shown in Supplementary Table S3, S6 to S9.

6)	The inclusion of the fasting blood glucose in ALSPAC 18 years later seems spurious, as most blood chemistry would be expected to change over time and not sure how this would impact any fetal measurements. Horse has already left the barn in this case.

This is reasonable concern. As indicated in Table S3 the FPG level measured 18 years later in the ALSPAC showed different association patterns with birth outcomes from the FPG level in the HAPO study. The FPG measured during pregnancy in HAPO showed much stronger positive association with birth weight and length. The FPG genetic score built on the 22 SNPs associated with FPG identified in non-diabetic individuals also explained larger variance of FPG in HAPO. In our analyses of maternal causal effects, we used the ratio estimate as well as the TSLS (two-stage least squares) method and only the TSLS method requires exposure data (i.e. maternal FPG during pregnancy). Therefore, we only include HAPO in the TSLS estimation of causal effect of maternal FPG. The analysis of haplotype genetic score associations and the causal effect estimation using ratio method were not influenced by this problem.

7)	In the results, the fact that there is a smaller sample size (2 cohorts) it would appear unsurprising that the blood pressure is half, also given that there is a potential age discrepancy between the two and blood pressure is notoriously heterogeneous.

Yes. Blood pressure data during pregnancy were only available in ALSPAC and DNBC, which is a limitation of this current study. As pointed out by the reviewer, blood pressure can be influenced by many factors and could easily be heterogeneous between studies. However, we did not observe substantial difference in blood pressures between these two data sets (DNBC: SBP: 108±9.7; DBP: 71±7.8mmHg vs ALSPAC: SBP: 113±6.8; DBP: 67±4.7mmHg) (Table S2). The blood pressure genetic score also associated with SBP or DBP in these two data sets with similar strength. The only significant difference between the two data sets was observed in the associations between blood pressure and pregnancy outcomes (Table S3) – SBP and DBP were much more negatively associated with gestational duration and birth size in ALSPAC.

It should be noted that our analyses of haplotype genetic associations (Table 1) and ratio estimation of causal effect did not request the maternal phenotype (i.e. maternal blood pressure) and therefore were robust to incompleteness and heterogeneity in maternal phenotype data.

This incompleteness and heterogeneity in maternal phenotype data reduced the power in the analysis of “fetal drive”, in which these maternal phenotypes were dependent variables. We discussed this limitation in more detail in this revision.

“*In the analysis of causal effect of fetal growth on maternal blood pressure and FPG during pregnancy (Table 2B), the phenotype data were only available in a subset of samples, which comprised the power of statistical analyses. These results will require further replication in independent cohorts.”*

8)	How is pre-term birth being assessed throughout, there is no clear definition provided but the term is used throughout the results.

We thank the reviewer for pointing out this problem. We now provided clear definition of preterm birth (<37 weeks) in the method section.

9)	I would be very cautious regarding the fasting glucose results as this only comes from one cohort, so I am not sure there is strong evidence for the results here.

As we mentioned in our response to question 7, our analyses of haplotype genetic associations (Table 1) and estimation of causal effect by ratio estimate (Table S11) did not require the maternal phenotype because we used the genetic scores to instrument the phenotype.

We agree that in the analysis of fetal drive (Table 2), the lack of FPG and blood pressure during pregnancy in many of the data sets is a limitation (the phenotypes were used as the dependent variables in this analysis). In this revision, we discussed this problem as a limitation (please see our response to question 7).

10)	In the meta-analysis was any sensitivity testing done, such as drop one cohort out and see if they have more influence on the overall results.

We did not perform ‘leave-one-out’ sensitivity analyses in all the meta-analyses. Instead we examined the heterogeneity using Cochran’s Q test and carefully inspected the source of heterogeneity using forest plots (Figure S8 to S12). Among the 60 meta-analyses of the associations between haplotype genetic score and pregnancy outcomes (Figure S8 to Figure 12), there were only 5 show significant Cochran's Q and 4 involved distinct patterns of associations with fetal growth measures in the GPN data set (Figure S8, top row and Figure S12, second row). After excluding the GPN data set, the Q tests were no longer significant the results became even stronger. We also performed random-effects meta-analyses using REML method and the results were presented in Supplementary Table S18, which were essentially the same as the meta-analysis using fixed-effect model (Table 1). Please also see our response to reviewer 1’s question 7 for more detail.

## *** Reviewer #3:

This paper by Chen, Bacelis, et al. explores the contributions of maternal and fetal genetics, and maternal phenotypes, to pregnancy outcomes - a topic that has some significant general interest. Since I am not a statistical geneticist my critique is brief and based more on common sense than on technical considerations. Nonetheless, it might be useful since PLoS Medicine's target audience is broad.

We appreciate the reviewer’s comments relevant to the broad audience of PLOS Medicine.

This is an extremely complex piece of work, involving phenotypic (height, weight, metabolic, pregnancy duration, etc.) and genotypic (SNP) data of 10,734 mother/infant pairs from six independently conducted birth studies. Each of these birth studies had different designs and different ways of obtaining both the genotypic and phenotypic data - which necessitated several significant compromises in their joint analysis. To cite just one example, maternal fasting plasma glucose (FPG) levels during pregnancy were available only in the HAPO study, while FPG was measured in over 4000 ALSPAC mothers not during pregnancy but in a follow-up data collection 18 years after the pregnancy.

We agree with the reviewer that this is a complex piece of work. Also as indicated by the reviewer using FPG example, the heterogeneity and incomplete phenotype data across different data sets might introduce difficulties in the interpretation of results. The same concerns have been raised by reviewer 2. However, as we explained in our response to Reviewer 2’s questions 7 and 9, similar to two-sample Mendelian randomization, our major results of haplotype genetic score associations (Table 1), the estimation of maternal causal effect, fetal genetic effect using ratio estimates (Figure 3 and Table S11), and the projected association between birth outcomes and adult phenotypes (Figure 4 and 5) did not require maternal phenotype data and therefore will not be compromised by the missing or heterogeneity of maternal phenotype data.

However, the heterogeneity, incomplete phenotype data and the different instrumental strength do bring some problems in data analysis, including different power in testing the causal effects or genetic correlations for different traits and comprised power in the analysis of causal effect of fetal growth on maternal blood pressure and FPG during pregnancy. We discussed these limitations in this revision.

I believe the paper needs to be rewritten, and I have two suggestions:

1. The concepts of maternal and fetal "genotype genetic scores" and "haplotype genetic scores" will be new for a general readership and are important for understanding the study. These concepts should be illustrated diagrammatically in a main figure. The current diagrams with arrows indicating potential maternal-fetal interactions are useful and should be kept - but should simplified to deal with only the strongest and most reproducible findings (see #2 below).

We introduced the concept of “haplotype genetic scores" to study the casual effect of maternal phenotype on pregnancy outcomes in PLOS Medicine, 2015. This concept has been well accepted by the field and the method has been used in many high impact studies. The initial idea of using maternal non-transmitted haplotype to study the casual effect of maternal phenotype on pregnancy outcomes was illustrated diagrammatically in the original publication and several follow-up reviews (e.g. Zhang et al. Best Pract Res Clin Obstet Gynaecol. 2018; 52:33 and Lawlor et al. Wellcome Open Res. 2017; 2:11).

In this current study, we expanded our previous method to explicitly model maternal and fetal genetic effects using linear combinations of the haplotype genetic scores and estimate the genetic links (confounded associations due to shared genetics) between maternal phenotype, birth outcomes and adult phenotypes (Figure 1 and Figure 2). We agree that these are complicated problems and full understanding of our statistical approach will require some expertise in statistical genetics and certain familiarity with the problems. These complications do bring some challenges in the presentation of the results. However, as these problems are naturally interconnected, we intend to describe our findings in an integrative manner despite these challenges.

The major purpose of this study is to reveal the complex maternal and fetal genetic effects underlying the associations between maternal phenotypes, birth outcomes and adult phenotypes. Instead of focusing on pieces of strongest and most reproducible finding, we prefer presenting the “full picture” (Table 1, 2 and Figure 6) to convey the idea that all the maternal and fetal genetic effects are interconnected and jointly shape the observed associations between maternal phenotypes, birth outcomes and adult phenotypes. In addition, the multiple phenotype traits examined in this study are all related to growth and metabolism (e.g. height, BMI, BP, FPG and fetal growth), therefore, we believe showing the results in an integrative manner will facilitate the understanding of the relationships between these traits.

2. More crucially, what are the strongest and most reproducible findings in this study? Because of the way the authors chose to analyze and describe all 6 birth studies in parallel, it is difficult or impossible to extract a clear notion of replication of any of the specific findings across these independently conducted and differently designed birth studies. The authors should consider analyzing one of the birth studies (whichever one they see as most complete for their analytical approach and for the maternal and fetal phenotypes that they believe are most informative), and then vetting their strongest findings using the other 5 studies as replication sets. This approach would be easier to grasp and would have obvious advantages, both for distilling the most convincing results and for simplifying the tables, which are currently very difficult to read. The "study pooling" approach would not have to be totally discarded - it could be presented as supplemental data.

From this comprehensive study, we obtained multiple strong and significant findings. We now summarized the major findings at the beginning of the discussion section. We also re-drew Figure 6 by using different line widths to reflect the estimated effect sizes of the associations. We hope these revisions will improve the readability of our paper.

As mentioned in our response to the first question, we intent to present a “complete picture” of the maternal and fetal genetic effects of the multiple phenotypes examined in this study. We understand that our findings are associated with different levels of statistical evidence. We did not choose to present the strongest findings (e.g. based on p-values) for two major reasons:

*First*, in addition to the magnitude of the real effect, the statistical significance of a finding is largely influenced by data availability and instrumental strength of the genetic scores, which are different between different phenotypes. For example, the significance level of the maternal causal effect of maternal height on gestational duration (p=0.0017) is much stronger than the significance level of the maternal causal effect of blood pressure on gestational duration (p=0.03) (Table 1), but the estimated causal effect per sd change in blood pressure is actually larger than maternal height albeit with larger standard error (Table S11). This is because the genetic score for blood pressure explain much smaller phenotypic variance (2% vs 25% for height) and hence smaller instrumental strength. For this reason, it is hard to select “most significant findings” between different phenotypes based on statistical significance alone. However, for a particular phenotype, as we tested the three haplotype genetic scores simultaneously in a single regression model, the estimated effect sizes of these haplotypes scores and their associated p-values can be directly compared to assess the directions and relative contributions of the maternal and fetal effects. For example, for both phenotypes (height and blood pressure), we can draw the conclusion that the maternal effect appears to be more significant than the fetal genetic effect.

*Secondly*, we think that in order to understand the relative contributions of maternal and fetal effects and to obtain mechanistic implications, it is necessary to present both significant and non-significant results. For example, we showed a significant positive association between maternal non-transmitted FPG genetic score (*S*_h2_) and birth weight (P=4.7E-6) and a negative but non-significant association between paternal transmitted FPG genetic score and birth weight (P=0.38), which indicated a strong positive maternal causal effect of FPG on fetal growth. We also showed the T2D genetic scores associated with birth weight in a similar fashion, but with a less significant association with *S*_h2_ (P=0.0012) and a more significant association with *S*_h3_ (P=0.0061). By digesting these results together, we can draw a mechanistic interpretation that maternal FPG causally drives fetal growth while alleles associated with T2D risks other than normal control of blood glucose levels have negative impact on fetal growth through fetal genetic effect, which supports the fetal insulin hypothesis.

We appreciate the reviewer’s suggestion to split our date sets into discovery and replication sets. However, as our study was driven by well-established evidence of phenotypic associations between maternal traits and birth outcomes, and in turn with adult phenotypes, we intend to dissect and test their underlying maternal and fetal genetic effects with all available data sets instead of using a discovery/replication approach to discover the strongest and most reproducible findings. Also as discussed in our response to reviewer 1’s question 7, we did not observe substantial heterogeneity in our meta-analysis (except in four occasions in which the GPN data set was clearly the source of heterogeneity). Therefore, we believe that a meta-analysis of all the data sets together will provide better power than a discovery/replication approach. Also, we believe that presenting the meta-analysis of all the data sets together is more straightforward and fits our purpose (showing “whole picture” instead of identifying the strongest and most reproducible findings) better.

Still we tested the associations between haplotype genetic scores and birth outcomes in ALSPAC and in the other 5 data sets separately as suggested by the reviewer. The results were appended in Supplementary Table S19 (ALSPAC) and S20 (other five data sets). The results showed similar haplotype genetic score associations with birth outcomes except the five data sets had more associations with preterm birth probably because these data sets were enriched for preterm pregnancies.

## *** Reviewer #4:

Ge Zhang et al implemented a haplotype-based genetic score method to dissect the maternal causal effects and fetal genetic effects on pregnancy outcomes known to be relevant for life-course health and disease status. A key strength of this work is the depth of analyses done to partition the transmitted and non-transmitted parental haplotypes and model their relationships with maternal phenotypes and offspring outcomes. The approach has helped unravel some novel insights about complex relations of maternal, offspring and adult phenotypes, and validated known relationships from previous studies. This is a great scientific addition to the literature on early development and its relevance in adult cardiovascular diseases. Please find some comments below, most of which are minor.

We thank the reviewer for the positive evaluation of our manuscript.

1. The finding that the fetal growth raising genetic effect may negatively impact maternal BP during pregnancy is interesting. However, fetal growth rate was measured at birth whereas BP was measured over a range of prenatal gestation weeks, making the conclusion less strong. Perhaps, adjusting for the gestational week when BP was measured in ALSPAC and HAPO may be done to evaluate whether the effect remains stable.

This is a good point. But unfortunately, the blood pressure (SBP and DBP) in ALSPAC was an average of the blood pressure measured between 30 and 36 weeks and we do not have access to the individual BP measurements and the exact gestational weeks when the measurements were done. The blood pressure in HAPO was a single-time measurement obtained between 24 and 32 weeks and the SBP and DBP were not significantly associated with the gestational weeks when they were measured (although there was a trend of increasing blood pressure with gestational weeks). We included gestational weeks at measurement in the analysis of the HAPO data and it did not change the results.

2. The authors found a negative effect of the maternal non-transmitted haplotype BP score on birthweight and lengths. Testing the fetal causal effects using only the 64 “fetal-only” birthweight-associated SNPs reported in Warrington et al’s 2019 may further clarify the relative effects of Sh1, Sh2 and Sh3.

The negative association between the maternal non-transmitted haplotype BP score with birthweight and length indicated the negative causal effect of maternal BP. This observation is consistent with previous findings (Tyrrell et al. Jama 2016; 315:1129 and Warrington et al. Nat Genet 2019; 51:804). Our analysis also showed that both maternal transmitted and paternal transmitted BP scores were negatively associated with birth weight (Table 1), suggesting a negative fetal genetic effect of BP increasing alleles on birth weight. This is consistent with Horikoshi et al. (Nature 2016; 538:248), which showed negative genetic correlation between birth weight and blood pressure.

We currently used 86 SNPs (selected from the 106 SNPs with confirmed fetal genetic effects from Warrington’s et al, Supplementary Table S4) in constructing the birth weight genetic score. We used this inclusive set (some of them also have maternal effect) instead of the fetal-only” SNPs because our analysis of “fetal drive” based on the paternal transmitted alleles are robust to possible maternal effect.

As suggested by the reviewer, we tested the SNPs with only fetal effect reported by Warrington et al. From the 64 SNPs, we used 54 SNPs (excluding 3 SNPs due to LD redundancy and 7 SNPs not available in all data sets) and constructed genetic scores. This new fetal genetic score was significantly associated with gestational age adjusted birth weight but the *R*^2^ (1.6%) was substantially smaller than the *R*^2^ (3.1%) explained by the score built on the “inclusive set” of 86 SNPs (Table S4 and S13). The new haplotype scores (*S*_h1_, *S*_h2_ and *S*_h3_) showed the same directions of associations with birth outcomes, maternal blood pressure and FPG but less significant p-values, most probably due to smaller instrumental strength of the new scores (Please see tables below and compare with Table 2). Since these new scores showed consistent but less significant results, we intent not including these results in the manuscript to keep it simple.

We recognize that our current finding of “fetal drive” of fetal growth on maternal systolic blood pressure was established based on ALSPAC and HAPO date sets only, also there were problems in blood pressure measurements (see question 1 and 6), modeling of fetal growth (question 4), and the p-value was only modestly significant. In this revision, we discussed these limitations in more detail and highlighted that the validity of this association needs to be confirmed by further replication studies.

| **Genetic score association &**  **Causal estimation** | **Gestational days** | | | **Preterm birth (log(OR))** | | | **Birth weight (g)** | | | **Birth length (cm)** | | |
| --- | --- | --- | --- | --- | --- | --- | --- | --- | --- | --- | --- | --- |
|  | **beta** | **se** | **p-val** | **beta** | **se** | **p-val** | **beta** | **se** | **p-val** | **beta** | **se** | **p-val** |
| *Maternal trans (*$\beta_{h1}$*)* | -0.0069 | 0.003 | **0.032** | 0.0026 | 0.0008 | **0.0007** | 0.67 | 0.1 | **2.20E-11** | 0.0018 | 0.0006 | **0.00094** |
| *Maternal non-trans (*$\beta_{h2}$*)* | -4.10E-05 | 0.003 | 0.99 | -0.0002 | 0.0008 | 0.8 | -0.08 | 0.1 | 0.45 | -0.0001 | 0.0006 | 0.85 |
| *Paternal trans (*$\beta_{h3}$*)* | -0.0075 | 0.003 | **0.017** | 0.0023 | 0.0008 | **0.0034** | 1.1 | 0.1 | **7.90E-29** | 0.0024 | 0.0005 | **1.20E-05** |
| *Maternal effect (*$\beta_{MY}$*)* | 0.00022 | 0.003 | 0.94 | 8.50E-05 | 0.0007 | 0.9 | -0.24 | 0.09 | **0.0045** | -0.0003 | 0.0005 | 0.52 |
| *Fetal effect (*$\beta_{FY}$*)* | -0.0073 | 0.003 | **0.009** | 0.0026 | 0.0007 | **0.0001** | 0.92 | 0.09 | **2.00E-26** | 0.0021 | 0.0005 | **6.90E-06** |
|  |  |  |  |  |  |  |  |  |  |  |  |  |
| *Causal (TSLS)* | -2.04 | 1.03 | **0.048** | 1.05 | 0.365 | **0.0041** |  | | | 1.01 | 0.16 | **3.00E-10** |
| *Maternal trans (*$\beta_{h1}$*)* | -2.47 | 1.04 | **0.018** | 0.738 | 0.257 | **0.004** |  |  |  | 0.778 | 0.185 | **2.60E-05** |

| **Genetic score association**  **&**  **Causal estimation** | **SBP (mmHg)** | | | **DBP (mmHg)** | | | **FPG (mmol/L)** | | |
| --- | --- | --- | --- | --- | --- | --- | --- | --- | --- |
|  | **beta** | **se** | **p-val** | **beta** | **se** | **p-val** | **beta** | **se** | **p-val** |
| *Maternal trans (*$\beta_{h1}$*)* | -0.0033 | 0.002 | 0.16 | -0.0004 | 0.0016 | 0.83 | -0.0001 | 0.0003 | 0.69 |
| *Maternal non-trans (*$\beta_{h2}$*)* | 0.0026 | 0.002 | 0.26 | 0.0023 | 0.0016 | 0.14 | -9.30E-05 | 0.0003 | 0.73 |
| *Paternal trans (*$\beta_{h3}$*)* | 0.0035 | 0.002 | 0.12 | 0.00078 | 0.0016 | 0.62 | 0.00022 | 0.0003 | 0.39 |
| *Maternal effect (*$\beta_{MY}$*)* | -0.0021 | 0.002 | 0.29 | 0.00058 | 0.0014 | 0.67 | -0.00021 | 0.0002 | 0.36 |
| *Fetal effect (*$\beta_{FY}$*)* | -0.0012 | 0.002 | 0.55 | -0.0009 | 0.0014 | 0.51 | 0.00011 | 0.0002 | 0.64 |
|  |  |  |  |  |  |  |  |  |  |
| *Causal (TSLS)* | 1.05 | 0.695 | 0.13 | 0.3 | 0.473 | 0.53 | 0.0651 | 0.075 | 0.39 |
| *Causal (Ratio)* | 1.15 | 0.746 | 0.12 | 0.254 | 0.512 | 0.62 | 0.073 | 0.085 | 0.39 |

3. For height, there was high correlation between the maternal transmitted and non-transmitted GRSs, and the 1d increase in gestational duration per maternal GRS for 6.4cm was found to be offset by an opposite .71d shorter gestation for offspring GRS. Yet, maternal height was still strongly associated with longer gestation (~.9d per 6.4cm, suppl table 3). Any thoughts to wrap these together?

This is a keen observation. Our interpretation of this observation is that large maternal size causally increases duration of gestation; however, the height increase alleles can increase fetal growth when transmitted and in turn shorten gestational duration. We discussed this in the Discussion section:

“*maternal size and fetal growth are important factors in defining the duration of gestation. This is demonstrated not only by the significant causal effect of maternal height (size of the mother) on the duration of gestation, but also by the fact that the maternal or fetal genetic effects that affect fetal growth are usually associated with a reversed effect on gestational duration.*”

We believe that the significant positive correlations between the maternal transmitted and non-transmitted GRSs and also the paternal transmitted height scores (Table S10) are clear evidence of assortative mating by height, which has been by shown previously by us (Zhang et al. PLOS Med 2015; 12:e1001865) and others (Robinson et al. Nat Hum Behav 2017;1:0016). As the three haplotype scores were included in a single regression model (Figure 2) and the correlation was not substantial (<5%), the correlations among the three haplotype scores should not introduce bias nor collinearity problem.

4. Gestational age-adjusted birthweight is a useful indicator of fetal growth in the absence of fetal growth measurements, but several studies reflected its limitations as such. This limitation needs to be acknowledged.

This is a good point. We added discussion of this limitation in this revision.

5. In the Introduction, clarify what “maternal effects” (Introduction, par 1) means in the context of this work. Does it include both genetic and maternal phenotypic effects as conceptualized in Wolf et al’s 2009 paper? Based on the first paragraph and Figure 1, the difference between “maternal phenotype” and “intrauterine environment” is not clear. These terminologies are often used in exchange in the literature, so the authors need to clarify what they mean exactly.

Yes. We followed Wolf et al’s concept in defining “maternal effect”. We explicitly spelled out our definition in this revision (“*maternal effects, defined as the causal influence of the maternal phenotype on birth outcomes or offspring phenotype*”). We removed “genotype” from Wolf et al’s original version (“*the causal influence of the maternal genotype or phenotype*”) to avoid confusion, as all maternal effects are ultimately mediated through maternal phenotypes (including intrauterine environment).

We are sorry for the confusion between “maternal phenotype” and “intrauterine environment”. We deleted “external or intra-uterine” in the introduction. We regard the causal influence of “intrauterine environment” as a maternal effect.

6. In the Methods, clarify how BP was handled for women with more than one (repeat) BP measurements in ALSPAC (30-36wks) and HAPO (24-32wks). Also mention that the GRS for BP was an average of DBP and SBP Scores, as stated in the Table footnote.

The blood pressure in HAPO was a single-time measurement obtained between 24 and 32 weeks of pregnancy when the mothers underwent an oral glucose tolerance test. In ALPAC, the measurement was an average of the multiple blood pressure measured between 30 and 36 weeks. The individual measurements were not available to this study (Please also our response to reviewer 1’s question 3). We clarified that the GRS for BP was an average of DBP and SBP Scores in the Method section.

7. In the abstract, the word “jointly” is confusing - as the tests and not joint tests. Please rephrase. It is also okay to leave out “fetal growth is influenced by both maternal and fetal effects”, to sharpen to focus of the abstract on the new findings.

We deleted “jointly” as suggested by the reviewer.

We kept the “fetal growth is influenced by both maternal and fetal effects”, because these are integrative parts of the “complex maternal and fetal genetic effects” (Figure 6) that we would like to present by this study. Although these results by themselves are not completely novel, our approach provided a more explicit way to model these effects.

8. In the Results, “We only included spontaneous....” is redundant. Delete.

Deleted as suggested.

9. For BP, add “fetal genetic effect” in the sentence related to “94g reduced birthweight…”.

Revised as suggested.
