# Supplementary material for: Dissecting maternal and fetal genetic effects underlying the associations between maternal phenotypes, birth outcomes, and adult phenotypes: A mendelian-randomization and haplotype-based genetic score analysis in 10,734 mother–infant pairs
Source: PLoS Med. 2020 Aug 25;17(8):e1003305. doi: 10.1371/journal.pmed.1003305 (PMC7447062; doi:10.1371/journal.pmed.1003305)
Supplement: Attachment — Submitted filename: response.rev2.docx [file pmed.1003305.s042.docx]

# Requests from the editors:

We appreciate the editors’ very careful attention to our manuscript and have taken all of the comments into account. Our detailed responses follow.

1.Response to editor comments: “Please quote exact p values or p<0.001, unless there are specific reasons to quote smaller p values.”

Your response: We intend to keep the actual exact P values. We understand that expressing P values to more than 3 significant digits usually does not add useful information because P values with extreme results are sensitive to biases or departures from the assumptions of the test. However, in this manuscript, exact P values were used to compare the relative magnitude of the effects of the three haplotype scores tested using the same statistical model.

After consulting with the statistical reviewer, we ask that you please explain this further. Our understanding is that in most cases p-values shouldn’t be directly compared, except perhaps in the context of models where the sample sizes are exactly the same. Can you please clarify the rationale behind direct comparison of p-values and note in the relevant section of the Methods where this is applied to your analyses.

We agree that “in most cases p-values shouldn’t be directly compared, except perhaps in the context of models where the sample sizes are exactly the same”. This is exactly the reason why it is valid to compare the p-values of the three haplotype genetic scores.

The three haplotype genetic scores of the same mother-child pairs were simultaneously tested using a single regression model (i.e. $Y\sim S_{h1}+S_{h2} {+ S}_{h3}+Cov$, where $\mathrm{Cov}$ is a list of appropriate covariates) and therefore they had exactly the same sample size. We revised the relevant section (**Association tests between haplotype genetic scores and pregnancy outcomes, Page 9**) to make this point clear.

“*The three haplotype genetic scores of the same mother-child pairs were simultaneously tested in a single regression model (i.e.* $Y\sim S_{h1}+S_{h2} {+ S}_{h3}+\mathrm{Cov}$*, where* $\mathrm{Cov}$ *is a list of appropriate covariates) and hence they had exactly the same sample size. Therefore, the effect size estimates of these haplotypes scores and their associated p-values can be directly compared to assess the directions and relative contributions of the maternal and fetal effects.*”

2. Response to Reviewer comments: Reviewer 1, point 9: Would you please address this point a bit further, particularly given the well accepted relationship between maternal smoking and fetal effects?

We addressed this as a limitation (Page 22 of the marked up version): “*Another limitation is that due to incomplete data, we were not able to include important environmental or socioeconomic factors in the analysis …*”.

We recognize that some of the maternal environmental or behavioral factors (e.g. smoking) might associate with both maternal phenotypes and birth outcomes, thereby can cause confounding. However, we argue that the genetic scores used in our study are not associated with these factors and therefore our analyses are robust to potential confounding due to these environmental factors. We added additional explanations and two references (*Prev Med*. 2010;50:13 and *Int J Epidemiol*. 2003;32:1) to make this point clearer.

3. Please revise your title according to PLOS Medicine's style. Your title must be nondeclarative and not a question. It should begin with the main concept if possible. Please place the study design ("A randomized controlled trial," "A retrospective study," "A modelling study," etc.) in the subtitle (ie, after a colon). We suggest incorporating that the study included Mendelian randomization as well. We suggest: “Dissecting maternal and fetal genetic contributions to the associations between maternal phenotypes, birth outcomes and adult phenotypes: a Mendelian randomization and haplotype-based genetic score analysis in 10,734 mother-infant pairs of European ancestry” or similar.

According to this suggestion, we revised the title to: “*Dissecting maternal and fetal genetic effects underlying the associations between maternal phenotypes, birth outcomes and adult phenotypes: a Mendelian randomization and haplotype-based genetic score analysis in 10,734 mother-infant pairs*”.

We prefer using “*Dissecting maternal and fetal genetic effects …*” instead of “*Dissecting maternal and fetal genetic contributions …*”, as “maternal effect” has a clear scientific definition, which emphasizes the causal influence of the maternal phenotype on offspring phenotype (*Philos Trans R Soc Lond B Biol Sci*. 2009;364:1107), whereas “maternal contribution” is a broader description, which can include both phenotypic and genetic contributions (by allele transmission). A major advantage of our haplotype-based approach is the capability to distinguish phenotypic vs genetic contributions and to explicitly model maternal and fetal genetic effects and make valid causal inferences. Please also see our response to point 6.

We also had to delete “*of European ancestry*”; otherwise the length of the title will exceed 250 characters.

In the title (and throughout), we suggest using a hyphen for “mother-infant” or “mother-child” pairs.

Modified.

4. Abstract: Background: We suggest revising the last sentence to “The causal mechanisms and the relative contributions of maternal and fetal genetic effects behind these observed associations are unresolved.”

Revised.

5. Abstract: Methods and Findings: We suggest revising to: Please clarify “these observed associations” as it is not clear what this is referencing.

We changed “these observed associations” to “the observed associations between maternal phenotypes and pregnancy outcomes”. According to the suggestion by reviewer 2, we added more descriptions of methods the Methods and findings of the Abstract.

6. Abstract, and throughout: Please revise to avoid causal language, for example in the following sentence. “The maternal non-transmitted haplotype score for height was significantly associated with gestational duration (P=0.00022) and preterm birth (P=0.00097) confirming the causal effect of maternal height on gestational duration.” Similarly, please soften ‘maternal and fetal genetic effects’ to ‘maternal and fetal genetic contributions’ or similar, to avoid causal implications.

We changed the sentence as suggested and revised the abstract to avoid making strong causal implications.

The editors’ suggestion to soften “causal language” throughout the manuscript is well accepted. We followed most of the editors’ suggestions (point 6, 8, 9, 11, 12, 13, and 16) and reduced causal language in all these occasions. However, as the major purpose of this current study is to dissect the maternal and fetal genetic effects underlying the associations between maternal phenotypes, birth outcomes and adult phenotypes, we feel that it is relevant and appropriate to make causal inference. This is particularly applicable to maternal effects and fetal drive – by definition, these are causal influences of maternal phenotypes on birth outcomes (maternal effects) or causal effects of fetal phenotypes on maternal phenotypes (fetal drive). Also, we prefer keeping “maternal and fetal genetic effects” instead of using “maternal and fetal contributions” for the reasons that we proposed in our response to point 3.

7. Abstract, Methods and Findings: If scientific notation is used for p values, please use consistently throughout.

We changed all the p values in the Methods and Findings (Abstract) to scientific notation as suggested. In the rest of the manuscript, we used scientific notation for numbers less than 0.0001 (1.0E-4). We can change all p values to scientific notation if it is recommended by the Editors.

8. Abstract, Methods and Findings: we suggest revising “...however, the glucose-increasing alleles in the fetus reduced birth weight…” to “glucose-increasing alleles in the fetus were associated with reduced birth weight…” or similar, to avoid inferring causality.

Revised.

9. Abstract, Conclusions (and throughout the manuscript): Please revise this paragraph to remove language that implies causality, such as “both maternal height and fetal growth affect the duration of gestation” . Refer to associations instead.

We reduced causal language and used “association” where appropriate.

10. Author Summary: Please place the author summary between the abstract and the introduction sections.

Done.

11. Author Summary: What did the researchers do and find?: Please reduce causal language here.

We avoided using causal language as suggested.

12. Author Summary: What do these findings mean?: Please reduce causal language, particularly in the second bullet point. For the first bullet point, we suggest revising to: “In this study, we observed that maternal size and fetal growth are important factors in shaping the duration of gestation.” or similar.

Revised as suggested.

13. Methods: Construction of genetic scores paragraph on page 6: Please refer to “association” rather than “effect” to avoid causal implications: “To examine the effect of fetal growth (as proxied by birth weight) on pregnancy outcomes and maternal blood pressure and FPG…”

We changed the sentence to “*To examine the associations of fetal growth (as proxied by birth weight) with pregnancy outcomes and maternal blood pressure and FPG …*”

14. Results: Please provide p values associated with all analyses. For example, on page 15, please provide p values associated with the OR for the association between birth weight and susceptibility to T2D (OR = 0.95, 95% CI: 0.92 to 0.99).

We add the p values. We also correct an error in the calculation of odds ratio of T2D: from (OR = 0.95, 95% CI: 0.92 to 0.99) to (OR = 0.91, 95% CI: 0.85 to 0.98, P=0.0073).

15. Discussion: At the beginning of the discussion, please summarize your findings in paragraph form rather than as a numbered list.

We put these points in a paragraph as suggested.

16. Discussion: Please temper the causal language. Under the first paragraph, in point 1, we suggest revising the final sentence to: “These new results support our previous finding [15] with additional evidence.”

We revised the sentence as suggested and we soften the language of causal inference.

17. Discussion: Final paragraph: We suggest revising this sentence to: “These maternal and fetal genetic effects may explain the observed associations between the studied maternal phenotypes and birth outcomes as well as the life-course associations between these birth outcomes and adult phenotypes.”

Revised as suggested.

18. Tables: Please define abbreviations for “OR” and “se” in the legends.

Added.

19. Figure 3, 4, 5, 6: Please define abbreviations for FPG, BMI, BP, T2D in the figure legend.

Added.

20. Supporting Information Tables: Please define all abbreviations, such as for SBP, DBP, FPG, BMI, se, sd in the legends, where appropriate

Added.

# Comments from the reviewers:

## Reviewer #1:

The authors have done a thorough job revising their manuscript. Appreciate the thought given to the responses and providing additional analyses. Given the number of the figures and tables already included and supplemental analyses, I would try to refrain any more further analyses as there already 5-6 key findings from the analyses. As with these types of studies, there could be even more analyses and permutations but it would be advisable at this stage to keep this to a minimum. I think any further analyses could be highlighted more as future research in the discussion. The only point I would add is that, perhaps in the discussion is that although the analysis was done as a cross-sectional study, the nature of the data used involves several birth cohorts which longitudinal data which opens up some future opportunities to explore later growth outcomes, including the relationship between glucose raising alleles on lower birth weight and how this relates to rapid growth in infancy (as we do observe LBW children tend to exhibit a period of rapid catch up growth as well in their first 1-2 years of life). This interplay between genetics, fetal growth, low birth rate, and subsequent growth in the first years of life would have potential to be answered using these same datasets the authors have collated. Food for thought in future exploration in more longitudinal designs.

We are very pleased to know that the reviewer is satisfied with our responses and the revisions that we have made. We thank the reviewer again for the expert comments and advise.

We thank the reviewer for pointing out the future opportunities of conducting similar analysis using longitudinal data. We added the following sentences at the end of the Discussion section (Page 23 of the marked up version) to encourage future exploration in more longitudinal designs:

“*Although our current study focused on pregnancy outcomes measured at birth (e.g. gestational duration and birth weight), similar analysis can be conducted on longitudinal measures (e.g. those related to growth-pattern in early life and later adulthood). With the accumulation of more longitudinal birth cohorts, the dissection of maternal and fetal genetic effects may open up future opportunities to explore how maternal effect, fetal development and genetics influence long-term health and well-being.*”

## Reviewer #2:

I thank the authors for their careful consideration of the previous comments regarding the proposed manuscript. I feel that they addressed the majority of my concerns in the updated manuscript. I do have a few minor suggestions.

We thank the reviewer for the thoughtful comments, which helped us to improve the quality of the manuscript.

I feel that the Methods/Results section is very results heavy. There is a single sentence form methods and the rest is dedicated to results. However, I feel given the complexity of the methodology a few more details would be welcome here. I think you can focus on the main findings rather than all of the results.

We revised the Methods and findings of the Abstract. We added several sentences to explain the methods and reduced the descriptions of results.

Also, there is not Author Summary included as indicated in their response. I think this would be useful for a clinical audience.

We uploaded Author Summary as a separate file in the previous submission. We now put the Author Summary between the Abstract and the Introduction sections.

## Reviewer #4:

All my comments have been adequately addressed.

We are very pleased to know that the reviewer is satisfied with our responses.
